# Advanced High‐Throughput Rational Design of Porphyrin‐Sensitized Solar Cells Using Interpretable Machine Learning

**DOI:** 10.1002/advs.202407235

**Published:** 2024-09-24

**Authors:** Jian‐Ming Liao, Yu‐Hsuan Chen, Hsuan‐Wei Lee, Bo‐Cheng Guo, Po‐Cheng Su, Lun‐Hong Wang, Nagannagari Masi Reddy, Aswani Yella, Zhao‐Jie Zhang, Chuan‐Yung Chang, Chia‐Yuan Chen, Shaik M Zakeeruddin, Hui‐Hsu Gavin Tsai, Chen‐Yu Yeh, Michael Grätzel

**Affiliations:** ^1^ Department of Chemistry National Central University No. 300, Zhongda Rd., Zhongli District Taoyuan City 32001 Taiwan; ^2^ Department of Chemistry i‐Center for Advanced Science and Technology (i‐CAST) Innovation and Development Center of Sustainable Agriculture (IDCSA) National Chung Hsing University Taichung City 402 Taiwan; ^3^ Laboratory for Photonics and Interfaces Institute of Chemical Sciences and Engineering École Polytechnique Fédérale de Lausanne Lausanne 1015 Switzerland; ^4^ Research Center of New Generation Light Driven Photovoltaic Modules, National Central University Taoyuan 32001 Taiwan

**Keywords:** design rules, dye‐sensitized solar cells, high‐throughput virtual screening, interpretable machine learning model, SHAP

## Abstract

Accurately predicting the power conversion efficiency (PCE) in dye‐sensitized solar cells (DSSCs) represents a crucial challenge, one that is pivotal for the high throughput rational design and screening of promising dye sensitizers. This study presents precise, predictive, and interpretable machine learning (ML) models specifically designed for Zn‐porphyrin‐sensitized solar cells. The model leverages theoretically computable, effective, and reusable molecular descriptors (MDs) to address this challenge. The models achieve excellent performance on a “blind test” of 17 newly designed cells, with a mean absolute error (*MAE*) of 1.02%. Notably, 10 dyes are predicted within a 1% error margin. These results validate the ML models and their importance in exploring uncharted chemical spaces of Zn‐porphyrins. SHAP analysis identifies crucial MDs that align well with experimental observations, providing valuable chemical guidelines for the rational design of dyes in DSSCs. These predictive ML models enable efficient in silico screening, significantly reducing analysis time for photovoltaic cells. Promising Zn‐porphyrin‐based dyes with exceptional PCE are identified, facilitating high‐throughput virtual screening. The prediction tool is publicly accessible at https://ai‐meta.chem.ncu.edu.tw/dsc‐meta.

## Graphical abstract

Innovative High‐Throughput Design of Porphyrin‐Sensitized Solar Cells Through Interpretable Machine Learning

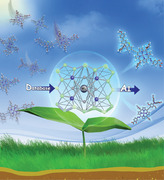

## 1 Introduction

DSSCs, designed to utilize solar energy, have garnered significant attention in addressing the growing need for clean and renewable energy sources, due to their simple fabrication, cost‐effectiveness, and suitability when compared to traditional silicon (Si)‐based photovoltaic devices.^[^ ^1^ ^]^ They are particularly well‐suited for applications in indoor settings and building‐integrated photovoltaic (BIPV) systems.^[^ ^1^, ^2^, ^3^ ^]^ After three decades of development, the highest achieved PCE for DSSCs stands at 14.3% under standard air mass 1.5 global (AM 1.5G) illumination,^[^ ^4^ ^]^ and 32% under artificial indoor lighting conditions.^[^ ^2^ ^]^ Among various sensitizers, porphyrins^[^ ^3^, ^5^, ^6^, ^7^ ^]^ stand out as prominent contenders, owing to their unique attributes, including strong absorption across a broad spectral range, tunable photophysical and electrochemical properties, and the ability to sustain long‐lived excited states, which facilitate efficient electron injection processes. Notably, porphyrin and their derivatives exhibit prominent Soret (B) bands in the wavelength range of 400 to 500 nm, along with moderate Q bands spanning 500 to 700 nm, and demonstrate enhanced absorption in the near‐infrared (NIR) region, exemplifying their remarkable panchromatic light‐harvesting capability.^[^ ^5^ ^]^ Moreover, the structural flexibility of porphyrin enables fine‐tuning of the photophysical, electronic, and photovoltaic properties of porphyrin sensitizers, ultimately tailoring their overall performance in DSSCs. In 2011, **YD2‐*o*‐C8**, developed by Yeh and workers,^[^ ^3^ ^]^ was cosensitized with an organic dye, **Y123**, and used a Co(II/III)‐based redox electrolyte, performing a record PCE of 12.3%. In 2014, **GY50** and **SM315** achieved a PCE of 12.75% and 13.0%, improving the PCE by introducing benzothiadiazole to porphyrin.^[^ ^3^, ^8^ ^]^ Recently, Grätzel and his collaborators have achieved a remarkable advancement for organic dye, **SL9**, elevating the PCE record further to 15.2%.^[^ ^9^ ^]^

Further improving DSSCs' efficiency demands strategic design and synthesis of novel, high‐efficiency dye sensitizers, crucial for light absorption and electron generation.^[^ ^10^, ^11^ ^]^ The vast chemical space of potential dye molecules, with numerous functional groups and diverse combinations, poses challenges in terms of laborious and costly synthesis and purification procedures to find optimal dyes from such a huge chemical space. For DSSCs with complex processes, a reliable multidimensional quantitative structure‐property relationship (QSPR) is highly desirable to expedite the discovery of high‐performance dye sensitizers, despite the application of general design principles. Such a model would facilitate cost‐effective high throughput virtual screening of candidate dyes, reducing the reliance on time‐consuming experimental approaches. However, the development of multidimensional QSPR models for DSSCs faces significant hindrances due to the complex chemical processes in DSSC devices. This entails not only considering the ground‐state and excited‐state photochemical properties of dyes but also accounting for the intermolecular interactions of DSSC devices. As a result, quantitative virtual screening for various dye‐sensitizers in DSSC applications remains challenging.^[^ ^12^, ^13^, ^14^, ^15^, ^16^ ^]^

ML established multidimensional QSPRs recently emerged as a promising data‐driven technique for expediting the discovery of novel materials.^[^ ^17^, ^18^, ^19^, ^20^, ^21^ ^]^ By establishing correlations between the diverse physical and chemical properties of studied materials and their desired properties, ML enables efficient material design. Numerous ML models applied in material design have been reported, spanning various domains such as organic light‐emitting diodes,^[^ ^22^ ^]^ perovskites,^[^ ^23^, ^24^, ^25^, ^26^, ^27^ ^]^ and organic photovoltaic materials.^[^ ^13^, ^20^, ^21^, ^28^ ^]^ These examples highlight the potential of ML as a valuable tool for accelerating material discovery and development. Recently, ML models have also been developed for photovoltaic devices.^[^ ^12^, ^13^, ^14^, ^15^, ^29^ ^]^ Remarkably, Ma and colleagues have successfully developed predictive ML models employing molecular descriptors (MD) derived from density functional theory (DFT) calculations. The stacking learners demonstrates exceptional performance, excelling in both robustness and accuracy, with validation and testing set correlation coefficients (*r*_val_/*r*_test_) of 0.79 and 0.78, respectively. Their developed workflow ensures both effectiveness and expediency in discovering promising organic dyes.^[^ ^13^ ^]^ Moreover, Liu et al.^[^ ^23^ ^]^ leveraged ML algorithms and a curated dataset consisting of 814 valid data points extracted from 2735 peer‐reviewed publications. Their focus was on building ML prediction models for critical properties of perovskites, including bandgap, conduction band minimum, valence band maximum, and electrical parameters of perovskite solar cells (PSCs). Additionally, they employed the Shapley Additive Explanations (SHAP) theory^[^ ^30^, ^31^ ^]^ to analyze and elucidate the significant factors influencing the PCE of PSCs. These recent studies exemplify the growing significance of ML‐based methodologies in materials discovery and design, showcasing their potential in accelerating advancements in various fields of solar cell research. Remarkably, Lee has developed a predictive ML model for accurately estimating the efficiency of ternary organic solar cells (OSCs) that utilize a nonfullerene acceptor.^[^ ^21^ ^]^ This model achieves a high level of accuracy, with a coefficient of determination exceeding 0.9, while employing a reduced number of MDs.

To expedite the discovery of dye sensitizers by ML, the primary focus is to assess the capability of ML models in navigating unexplored chemical spaces. In the context of having limited data points, which may fall short of the widely advocated “big data” standard, the selection of effective MDs becomes a critical task as they define the chemical space that ML models can explore. Given that DSSCs primarily involve electron transfer processes, it is anticipated that MDs derived from first‐principle molecular orbital calculations^[^ ^13^ ^]^ can effectively expand the known chemical space (training set) to encompass unexplored regions (*de novo* dyes), in contrast to MDs‐based solely on molecular fingerprints. Moreover, a pivotal aspect of a successful predictive ML model lies in its interpretability, allowing it to provide meaningful physical‐chemical insights and formulate design rules, rather than being limited to an opaque black box.^[^ ^32^ ^]^ First‐principle calculated MDs provide valuable physics‐based insights that can be utilized to establish chemical guidelines for the transparent and enlightening design and optimization of dye sensitizers, making them suitable for extensive virtual screening. Furthermore, it is common practice to conduct a test study using either published experimental data points to assess the performance of ML models. However, it is essential to emphasize the importance of testing data similarity to the training data when evaluating the ML predictions for uncharted chemical spaces.

**Scheme 1:**
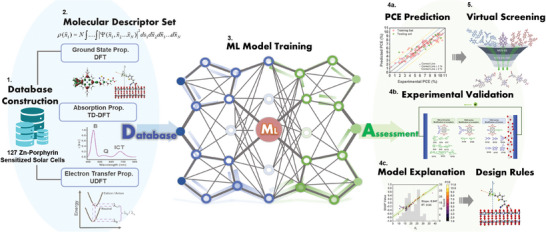
The workflow employed in this study.

In light of the aforementioned challenges, our primary aim is to construct a reliable, predictive and interpretable ML model for *meso*‐functionalized Zn‐porphyrin dyes with a push‐pull D‐π‐A framework, for high throughput rational design in DSSCs. This model will enable a more comprehensive understanding of the multidimensional QSARs and pave the virtual screening for efficient and targeted discovery of promising Zn‐porphyrin dyes for enhanced DSSC performance. To achieve this, we integrate ML techniques with physical and chemical properties derived from first principle quantum chemistry calculations, aligning with DSSC working principles. The process flow of our framework is illustrated in **Scheme** **1**. First, we commence by curating a database comprising 127 peer‐reviewed Zn‐porphyrin dyes (Table S1, Supporting Information), encompassing experimentally determined PCE ranging from 0.3% to 10.51%. Next, three levels of complexity for MD Sets (MDS) are constructed (Tables S2–S4, Supporting Information), incorporating ground‐state (MDS−GS), absorption (MDS−ABS), and electron transfer (ET, MDS−ET) properties of dyes, obtained through DFT, time‐dependent DFT (TD‐DFT), and unrestricted DFT (UDFT) calculations, respectively. The three MD Sets (47 MDs) are carefully chosen and assembled to represent the impact of Zn‐porphyrins on DSSC's PCE, serving as inputs for our ML models, which encompass light gradient boosting machine (LGBM), artificial neural network (ANN), and convolutional neural network (CNN). The purpose is to establish meaningful correlations between these MD Sets and the experimental PCE. With respect to SHAP values,^[^ ^30^, ^31^ ^]^ valuable chemical guidelines are extracted to inform dye design. We evaluated the predictive capability of our optimized ML models using a set of 17 newly designed Zn‐porphyrin‐sensitized solar cells that had not been previously reported in the literature. Our ML model provided excellent predictions for their PCEs, closely matching the experimentally measured values. This independent validation underscores the accuracy of our approach in navigating the unexplored chemical space of porphyrin dyes. The optimized ML model predicts the PCE of 3084 automatically generated Zn‐porphyrin dyes using two computationally inexpensive MDs from the training set. Promising Zn‐porphyrin‐based dyes with exceptional PCE are identified. Our findings show that MDs beyond isolated molecule calculations enhance PCE prediction, and our computational workflow ensures efficient and rapid discovery of promising Zn‐porphyrin dyes from a vast chemical space.

## 2 Experimental Section

### 2.1 Database Construction and Data Division

A dataset comprising 127 Zn‐Porphyrin‐sensitized solar cells (Table S1, Supporting Information) was assembled from various literature sources and served as the foundation for training the ML models. The Zn‐Porphyrins in the database generally possess a push‐pull framework, with donor functionalized at the *meso*‐position and acceptor functionalized at the opposite *meso*‐position. Furthermore, their *β*‐positions are not functionalized. To ensure reasonable comparability of the data, specific selection criteria were applied, despite their diverse origins: *i*) utilization of the I^−^/I_3_^−^ electrolyte; *ii*) employment of TiO_2_ semiconductor; *iii*) absence of polymer, co‐sensitizer, or co‐adsorbent usage; and *iv*) acquisition of PCE data under the standardized AM 1.5G light source with a total irradiance of 100 mW cm^−2^. The utilization of I^−^/I_3_^−^ electrolytes in Zn‐porphyrin‐based DSSCs had provided a more extensive dataset, enabling the training of the ML model. Figure S1, Supporting Information illustrates the PCE distribution (0.1–10.5%) of Zn‐Porphyrin‐sensitized solar cells within the database. This database consisted of low‐medium‐high PCE data points. The application of these stringent criteria guaranteed the validity and comparability of the data, facilitating reliable analysis and ML model training.

### 2.2 Molecular Descriptors and Molecular Descriptor Sets

In this study, a meticulous curation of 47 MDs was undertaken, employing the foundational principles expounded in the supporting materials. These MDs were primarily computed through the utilization of DFT, TD‐DFT, and UDFT calculations. The overarching aim of this effort was to facilitate expedited multi‐stage virtual screening of novel Zn‐porphyrin compounds. To this end, this work had systematically organized these 47 MDs into three distinct sets, referred to as MDS, predicated upon their inherent properties and the computational approaches employed for their derivation. Importantly, it was worth noting that each of these three MD Sets can be derived from a solitary DFT, TD‐DFT, or UDFT calculation. The envisaged utility of these three MD Sets lied in their ability to collectively simulate varying levels of complexity inherent to DSSC processes. For a concise overview of these MDs and their respective descriptions, this work direct readers to Tables S2–S4, Supporting Information. The following elucidation provides a categorization of the three MD Sets for clarity:

- MDS−GS (MD 1–23, Table S2, Supporting Information): This set encompassed MDs primarily derived from ground‐state properties, chiefly acquired through a DFT geometry optimization computation.

The MDS−GS comprised six frontier molecular orbitals (MOs): the three highest occupied MOs and the three lowest unoccupied MOs, alongside the energy differentials between these occupied and unoccupied MOs. Meanwhile, MD 16 denoted the dye's dipole moment (DM). MDs 17–21 elucidated the structural attributes of the dye. These encompassed the degree of unsaturation,^[^ ^33^ ^]^ the degree of freedom with alkyl chains replaced by methyl, and the count of carbon atoms in the alkyl chains attached to donor, aryl at the *meso*‐position (*meso*‐aryl), and acceptor, etc. All quantum chemistry calculations, except for MDs 22 and 23, were performed on individual Zn‐porphyrin molecules in an implicit solvent using the C‐PCM model. The details are provided in the SI.

This work had introduced two new MDs that were derived from the binding mode of dyes on TiO_2_, namely, *ϕ* (MD 23) and *d_z_*_._ (MD 22). *ϕ* represents the tilt angle at which the dye is adsorbed on TiO_2_ concerning the normal axis of an anatase TiO_2_ (101) surface, while *d_z_* signifies the vertical distance between the electron donor of the dye and the TiO_2_ surface. The manner in which sensitizers adsorb to the surface of TiO_2_ in DSSCs played a pivotal role in shaping their overall performance.^[^ ^34^, ^35^, ^36^, ^37^, ^38^, ^39^, ^40^, ^41^ ^]^ Dyes that adopt an optimal tilt angle exhibited enhanced efficiency in light absorption and charge separation processes. A well‐suited tilt angle ensured more effective coverage of the TiO_2_ surface, thereby facilitating the efficient utilization of incident photons. This, in turn, lead to an increased dye loading, a desirable characteristic for augmenting light‐harvesting efficiency. Dyes with specific tilt angles can affect charge recombination by modulating the distance between the oxidized dye's hole and the TiO_2_ surface. This modulation was of paramount importance as it helped in sustaining a prolonged electron lifetime, ultimately contributing to an enhancement in the overall efficiency of the DSSC.

**Figure 1:**
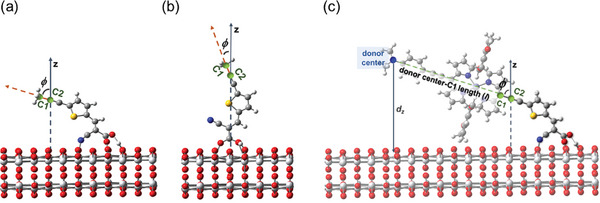
Illustrates the tilt angle of the cyanoacrylic anchor group when adsorbed onto a TiO_2_ surface. The anchor group assumes different configurations: a) tridentate fashion, b) bidentate fashion, and c) in this graph, we present the approximations employed to ascertain the tilt angle and vertical distance of a donor center for a specific Zn‐porphyrin dye upon adsorption onto a TiO_2_ surface.

**Figure** **1** demonstrates and defines the *ϕ* and *d_z_*_._ To model *ϕ* and *d_z_*_._ of dye adsorbed on TiO_2_. One of the key functions of ML models was to enable high‐throughput virtual screening. Performing DFT geometry optimizations on a large number of Zn‐porphyrin adsorbed on TiO_2_ systems was challenging; however, this model‐building approach effectively addressed this challenge. Instead of calculating the entire dye molecule adsorbed on TiO_2_, this wok estimated the *ϕ* and *d_z_*_._ of the dye based on the configuration of dye's anchor adsorbed on TiO_2_ and the geometry of the isolated dye. This work first optimized 54 kinds of acceptors of the database adsorbed on (TiO_2_)_64_ unit cell with anatase TiO_2_ (101) surface under periodic boundary condition based on the DFT. The acceptors were capped by a methyl group. On the basis of the previous study^[^ ^42^ ^]^ of the adsorption models of cyanoacrylic and carboxylate anchored on TiO_2_, the carboxylate anchor adopted a bidentate fashion, while the cyanoacrylic anchor adopted the most stable tridentate via COO^−^ and CN groups and H^+^ on TiO_2_ [see Figure 1a, coordinates are provided in Table S10, Supporting Information] and kinetically‐trapped bidentate fashion via COO^−^ group, [see Figure 1b, coordinates are provided in Table S11, Supporting Information]. For dyes featuring the cyanoacrylic anchor, the determination of their tilt angle involved considering an average of the tilt angle observed in both bidentate and tridentate binding modes. The tilt angle for a specific anchor was calculated as following. Initially, this work established a vector aligned with the carbon atom (C1) of the capped methyl group and its adjacent non‐hydrogen atom (C2). This vector ($\underset{C2C1}{\to }$) was calculated based on the optimized structural configuration of the specific anchor adsorbed onto the TiO_2_ substrate. Subsequently, the tilt angle of the particular anchor concerning the normal axis of the TiO*_2_* surface can be computed. This work approximated the tilt angle of a dye on a TiO_2_ substrate by correlating it with the tilt angle of its anchor, as determined previously. This approximation significantly expedited the virtual screening process for a diverse range of candidate dyes, given that the geometry optimization of Zn‐porphyrin adsorbed on a TiO_2_ substrate incurs computational costs. The rationale for this approximation was rooted in the following observations and assumptions. In the database, it was observed that the Zn atom, the *meso*‐carbon atom, and the adjacent atoms within the anchor groups of the optimized Zn‐porphyrins tend to align linearly. Consequently, considering the capped C1 atom within the anchor as the *meso*‐carbon of a given Zn‐porphyrin within the database, and superimposing its anchor group onto the adsorbed structure of its anchor on the TiO_2_ surface, this work discerned that the vector $\underset{C2C1}{\to }$ traverses through the center, such as the Zn atom, of the porphyrin macrocycle and also the opposing *meso*‐carbon atom [Figure 1c]. In this context, the tilt angle of the anchor adsorbed was employed on the TiO_2_ surface as a representative measure for the tilt angle of the corresponding Zn‐porphyrins.

To estimate the *d_z_* for a specific dye, this work followed a methodical procedure. Initially, this work determined the length (*l*) between the *meso*‐carbon atom and the defined donor center based on the optimized geometry of the dye. Subsequently, the distance between the defined donor center and the C2 atom was calculated, as illustrated in Figure 1c (coordinates are provided in Table S12, Supporting Information). The calculation of the *z*‐coordinate of the defined donor center for a dye adsorbed on a TiO_2_ surface, and thereby the determination of *d_z_*, relies on the knowledge of the tilt angle and the distance between the defined donor center and the C2 atom. The definition of the donor center was governed by the following rules: 1). If a donor group contained a nitrogen atom, the nitrogen atom was designated as the donor center; 2). If a donor group comprised more than one nitrogen atom, a non‐hydrogen atom in close proximity to the geometric center of the nitrogen atoms was assigned as the donor center; and 3). In cases where a donor group lacks any nitrogen atom, this work defined one of the carbon atoms nearest to the geometric center of the donor group as the donor center. This systematic approach ensured the consistent determination of *d_z_* for dyes adsorbed on TiO_2_ surfaces, taking into account the specific donor characteristics of each dye. For a large‐scale virtual screening of candidates, these approaches were computationally efficient by reusing the pre‐calculated configuration of the dye's anchor adsorbed on TiO_2_.

- MDS−ABS (MDs 24–42, Table S3, Supporting Information): MDS−ABS encompassed MDs associated with the properties of dye absorption spectra. These descriptors predominantly arose from computations involving the absorption spectra using the TD‐DFT methodology.

MDS−ET (MDs 43–47, Table S4, Supporting Information): MDS−ET encompassed the MDS primarily related to the internal reorganization energy of dyes. These descriptors were obtained through UDFT calculations that involve geometry‐optimized cationic and anionic species. Additionally, properties such as the kinetics of electron injection, based on Marcus' theory,^[^ ^43^, ^44^ ^]^ which were derived from the characteristics of cationic and anionic species, were included in this MD set (see the SI for details). In addition, energy difference between singly occupied MO (SOMO) of cationic dye and electrolyte's (I^−^/I_3_^−^) oxidation potential was also included.

### 2.3 Synthesis of GY‐Series Dye

**Figure 2:**
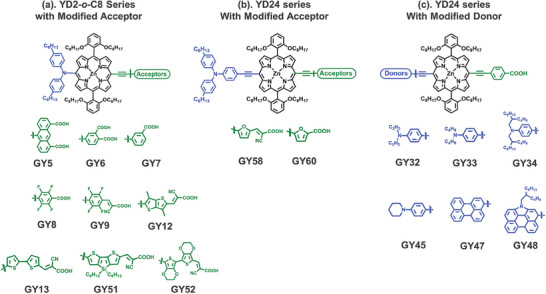
17 newly designed Zn‐porphyrin sensitizers. a) 9 Zn‐porphyrin molecules derived from **YD2‐*o*‐C8** framework with diverse donor variations; b) 2 Zn‐porphyrin molecules based on **YD24** framework with donor group modifications; and c) 6 Zn‐porphyrin molecules from **YD24** framework, with different acceptor groups. The experimental measured PCE value is indicated in parentheses.

**Scheme 2:**
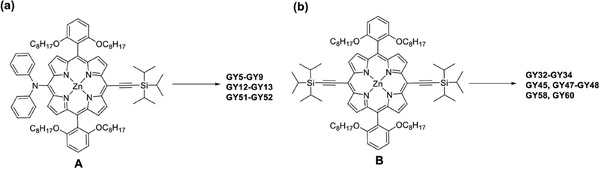
Synthesis Routes for **GY**‐series Porphyrin Dyes.

Seventeen newly designed Zn‐porphyrin dyes were synthesized (**Figure** **2**). The synthesis route for these 17 **GY**‐series Zn‐porphyrin dyes is depicted in **Scheme** **2**. This section detailed the synthesis method for **GY58** as a representative example. To a solution of B (Scheme 2, 131.4 mg, 0.093 mmol) in THF (3 mL) was added tetra(n‐butyl)ammonium fluoride (0.37 mL, 0.71 mmol) at room temperature. Before water was added, the mixture was stirred for 1 h. The solution was extracted with dichloromethane, the solvent was evaporated under reduced pressure to get the deprotected intermediate. To a solution of the deprotected intermediate in a degassed mixture of THF (10 mL) and Et_3_N (2 mL) was added 2‐cyano‐3‐(5‐iodothiophen‐2‐yl)acrylic acid (42.1 mg, 0.138 mmol), 4‐hexyl‐N‐(4‐hexylphenyl)‐N‐(4‐iodophenyl)benzenamine (59.7 mg, 0.110 mmol), Pd_2_(dba)_3_ (8.4 mg, 0.009 mmol), and AsPh_3_ (28.2 mg, 0.092 mmol). The mixture was refluxed for 4 h. The solvent was removed under vacuum and the residue was purified by column chromatography (silica gel) using CH_2_Cl_2_/CH_3_OH (15/1) as eluent to afford **GY58**.

The synthesis of porphyrin sensitizers **GY60**, **GY32‐34**, **GY45**, **GY47‐48** followed similar routes as that of **GY58**. Likewise, the synthesis of porphyrin sensitizers **GY5‐9**, **GY12‐13**, and **GY51‐52** used A (Scheme 2) as the starting material, following similar routes to that of **GY58**. the chemical structures were characterized by ^1^H and ^13^C NMR and MS (see the SI).

### 2.4 Device Fabrication

The^[^ ^3^ ^]^ TiO_2_ electrodes comprising a 5 µm mesoporous TiO_2_ layer (particle size, 20 nm, pore size 32 nm) and a 5 µm TiO_2_ scattering layer (particle size, 400 nm) used as photoanode. The working electrode was prepared by immersing the 10 µm (5 µm thick transparent layer +5 µm thick scattering layer) TiO_2_ film into dye solution for 18 h. A thermally platinized FTO glass counter electrode and the working electrode were then sealed with a 25 µm thick hot‐melt film (Surlyn), by heating the system at 100 °C. Devices were completed by filling the electrolyte by pre‐drilled holes in the counter electrodes and finally the holes were sealed with a Surlyn sheet and a thin glass cover by heating. A black mask (6×6 mm^2^) was used in subsequent photovoltaic studies. The used electrolyte was prepared by 1.0 m 1,3‐dimethylimidazolium iodide (DMII), 0.03 m iodine, 0.1 m guanidinium thiocyanate and 0.5 m *tert*‐butylpyridine in a mixture of valeronitrile/acetonitrile (15:85 v/v).

## 3 Results and Discussion

### 3.1 Performance of Predictive Models in Terms of MDS−GS

**Figure 3:**
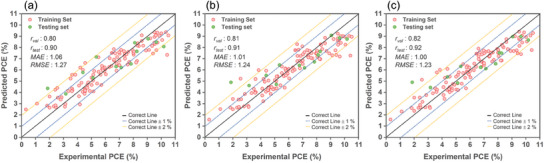
The performance assessment and predicted PCE values from MDS‐GS trained models using a) LGBM, b) ANN, and c) CNN are compared with the experimentally measured PCEs for both the training and test sets, utilizing the bagging technique.

**Figure** **3** shows the performance and comparison of predicted PCE values from ML models trained on MDS‐GS using three methods: LGBM, ANN, and CNN. The comparison includes both the experimentally measured PCEs for the training and test sets, using the bagging technique. The MDS‐GS primarily includes MDs derived from ground‐state properties.

The CNN model achieved the highest validation correlation coefficient (*r*_val_), with a value of 0.82, indicating a strong positive relationship between predicted and experimental values. The LGBM model demonstrated the highest testing correlation coefficient (*r*_test_) at 0.90 in the testing phase, implying excellent predictive performance on unseen data. All three learners exhibited an *MAE* slightly above 1.09%, indicating their capability to predict the PCE of new Zn‐porphyrin‐sensitized solar cells with an error of ≈1.09%. Among these models, the CNN model stood out with the lowest *MAE*, specifically 1.09. This implies that, on average, its predictions deviated by only 1.09% from the actual values.

The analysis reveals a common trend among the three models. Most of the data points in the higher PCE region (> 8%) are consistently under‐predicted. Conversely, in the low PCE region (< 4%), most data points tend to be over‐predicted. These outcomes may be attributed to the substantial influence of the most prevalent medium‐range PCE data points.

**Figure 4:**
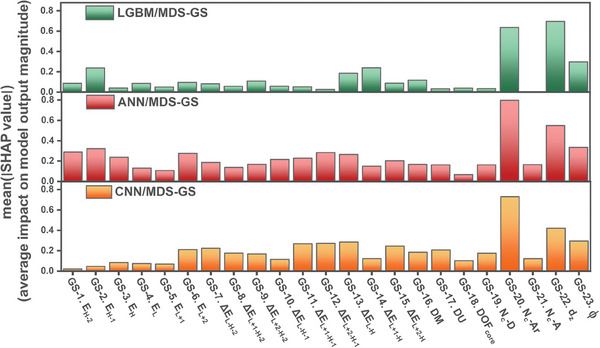
MD importance based on SHAP values for a) LGBM/MDS‐GS, b) ANN/MDS‐GS, and c) CNN/MDS‐GS models.

**Figure** **4** illustrates the MD importance based on SHAP values determined by models trained on the MDS−GS. While the importance of MDs varies across the three models, they do share some consistent significance, which includes *N_C_*‐Ar (number of alkyl chain's carbon atoms attached to *meso*‐aryl groups), *d_z_* (vertical distance of the donor center of dye adsorbed on TiO_2_ to TiO_2_ surface), and *ϕ* (tilt angle of dye).

### 3.2 Performance of Predictive Models in Terms of MDS−GS+ABS

**Figure 5:**
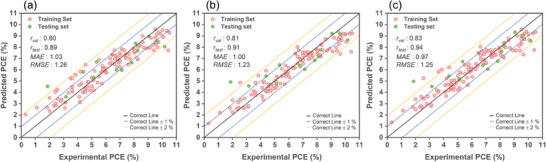
The performance assessment and predicted PCE values, based on the MDS−GS+ABS trained models using a) LGBM, b) ANN, and c) CNN learners, are compared to the experimentally measured PCEs for both the training set and test set after applying the bagging technique.

**Figure** **5** shows the performance assessment and predicted PCE values from MDS−GS+ABS trained models (LGBM, ANN, and CNN) compared to experimental PCEs. The MDS‐GS+ABS set includes 23 MDs from MDS−GS and 19 additional MDs from TD‐DFT excited‐state calculations in MDS−ABS. When evaluated with MDS−GS+ABS, all three models yield smaller *MAE* and *RMSE* values and larger *r*_tval_ and *r*_test_ values compared to their counterparts using MDS−GS alone, suggesting that the inclusion of MDs pertaining to dye's absorption spectra enhances prediction accuracy. Notably, the CNN/MDS−GS+ABS model boasts a particularly low *MAE* of 1.00%.

**Figure 6:**
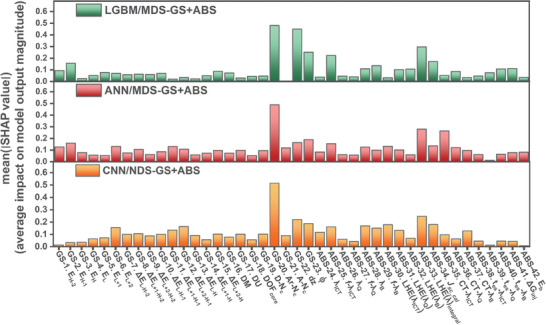
MD importance based on SHAP values for a) LGBM/MDS−GS+ABS, b) ANN/MDS−GS+ABS, and c) CNN/MDS−GS+ABS models.

**Figure** **6** shows the importance of MDs as determined by SHAP values, using models trained on the MDS−GS+ABS set. The key MDs identified as most significant, namely *N_C_*‐Ar, *d_z_*, and *ϕ* , in the MDS−GS models, continue to maintain their prominence in the MDS−GS+ABS models. Furthermore, it is noteworthy that the light‐harvesting efficiency integral (LHE(λ)_integral_, in MD−ABS) emerged as a top‐ranked MD in the LGBM, ANN, and CNN models.

### 3.3 Performance of Predictive Models in Terms of MDS−GS+ABS+ET

**Figure 7:**
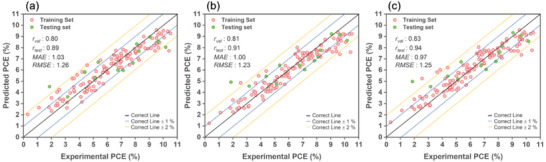
The performance assessment and predicted PCE values, based on the MDS−GS+ABS+ET trained models using a) LGBM, b) ANN, and c) CNN learners, are compared against the experimentally measured PCEs for both the training set and test set after applying the bagging technique.

**Figure** **7** presents three plots comparing the performance assessment and predicted PCE values from MDS−GS+ABS+ET trained models (LGBM, ANN, CNN) to the experimentally measured PCE values. The MDS−GS+ABS+ET comprises MDS−GS+ABS, supplemented with an additional 5 MDs from MDS−ET, primarily focusing on reorganization energy calculations derived from UDFT computations involving dyes' cationic and anionic states. The overall performance of these three models, when evaluated using MDS−GS+ABS+ET, surpasses that of their counterparts employing MDS−GS. Notably, their performance is slightly enhanced compared to their counterpart models utilizing MDS−GS+ABS.

**Figure 8:**
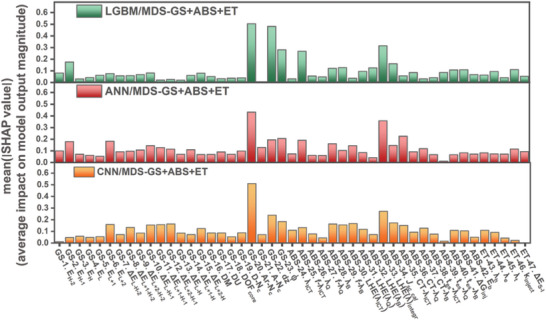
MD importance based on SHAP values for a) LGBM/MDS−GS+ABS+ET, b) ANN/MDS−GS+ABS+ET, and c) CNN/MDS−GS+ABS+ET models.

The significance of MDs as inferred from SHAP values, is illustrated in **Figure** **8**. It is noteworthy that four key MDs, specifically *N_C_*‐Ar, *d_z_*, *ϕ* , and LHE(λ)_integral_, which were prominent in the MDS−GS and MDS−GS+ABS models, continue to maintain their importance in the context of the extended MDS−GS+ABS+ET models in terms of using LGBM, ANN, and CNN learners. Nevertheless, the five MDs in the MDS−ET model do not make substantial contributions to the models using MDS−GS+ABS+ET.

### 3.4 Insights into Role of Key Molecular Descriptors for Chemical Design Rules

Our study shows the three learners in conjunction with three different MD Sets share common important MDs: *N_C_*‐Ar, *d_z_*, *ϕ*, and LHE(λ)_integral_. To analyze the MD importance, we examine the MD importance of the optimized CNN/MDS−GS+ABS+ET predictive model based on SHAP values with respect to the calculated properties. The SHAP plots provide an opportunity to explore established concepts related to the relationship between PCE and molecular properties.

#### 3.4.1 Effects of Location and the Number of Carbon Atoms of Alkyl Chains

It has been recognized that the presence of alkyl chains in dyes acts as a protective layer, preventing the aggregation of sensitizers and reducing charge recombination on the TiO_2_ surface, ultimately enhancing the performance of DSSCs.^[^ ^45^, ^46^, ^47^ ^]^ In this context, the specific locations on the dye molecule where the long alkyl chains are attached play a crucial role in promoting PCE effectively. More importantly, the ML model identified positions on the π‐spacer (*meso*‐aryl on the Zn‐porphyrin macrocycle) rather than on the donor and acceptor as the most critical features.

**Figure 9:**
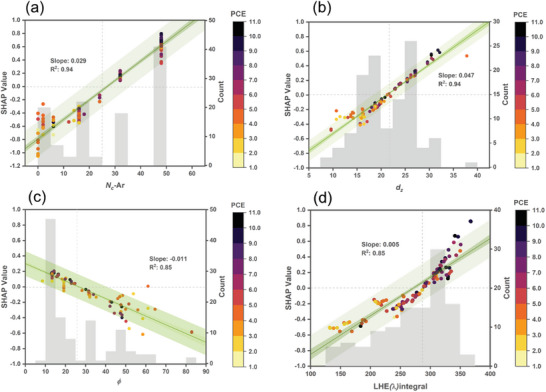
Molecular information of Zn‐porphyrin‐based dye sensitizers extracted from the optimized CNN/MDS‐GS+ABS+ET model. Distribution plots correlating the a) *N_C_*‐Ar, b) title angle (*ϕ* ), and c) vertical distance (*d_z_*), and LHE(λ)_integral_ values with their respective SHAP values based on the optimized CNN/MDS−GS+ABS+ET model. The figure displays linear fitting results, including the slope and *R^2^* value. The dark‐colored narrow band represents the 95% confidence interval, while the light‐colored wide band indicates the 95% prediction interval. The histogram represents the frequency distribution of dye counts.

In the analysis of the SHAP plot pertaining to *N_C_*‐Ar as depicted in **Figure** **9**a, a discernible positive linear correlation is evident between this particular MD and its corresponding SHAP value. The linear regression model reveals a slope of 0.029, emphasizing the direction of this correlation. Conversely, when examining the analogous plots for *N_C_*‐D and *N_C_*‐A (number of carbon atoms of alkyl chain attached on donor and acceptor, respectively, as represented in Figure S2, Supporting Information), contrasting trends emerge, characterized by nearly zero and negative slopes (0.007 and −0.037, respectively). These observations underscore the presence of no correlation between the *N_C_*‐D and its SHAP value and negative correlations between the *N_C_*‐A and its SHAP value. The relationship between the number of carbon atoms of alkyl chains of dyes and their conversion efficiency in DSSCs has been examined experimentally. Arakawa and colleagues^[^ ^48^ ^]^ conducted a study involving a series of benzothiazole merocyanines featuring varying alkyl chain lengths attached to the benzothiazole ring. Their research findings^[^ ^49^ ^]^ revealed a positive correlation between the number of carbon atoms of the alkyl side chain attached to the benzothiazole ring and both the conversion efficiency and the incident photon‐to‐current efficiency (IPCE) value. Notably, they observed that as the alkyl side chain length increased, so did the conversion efficiency and the IPCE value. The dye possessing the longer linear alkyl chain, consisting of 18 carbon atoms, emerged as the most efficient sensitizer in terms of PCE.

In contrast to *N_C_*‐Ar, the number of carbon atoms of the alkyl chain attached to the donor (*N_C_*‐D) or acceptor (*N_C_*‐A) demonstrates no and negative correlation with their respective SHAP values. This suggests that alkyl chains attached to two *meso*‐aryls can effectively mitigate dye aggregation and reduce charge recombination on the TiO_2_ surface, compared to the *N_C_*‐D and *N_C_*‐A configurations. It is established that porphyrin macrocycles tend to aggregate, and as such, the alkyl chain attached to the two *meso*‐aryl groups can efficiently envelop the porphyrin ring, thereby reducing the extent of dye aggregation. In recent notable studies, Yeh and colleagues^[^ ^7^, ^50^ ^]^ demonstrated the effectiveness of “double fence” porphyrin dyes, featuring eight long alkoxyl chains attached to the four β‐phenyl groups. These dyes exhibited higher PCE compared to the “single fence” *meso*‐substituted counterparts. These results suggest that increasing the number of “fences” on the porphyrin macrocycle can enhance cell performance. This finding underscores the crucial role of alkyl chains on the *meso*‐aryl positions of the porphyrin macrocycle in reducing molecular aggregation and charge recombination at the TiO_2_/dye/electrolyte interface. Our findings underscore the critical importance of strategically positioning alkyl chains on the *meso*‐aryl positions of the porphyrin macrocycle and support the utilization of the double fence model to maximize the performance of DSSCs.

#### 3.4.2 Tilt Angle of Dye Adsorbed on TiO_2_ Influences Dye Loading

The tilt angle of dye adsorption on TiO_2_ influences molecular footprint and dye packing on TiO_2_ surface. Figure 9b displays the distribution plot of tilt angles and their respective SHAP values. The plot indicates an inverse relationship, where smaller tilt angles of dyes are associated with positive SHAP values, contributing positively to the predicted PCE values. The tilt angle of a dye influences its occupancy on the TiO_2_ surface, with a smaller tilt angle corresponding to a higher dye‐loading capacity and vice versa. The dye‐loading capacity plays a crucial role in light‐harvesting efficiency. Marks, Chen, and their coworkers^[^ ^34^ ^]^ investigated the correlation between dye‐loading and the tilt angles of dyes concerning the TiO_2_ surface, with a specific focus on a series of four D‐π‐bridge‐A metal‐free tetrathienoacene (TTAR) sensitizers. These sensitizers all feature the same cyanoacrylic acid anchoring group (A). Notably, they examined two variations within this series: TPA‐TTAR‐A and TPA‐T‐TTAR‐A, which demonstrated relatively low dye loadings of 2.1 × 10^−8^ and 1.3 × 10^−8^ mol mg^−1^, respectively. It is important to note that “TPA” and “T” represent the triphenylamine and thiophene moieties, respectively. In contrast, TPA‐TTAR‐T‐A and TPA‐T‐TTAR‐T‐A exhibited significantly higher dye loadings, measured at 7.4 × 10^−8^ and 6.6 × 10^−8^ mol mg^−1^, respectively. The dye‐loading capacities of these four dyes align with their corresponding tilt angles, which revealed larger tilt angles for TPA‐TTAR‐A (≈70°) and TPA‐T‐TTAR‐A (≈74°) and smaller tilt angles for TPA‐TTAR‐T‐A (≈48°) and TPA‐T‐TTAR‐T‐A (≈58°), as confirmed through X‐ray reflectivity (XRR) measurements. The dye‐loading is intricately sensitive to these tilt angles, and notably, TPA‐TTAR‐T‐A exhibited the highest PCE of 10.1%.

#### 3.4.3 Vertical Distance of Dye's Donor to TiO_2_ Surface Affects the Charge Recombination Rate

The vertical distance of the dye's donor to the TiO_2_ surface is influenced not only by the tilt angle of the dye but also by the molecular length of the dye. Figure 8c depicts a distribution plot correlating the vertical distance (*d_z_*) values with their respective SHAP values. The plot illustrates a positive relationship, where larger *d_z_* values in dyes are associated with positive SHAP values, thereby making a positive contribution to the predicted PCE values. It is important to note that the *d_z_* value in dyes significantly influences their charge recombination rate on the TiO_2_ surface, with smaller *d_z_* values corresponding to higher charge recombination rates, and vice versa. In the study conducted by Marks, Chen, and their colleagues,^[^ ^34^ ^]^ specifically focusing on TPA‐TTAR‐A and TPA‐T‐TTAR‐A, as well as TPA‐TTAR‐T‐A and TPA‐T‐TTAR‐T‐A, they performed an analysis of the open‐circuit voltage decay (OCVD) dynamics.^[^ ^34^ ^]^ This analysis revealed varying decay rates among these sensitizers, shedding light on the intricacies of charge recombination processes. Specifically, the TPA‐TTAR‐T‐A‐based solar cell exhibited the slowest decay dynamics, while the dye TPA‐TTAR‐A displayed the most rapid decay. These observations suggest the lowest recombination rate for TPA‐TTAR‐T‐A‐based DSSCs. This phenomenon can be attributed to the vertical distance between the donor (hole) on the dye and the TiO_2_ surface. Dyes adopting a more vertical orientation are characterized by a greater distance, leading to slower charge recombination rates. Conversely, dyes with larger tilt angles tend to exhibit faster charge recombination rates.

**Figure 10:**
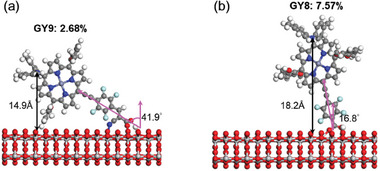
Adsorption configurations of a) **GY9** and b) **GY8** on the surface of anatase TiO₂. The alkyl chains are replaced by methyl groups for clarity. The color scale represents the relative vertical distance of the atoms in the molecules from the TiO₂ surface.

The tilt angle of dye adsorption on TiO₂ is influenced by the binding configuration of its binding group. For example, the cyanoacrylate group can bind to the TiO₂ surface in a tridentate mode, whereas the carboxylate group binds in a bidentate mode. **GY9**, which binds to the TiO₂ surface via the cyanoacrylate group in a tridentate mode, exhibits a tilt angle of 41.9°, resulting in a *d_z_* of 14.2 Å [**Figure** **10**a]. In contrast, **GY8**, which binds in a bidentate mode via the carboxylate group, has a tilt angle of 16.8°, leading to a *d_z_* of 18.2 Å [Figure 10b]. Therefore, **GY8** might have a higher dye loading and a slower charge recombination rate compared to **GY9**.These results are consistent with experimental findings, indicating that **GY9** has a lower PCE of 2.68%, while **GY8** achieves a higher PCE of 7.57%.

#### 3.4.4 Light Harvesting Efficiency

LHE quantifies the effectiveness of a photosensitive material in capturing and converting incident light into electrical current. In Figure 9d, a distribution plot depicts the relationship between LHE(λ)_integral_ values and their corresponding SHAP values. The plot reveals a proportional relationship, where larger LHE(λ)_integral_ values are associated with positive SHAP values, contributing positively to the predicted PCE values. Enhancing LHE is of paramount importance as it directly influences the PCE of DSSCs.

Our study emphasizes the critical importance of four key MDs—namely, *N_C_*‐Ar, *ϕ* , *d_z_*, and LHE(λ)_integral_ values—in predicting PCE values in DSSCs through ML models. Our ML model's results exhibit a strong alignment with experimental observations, underscoring the pivotal role these MDs play in both predicting and understanding the working mechanism in DSSCs. These findings are particularly valuable as they allow for the establishment of associated structure–function relationships, effectively providing a set of chemical rules. This “toolkit” can be leveraged for the rational design and molecular engineering of more suitable DSSC dyes, thereby advancing the field of DSSC technology.

### 3.5 Model Validation by Predicting Performance of Uncharted Novel Zn‐porphyrin DSSCs

The true strength of our ML approach is demonstrated in the “blind test.” To evaluate the predictive capabilities of our ML models, we designed and synthesized 17 novel Zn‐porphyrin molecules (Figure 2) that were not previously documented in the literature or included in our training dataset. Their experimentally measured PCE values range from 2.68% to 10.28%, with their photovoltaic parameters provided in the Table S5, Supporting Information. In Figure 2a, we present 9 Zn‐porphyrin molecules based on the **YD2‐o‐C8** framework,^[^ ^3^ ^]^ featuring variations in their donor components. Figure 2b showcases 2 Zn‐porphyrin molecules derived from the **YD24** framework,^[^ ^51^ ^]^ where modifications were made to the donor groups. Additionally, Figure 2c displays 6 Zn‐porphyrin molecules originating from the **YD24** framework,^[^ ^51^ ^]^ with variations in their acceptor moieties. Notably, with the exception of the acceptor in **GY60** and the donor in **GY33**, all the donors and acceptors depicted in Figure 2 were completely absent from our training dataset. This experimental assessment serves as a rigorous “blind test.” of our ML models' predictive accuracy in an uncharted chemical space.

**Figure 11:**
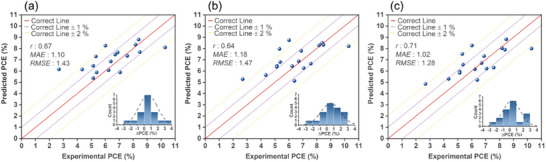
Performance assessment and predicted PCEs of 17 newly designed Zn‐porphyrin‐sensitized solar cells (as displayed in Figure 2) compared to their experimentally measured values using various ML Models, including a) ANN/MDS−GS, b) ANN/MDS−GS+ABS, and c) ANN/MDS−GS+ABS+ET. The inset figure displays the distribution of prediction errors (ΔPCE) calculated as the predicted PCE minus the experimental PCE.

**Figure** **11** presents a comparative analysis between the predicted PCE values generated by ANN learner, utilizing three different MD Sets (MDS−GS, MDS−GS+ABS, and MDS−GS+ABS+ET), trained on our dataset. The predictions are compared with the experimentally measured PCEs of DSSCs that employ 17 newly designed Zn‐porphyrin molecules, listed in Figure 2. Predicted PCE values generated by LGBM and CNN learners in conjunction with the mentioned MD Sets can be found in Figure S3, Supporting Information. For specific numerical values of predicted PCEs, please refer to Table S6, Supporting Information. Based on three metrics (*r*, *MAE*, and *RMSE*), ANN learner outperforms than LGBM and CNN learners for any of the thee MD Sets used. When employing MDS−GS, ANN achieves an *r*‐value of 0.67, *MAE* of 1.10, and *RMSE* of 1.43, while CNN achieves an *r*‐value of 0.65, *MAE* of 1.10, and *RMSE* of 1.46. For the ANN/MDS−GS model, an analysis of prediction errors reveals that more than 10 of the 17 newly designed dyes exhibit absolute prediction errors within a 1% margin, with 4 of the dyes falling within the range of 1–2% prediction error. The highest prediction error is observed for **GY9**, with a 3.48% overprediction. Concerning the predictive performance of ANN/MDS−GS and CNN/MDS−GS models for these 17 newly designed Zn‐porphyrin‐sensitized solar cells, it is suggested to employ the faster‐calculated MDS−GS for the initial‐stage virtual screening of unexplored Zn‐porphyrins with novel donor and acceptor groups.

In utilizing MDS−GS+ABS, ANN exhibits slightly poorer performance than when using MDS−GS. In the ANN/MDS−GS+ABS model, an analysis of prediction errors reveals that more than 8 of the 17 newly designed dyes exhibit absolute prediction errors within a 1% margin, with 5 of the dyes falling within the range of 1–2% prediction error. The highest prediction error is observed for **GY48**, with a 2.73% overprediction.

The **GY9**, which is 3.48% overpredicted by ANN/ MDS−GS model, is remediated to 2.33% overpredicted by MDS−GS+ABS model. Given the predictive performance of ANN/MDS−GS+ABS model for these 17 newly designed Zn‐porphyrin‐sensitized solar cells, and considering the higher cost associated with TD‐DFT calculations, the ANN/MDS−GS model can be employed for the second‐stage virtual screening of candidate Zn‐porphyrins identified using the ANN/MDS−GS and/or CNN/MDS−GS models.

Specifically, the ANN/MDS−GS+ABS+ET model outperforms its counterparts using either MDS−GS or MDS−GS+ABS. For the ANN/MDS−GS+ABS+ET model, the distribution of prediction errors reveals that 10 of the 17 newly designed dyes exhibit absolute prediction errors within the 1% margin, in particular, 6 of them fall within the 0.5% margin. While 3 of the dyes fall within the range of 1–2% prediction error. None of the predictions have errors beyond 2.5%. The ANN/MDS−GS+ABS+ET model exhibits the highest prediction error for **GY32**, with a 2.28% underprediction, The **GY9** peptide, initially overpredicted by 3.48% using the ANN/MDS−GS model and 2.33% using the ANN/MDS−GS+ABS model, has its prediction error reduced to 2.17% with the ANN/MDS−GS+ABS+ET model. These results underscore the precision of the ANN/MDS−GS+ABS+ET model in capturing the performance characteristics of the investigated dyes. An evaluation of the ANN/MDS−GS+ABS+ET model's performance metrics underscores its reliability in this independent validation. The model achieves a *MAE* of 1.02% and an *RMAE* of 1.28%, which, while slightly larger than the corresponding values based on the testing set (1.00% and 1.23%, respectively), still demonstrate its robustness. The results presented here confirm the effectiveness of our ML models, especially the ANN/MDS−GS+ABS+ET model, in accurately predicting and navigating the complex landscape of novel Zn‐porphyrins for potential use in DSSCs.

It is worth noting that four of these 17 Zn‐porphyrin DSSCs (**GY32**, **GY33**, **GY34**, and **GY45**) exhibit experimental PCE values exceeding 8%. All these dyes are based on the **YD42** framework and vary in terms of the donor. Notably, the central symmetric nature of the acceptor benzoic acid results in the dyes binding more vertically to the TiO_2_ surface, with a smaller tilt angle and positioning the donor away from the TiO_2_ surface. As suggested by our ML models, these properties could contribute to the higher PCE values observed.

### 3.6 In Silico Virtual Screening of Potential Zn‐Porphyrins

One of the primary objectives of training the ML model is to apply it to a vast number of unknown candidate dyes and identify potential high‐performance ones. In the context of Zn‐porphyrins, the design typically involves incorporating an electron donor (D) at the *meso*‐position of the porphyrin and an electron acceptor (A) at the *meso*‐position opposite to the donor. This configuration facilitates efficient photo‐induced charge separation and transfer. Modifying the donor and acceptor moieties allows for tuning the molecular energy levels and the absorption band shift.

**Figure 12:**
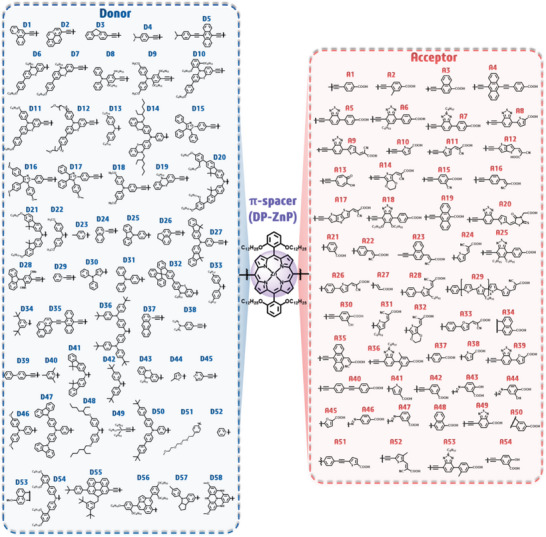
Chemical structures of donor (D), π‐spacer (DP‐ZnP), and acceptor (A) building components, along with their combined configurations.

To generate new candidate dyes, we utilized a set of 58 donors and 54 acceptors, as detailed in **Figure** **12**, sourced from our dataset and literatures.^[^ ^8^, ^52^, ^53^, ^54^, ^55^ ^]^ The core component of all these candidates remained consistent, featuring the Zn‐porphyrin core, specifically the 5,10‐di‐[1,3‐bis(dodecyloxy)‐phenyl] Zn‐porphyrin (denoted DP‐ZnP). Combining these 58 donors and 54 acceptors within D‐π(DP‐ZnP)‐A configurations resulted in a total of 3132 candidate molecules. After excluding dyes that had already been published, we were left with 3084 potential candidates.

For each of these molecules, we conducted ground‐state geometry optimizations using DFT to ensure their structural stability. Subsequently, these molecules underwent a screening process employing the ANN/MDS−GS and CNN/MDS−GS models to identify the most promising candidates. We note that the MDs, specifically the *ϕ* and *d_z_*_._, of these candidates are rapidly constructed solely based on their optimized geometry and the pre‐calculated configurations of the dye's anchor on TiO_2_. For a comprehensive list of predicted PCE values for all 3084 candidate molecules, please refer to a separate Excel file provided. Here, we present the top 10 high PCE sensitizers (**Table** **1**) as predicted by the ANN/MDS‐GS model, all demonstrating a predicted PCE exceeding 9.50%. Additionally, these 10 sensitizers are also predicted by the CNN/MDS‐GS model to have a PCE greater than 9.0%. Among these, **D58**, a nitrogen‐doped triphenylamine derivative suggested by previous theoretical calculations, is frequently present as their donor. It's worth noting that these high PCE sensitizers, featuring **D58** as their donors, display higher HOMO energy levels compared to others,^[^ ^55^ ^]^ as determined through DFT calculations. For example, the **D58‐DP‐ZnP‐A44** dye exhibits a HOMO energy of −3.91 eV, which is higher in energy than the average HOMO energy (−4.93 eV) of our database. Interestingly, some D‐π‐A organic dyes containing **D58** as their constituents were previously predicted to exhibit PCEs exceeding 10% by ML models.^[^ ^13^ ^]^

**Table 1:** Zn‐porphyrin sensitizers with top PCE values identified through a three‐stage virtual screening process, as predicted by the ANN/MDS‐GS, ANN/MDS‐GS+ABS, and ANN/MDS‐GS+ABS+ET models. In each stage, the top PCE values of Zn‐porphyrin sensitizers were cross‐validated using a CNN model with the corresponding MDS. For further details, please refer to the main text. The PCE values predicted by the ANN model are shown next to the molecules, with the CNN model's predicted PCE values indicated in parentheses.

	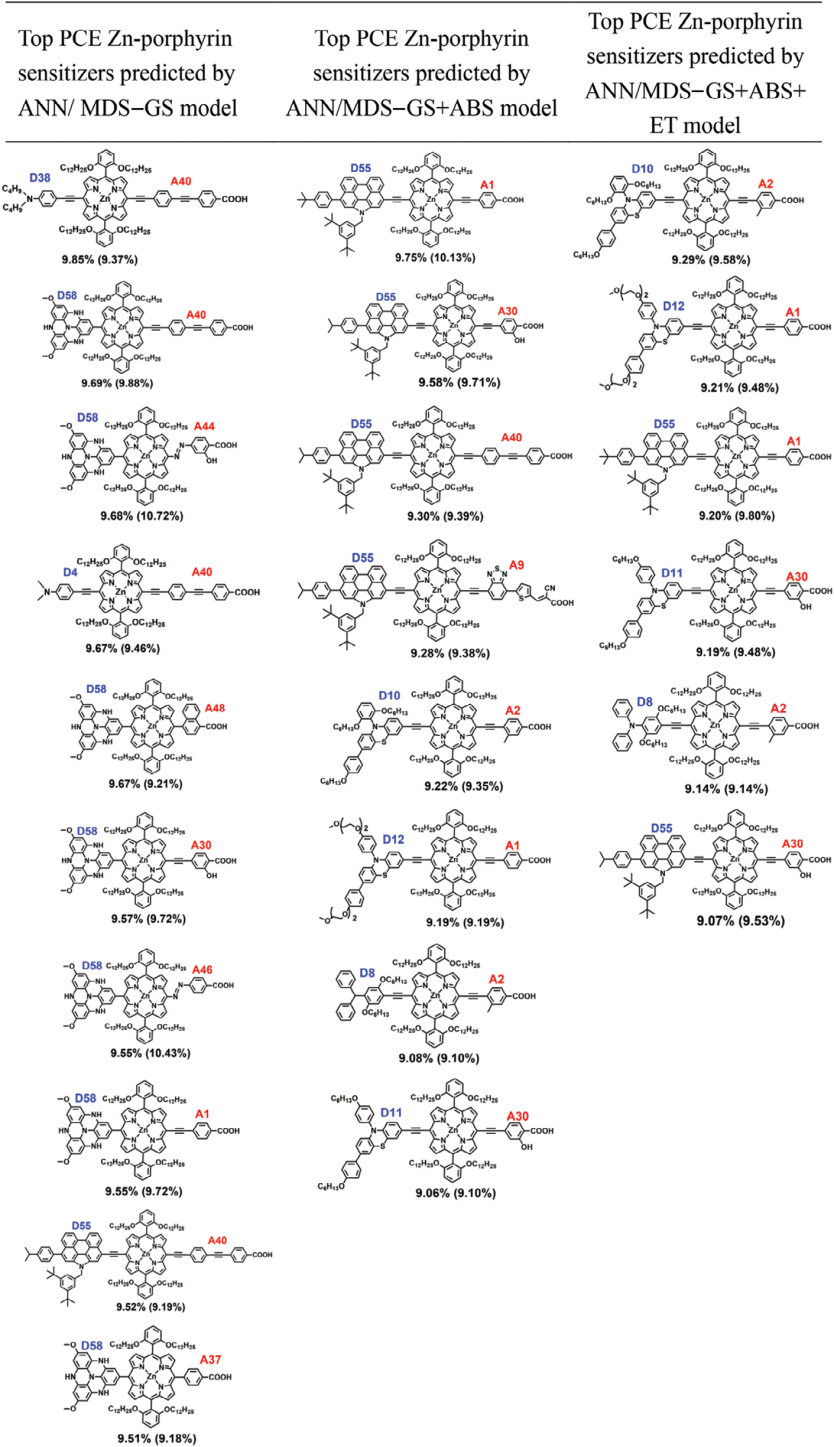

Based on the predicted PCEs obtained from the ANN/MDS−GS and CNN/MDS−GS predictive model, we have identified 39 high‐performance molecules with predicted PCEs exceeding 9% by both models. These molecules will undergo more precise screening using the ANN/MDS−GS+ABS and CNN/MDS−GS+ABS predictive model. We present the top 8 high PCE sensitizers (Table 1) as predicted by the ANN/MDS−GS+ABS model, all demonstrating a predicted PCE exceeding 9.00%. Additionally, these 8 sensitizers are also predicted by the CNN/MDS−GS+ABS model to have a PCE greater than 9.0%. Among these molecules, **D55** is frequently used as their donors. Remarkably, the highest‐ranked molecule (**D55‐DP‐ZnP‐A1**, predicted PCE = 9.75% by ANN/MDS−GS+ABS model) has not only been synthesized but also reported to exhibit high efficiency, boasting a PCE of 10.5% when used with Co(II/III)‐based electrolyte, as documented by Wu, Wang, and their collaborators.^[^ ^52^ ^]^ Although our predictive ML model was originally developed based on I^−^/I_3_^−^ electrolyte, it demonstrates the potential of employing **D55** as the electron donor in sensitizers. It's worth noting that the **D55** donor was not part of our training set. Nevertheless, our ANN/MDS−GS+ABS and CNN/MDS−GS+ABS predictive models successfully identify **D55‐DP‐ZnP‐A1** as the top molecule in our virtual screening. This underscores the accuracy, reliability, and generalization capability of the ANN/MDS−GS+ABS and CNN/MDS−GS+ABS predictive models.

However, it's worth noting that some molecules are predicted to have PCEs below 8% when assessed by the ANN/MDS−GS+ABS predictive model. Specifically, **D58‐**containing dyes, which were initially predicted to exhibit high PCEs by the ANN/MDS−GS and CNN/MDS−GS predictive model, are now predicted to have lower PCEs by the ANN/MDS−GS+ABS predictive model.

For instance, the **D58‐DP‐ZnP‐A44** molecule, initially ranked as the top PCE performer by the ANN/MDS−GS and CNN/MDS−GS predictive model, is now predicted to achieve a PCE below 8%. Our investigation has revealed that **D58**‐containing dyes typically exhibit smaller values for LHE(λ)_integral_ compared to others. For example, the top‐ranked molecule, **D58‐DP‐ZnP‐A44** has an LHE(λ)_integral_ value of 222.3, which is notably smaller than the average LHE(λ)_integral_ value (273.0) within our database. Additionally, we have observed that the ICT transition in **D58**‐containing molecules is relatively weak, resulting in smaller LHE(λ)_integral_ values. As the LHE(λ)_integral_ value has shown a positive correlation with the predicted PCE value in our ANN/MDS−GS+ABS model, it follows that the ANN/MDS‐GS+ABS model predicts **D58**‐containing dyes with lower PCEs.

Based on the predicted PCEs obtained from the ANN/MDS−GS+ABS and CNN/MDS−GS+ABS predictive model, we have identified 8 high‐performance molecules with predicted PCEs exceeding 9%. These molecules will undergo more computationally‐cost screening using the ANN/MDS−GS+ABS+ET and CNN/MDS−GS+ABS+ET predictive models. Out of the total 8 molecules evaluated, the ANN/MDS−GS+ABS+ET and CNN/MDS−GS+ABS+ET predictive model predicts that 6 of them (Table 1) are expected to demonstrate a PCE greater than 9.0%.

It is worth noting that all six of these dyes exhibit a *para*‐benzoic acid moiety as their acceptors, which, as discussed above, allows for more vertical binding on TiO_2_ and can lead to higher PCE values. Considering that our ANN/MDS−GS+ABS+ET and CNN/MDS−GS+ABS+ET predictive models typically underestimates the PCE of high‐performing sensitizers, it's possible that these candidate sensitizers predicted to have higher PCEs may still be underestimated.

## 4 Conclusions

In this study, we present accurate, predictive, and interpretable ML models that establish correlations between the molecular structural and electronic properties of 127 Zn‐porphyrin‐sensitized solar cells and their PCEs. These models were rigorously validated by predicting the PCE of 17 new Zn‐porphyrin‐based sensitizers intended for DSSCs. Our ML models successfully predicted the performance of 17 newly designed Zn‐porphyrin dyes, achieving a similar *MAE* as their training set counterparts, with 9 dyes predicted within a 1% error range. These results underscore the significance of our models in exploring uncharted chemical spaces of Zn‐porphyrins and their utility for rational design and further virtual screening.

In addition, we have developed and identified four highly significant MDs through the SHAP analysis: *N_C_*‐Ar, *ϕ* , *d_z_*, and LHE(λ)_integral_. These findings align with experimental observations and provide essential chemical insights into the microscopic photo‐electronic conversion processes in DSSCs. This information, encoded within easily evaluated PCE values, can be extracted with basic tools like paper, pencils, and a low‐cost computer, making it accessible to design candidate dyes and other photovoltaic materials.

The most significant advantage of ML in this context, and in other fields, is the substantial savings in time and resources. To make large‐scale virtual screening of numerous new Zn‐porphyrins feasible, which traditional experiments cannot achieve, we developed a three‐tiered system of ML models based on three distinct levels of MD Sets. While MDS calculations take days on a low‐cost computer, our ML models predict results in a fraction of a second, allowing newly designed sensitizers to be analyzed before synthesis and fabrication. This is crucial in both academic and industrial settings, where synthesizing sensitizers can take months. This approach enables researchers to evaluate promising candidates early. Additionally, more experimental data could enhance the reliability of our ML models, improving the accuracy of PCE predictions for DSSCs. This will be crucial for optimizing ML use in this field.

The strategies and findings presented in this study have the potential to make a substantial contribution to the broader fields of photovoltaic research. The methodologies developed here can be effectively extended to other photovoltaic systems, including perovskite solar cells, organic photovoltaics, and hybrid systems, offering significant opportunities for further exploration. Integrating these approaches with experimental workflows will enhance the efficiency of material discovery and enable the exploration of new chemical spaces. Moreover, the implementation of automation and high‐throughput screening systems, which combine ML predictions with robotic synthesis, holds the promise of revolutionizing the rapid identification of promising candidates for renewable energy applications. These advancements could play a crucial role in driving the development of the next generation of photovoltaic materials.

## Conflict of Interest

The authors declare no conflict of interest.

## Supporting information



Supporting Information

Supporting Information

Supporting Information

## Data Availability

The data that support the findings of this study are available in the supplementary material of this article.
