# Supplementary material for: Advanced High‐Throughput Rational Design of Porphyrin‐Sensitized Solar Cells Using Interpretable Machine Learning
Source: Adv Sci (Weinh). 2024 Sep 24;11(43):2407235. doi: 10.1002/advs.202407235 (PMC11578383; doi:10.1002/advs.202407235)
Supplement: Supplementary file 1 — Supporting Information [file ADVS-11-2407235-s001.docx]

**Supporting Information**

Advanced High-Throughput Rational Design of Porphyrin-Sensitized Solar Cells Using Interpretable Machine Learning

Jian-Ming Liao,1 Yu-Hsuan Chen,2 Hsuan-Wei Lee,2 Bo-Cheng Guo,2 Po-Cheng Su,2 Lun-Hong Wang,2 Nagannagari Masi Reddy,2 Aswani Yella,3 Zhao-Jie Zhang,1 Chuan-Yung Chang,1 Chia-Yuan Chen,1,4 Shaik M Zakeeruddin,3,* Hui-Hsu Gavin Tsai,1,4,* Chen-Yu Yeh,2,* and Michael Grätzel3,*

1Department of Chemistry, National Central University, No. 300, Zhongda Rd., Zhongli District, Taoyuan City, 32001, Taiwan

2Department of Chemistry, i-Center for Advanced Science and Technology (i-CAST), Innovation and Development Center of Sustainable Agriculture (IDCSA), National Chung Hsing University, Taichung City 402, Taiwan.

3Laboratory for Photonics and Interfaces, Institute of Chemical Sciences and Engineering, É cole Polytechnique Fédérale de Lausanne, Lausanne-1015, Switzerland

4Research Center of New Generation Light Driven Photovoltaic Modules National Central University, Taoyuan 32001,Taiwan

*To whom correspondence should be addressed.

E-mail: hhtsai@cc.ncu.edu.tw (H.-H. G. T.); cyyeh@dragon.nchu.edu.tw (C.-Y.Y.); shaik.zakeer@epfl.ch (S.M.Z.); and michael.graetzel@epfl.ch (M.G.)

Table S1. Chemical structures and photovoltaic parameters (short-circuit current density (*J_sc_*), open-circuit voltage (*V_oc_*), fill factor (*FF*) and power conversion efficiency (PCE) measured under AM 1.5G sunlight) of 127 dyes in database

| No. | Chemical Structure &  Name | *J_sc_*  [mA/cm^2^] | *V_oc_* [V] | *FF* | PCE  [%] |
| --- | --- | --- | --- | --- | --- |
| **1**^[1]^ |  | 10.3 | 0.72 | 0.69 | 5.11 |
| **2**^[2]^ |  | 16.84 | 0.67 | 0.7 | 8.02 |
| **3**^[3]^ |  | 10.56 | 0.66 | 0.73 | 5.11 |
| **4**^[3]^ |  | 15.92 | 0.682 | 0.72 | 7.83 |
| **5**^[3]^ |  | 14.04 | 0.654 | 0.73 | 6.62 |
| **6**^[3]^ |  | 17.02 | 0.678 | 0.72 | 8.26 |
| **7**^[3]^ |  | 19.63 | 0.711 | 0.72 | 10.06 |
| **8**^[4]^ |  | 12.92 | 0.682 | 0.72 | 6.3 |
| **9**^[4]^ |  | 17.26 | 0.689 | 0.68 | 8 |
| **10**^[4]^ |  | 15.51 | 0.685 | 0.7 | 7.4 |
| **11**^[5]^ |  | 14.01 | 0.716 | 0.68 | 6.79 |
| **12^[5]^** |  | 14.23 | 0.717 | 0.68 | 6.91 |
| **13**^[5]^ |  | 4.12 | 0.63 | 0.72 | 1.86 |
| **14**^[6]^ |  | 20 | 0.7 | 0.74 | 10.41 |
| **15**^[7]^ |  | 17.77 | 0.73 | 0.75 | 9.73 |
| **16**^[7]^ |  | 14.17 | 0.68 | 0.72 | 6.97 |
| **17**^[7]^ |  | 17.76 | 0.72 | 0.74 | 9.51 |
| **18**^[7]^ |  | 14.2 | 0.68 | 0.69 | 6.7 |
| **19**^[8]^ |  | 15.7 | 0.648 | 0.7 | 7.12 |
| **20**^[8]^ |  | 15.36 | 0.685 | 0.7 | 7.37 |
| **21**^[8]^ |  | 16.35 | 0.657 | 0.71 | 7.63 |
| **22**^[8]^ |  | 17.65 | 0.75 | 0.72 | 9.53 |
| **23**^[9]^ |  | 17.14 | 0.737 | 0.72 | 9.21 |
| **24**^[6]^ |  | 7.69 | 0.65 | 0.75 | 3.84 |
| **25**^[10]^ |  | 19.17 | 0.736 | 0.72 | 10.17 |
| **26**^[6]^ |  | 5.81 | 0.58 | 0.76 | 2.55 |
| **27**^[11]^ |  | 6.7 | 0.61 | 0.68 | 2.8 |
| **28**^[11]^ |  | 10.6 | 0.62 | 0.62 | 4.1 |
| **29**^[12]^ |  | 0.88 | 0.51 | 0.67 | 0.3 |
| **30**^[13]^ |  | 3.6 | 0.53 | 0.58 | 1.1 |
| **31**^[14]^ |  | 8.1 | 0.606 | 0.774 | 3.6 |
| **32**^[15]^ |  | 7.2 | 0.67 | 0.67 | 3.1 |
| **33**^[14]^ |  | 8.9 | 0.66 | 0.71 | 4.5 |
| **34**^[10]^ |  | 18.44 | 0.69 | 0.73 | 9.34 |
| **35**^[16]^ |  | 5.79 | 0.617 | 0.67 | 2.40 |
| **36**^[16]^ |  | 13.6 | 0.701 | 0.63 | 6 |
| **37**^[16]^ |  | 13.68 | 0.711 | 0.7 | 6.76 |
| **38**^[17]^ |  | 16.42 | 0.755 | 0.67 | 8.32 |
| **39**^[17]^ |  | 16.9 | 0.755 | 0.65 | 8.28 |
| **40**^[17]^ |  | 13.36 | 0.716 | 0.68 | 6.46 |
| **41^[17]^** |  | 15.66 | 0.723 | 0.66 | 7.5 |
| **42**^[16]^ |  | 10.85 | 0.713 | 0.69 | 5.34 |
| **43**^[16]^ |  | 5.05 | 0.651 | 0.64 | 5.65 |
| **44**^[16]^ |  | 10.05 | 0.65 | 0.67 | 4.38 |
| **45**^[18]^ |  | 10.7 | 0.67 | 0.7 | 5.01 |
| **46**^[19]^ |  | 17.07 | 0.725 | 0.7 | 8.6 |
| **47**^[19]^ |  | 18.02 | 0.72 | 0.67 | 8.7 |
| **48**^[19]^ |  | 18.79 | 0.7 | 0.72 | 9.5 |
| **49**^[20]^ |  | 12.1 | 0.701 | 0.68 | 5.76 |
| **50**^[21]^ |  | 12.33 | 0.8 | 0.64 | 6.27 |
| **51**^[21]^ |  | 14.44 | 0.772 | 0.63 | 7 |
| **52**^[22]^ |  | 16.27 | 0.728 | 0.7 | 8.2 |
| **53**^[22]^ |  | 17.9 | 0.711 | 0.68 | 8.6 |
| **54**^[23]^ |  | 18.04 | 0.742 | 0.68 | 9.05 |
| **55**^[23]^ |  | 18.4 | 0.731 | 0.7 | 9.51 |
| **56**^[23]^ |  | 19.36 | 0.741 | 0.7 | 10 |
| **57**^[23]^ |  | 18.53 | 0.752 | 0.7 | 9.72 |
| **58**^[23]^ |  | 19.47 | 0.75 | 0.71 | 10.32 |
| **59**^[23]^ |  | 18.36 | 0.74 | 0.69 | 9.61 |
| **60**^[24]^ |  | 12.95 | 0.6 | 0.66 | 5.14 |
| **61**^[24]^ |  | 10.96 | 0.61 | 0.68 | 4.55 |
| **62**^[25]^ |  | 12.76 | 0.58 | 0.59 | 4.38 |
| **63**^[25]^ |  | 13.61 | 0.6 | 0.62 | 5 |
| **64**^[26]^ |  | 10.98 | 0.62 | 0.49 | 3.34 |
| **65**^[27]^ |  | 18.38 | 0.778 | 0.675 | 9.65 |
| **66**^[28]^ |  | 10.51 | 0.7 | 0.72 | 5.19 |
| **67**^[28]^ |  | 12.79 | 0.701 | 0.72 | 6.42 |
| **68**^[28]^ |  | 17.93 | 0.711 | 0.72 | 9.12 |
| **69**^[29]^ |  | 17 | 0.712 | 0.69 | 8.39 |
| **70**^[29]^ |  | 13.28 | 0.717 | 0.7 | 6.61 |
| **71**^[29]^ |  | 7.43 | 0.704 | 0.69 | 3.62 |
| **72**^[30]^ |  | 8.42 | 0.65 | 0.82 | 4.17 |
| **73**^[30]^ |  | 6.67 | 0.59 | 0.79 | 3.13 |
| **74**^[31]^ |  | 10.65 | 0.648 | 0.69 | 4.77 |
| **75**^[31]^ |  | 12.19 | 0.689 | 0.7 | 5.92 |
| **76**^[31]^ |  | 13.1 | 0.61 | 0.72 | 5.75 |
| **77**^[31]^ |  | 15.72 | 0.7 | 0.69 | 7.59 |
| **78**^[32]^ |  | 15.6 | 0.73 | 0.68 | 7.7 |
| **79**^[33]^ |  | 14.99 | 0.716 | 0.66 | 7.13 |
| **80**^[33]^ |  | 15.73 | 0.68 | 0.64 | 6.84 |
| **81**^[33]^ |  | 15.6 | 0.694 | 0.68 | 7.32 |
| **82**^[22]^ |  | 18.83 | 0.645 | 0.64 | 7.94 |
| **83**^[34]^ |  | 15.78 | 0.699 | 0.68 | 7.4 |
| **84**^[30]^ |  | 4.82 | 0.6 | 0.77 | 2.42 |
| **85**^[30]^ |  | 5.19 | 0.61 | 0.78 | 2.45 |
| **86**^[35]^ |  | 6.55 | 0.585 | 0.67 | 2.59 |
| **87**^[24]^ |  | 10.96 | 0.61 | 0.68 | 4.55 |
| **88**^[36]^ |  | 4.24 | 0.6 | 0.72 | 1.82 |
| **89**^[37]^ |  | 21.43 | 0.689 | 0.713 | 10.51 |
| **90**^[37]^ |  | 20.05 | 0.685 | 0.702 | 9.64 |
| **91**^[37]^ |  | 12.9 | 0.65 | 0.691 | 5.79 |
| **92**^[37]^ |  | 21 | 0.68 | 0.691 | 9.87 |
| **93**^[37]^ |  | 13.6 | 0.64 | 0.682 | 5.94 |
| **94**^[38]^ |  | 14.18 | 0.59 | 0.68 | 5.71 |
| **95**^[38]^ |  | 11.37 | 0.57 | 0.7 | 4.54 |
| **96**^[38]^ |  | 11.56 | 0.56 | 0.7 | 4.54 |
| **97**^[15]^ |  | 5.8 | 0.62 | 0.65 | 2.3 |
| **98**^[15]^ |  | 4.3 | 0.62 | 0.67 | 2 |
| **99**^[18]^ |  | 10.9 | 0.64 | 0.68 | 4.7 |
| **100**^[39]^ |  | 7.5 | 0.66 | 0.69 | 3.5 |
| **101**^[40]^ |  | 10 | 0.579 | 0.75 | 4.37 |
| **102**^[40]^ |  | 7.31 | 0.558 | 0.77 | 3.16 |
| **103**^[41]^ |  | 4.9 | 0.546 | 0.73 | 2 |
| **104**^[41]^ |  | 11.8 | 0.617 | 0.65 | 4.7 |
| **105**^[41]^ |  | 9.6 | 0.58 | 0.69 | 3.6 |
| **106**^[42]^ |  | 10.9 | 0.629 | 0.71 | 4.88 |
| **107**^[42]^ |  | 10.4 | 0.602 | 0.73 | 4.53 |
| **108**^[16]^ |  | 10.81 | 0.708 | 0.67 | 5.13 |
| **109**^[4]^ |  | 11.78 | 0.644 | 0.71 | 5.4 |
| **110**^[4]^ |  | 9.07 | 0.624 | 0.73 | 4.1 |
| **111^[43]^** |  | 15.8 | 0.875 | 0.77 | 9.4 |
| **112**^[44]^ |  | 17.43 | 0.676 | 0.69 | 8.1 |
| **113**^[44]^ |  | 12.05 | 0.631 | 0.72 | 5.5 |
| **114**^[44]^ |  | 14.87 | 0.634 | 0.7 | 6.6 |
| **115**^[27]^ |  | 15.1 | 0.793 | 0.686 | 8.25 |
| **116**^[45]^ |  | 8.79 | 0.651 | 0.75 | 4.28 |
| **117**^[27]^ |  | 12.99 | 0.778 | 0.694 | 7.01 |
| **118**^[45]^ |  | 6.03 | 0.632 | 0.75 | 2.86 |
| **119**^[45]^ |  | 15.37 | 0.745 | 0.7 | 8.04 |
| **120**^[45]^ |  | 11.3 | 0.696 | 0.74 | 5.8 |
| **121**^[45]^ |  | 11.59 | 0.72 | 0.72 | 6.03 |
| **122**^[45]^ |  | 7.17 | 0.673 | 0.76 | 3.64 |
| **123**^[46]^ |  | 18.45 | 0.732 | 0.66 | 8.9 |
| **124**^[47]^ |  | 9.42 | 0.623 | 0.71 | 4.2 |
| **125**^[47]^ |  | 14.27 | 0.712 | 0.67 | 6.8 |
| **126**^[47]^ |  | 12.21 | 0.704 | 0.64 | 5.5 |
| **127**^[47]^ |  | 13.99 | 0.722 | 0.69 | 7 |


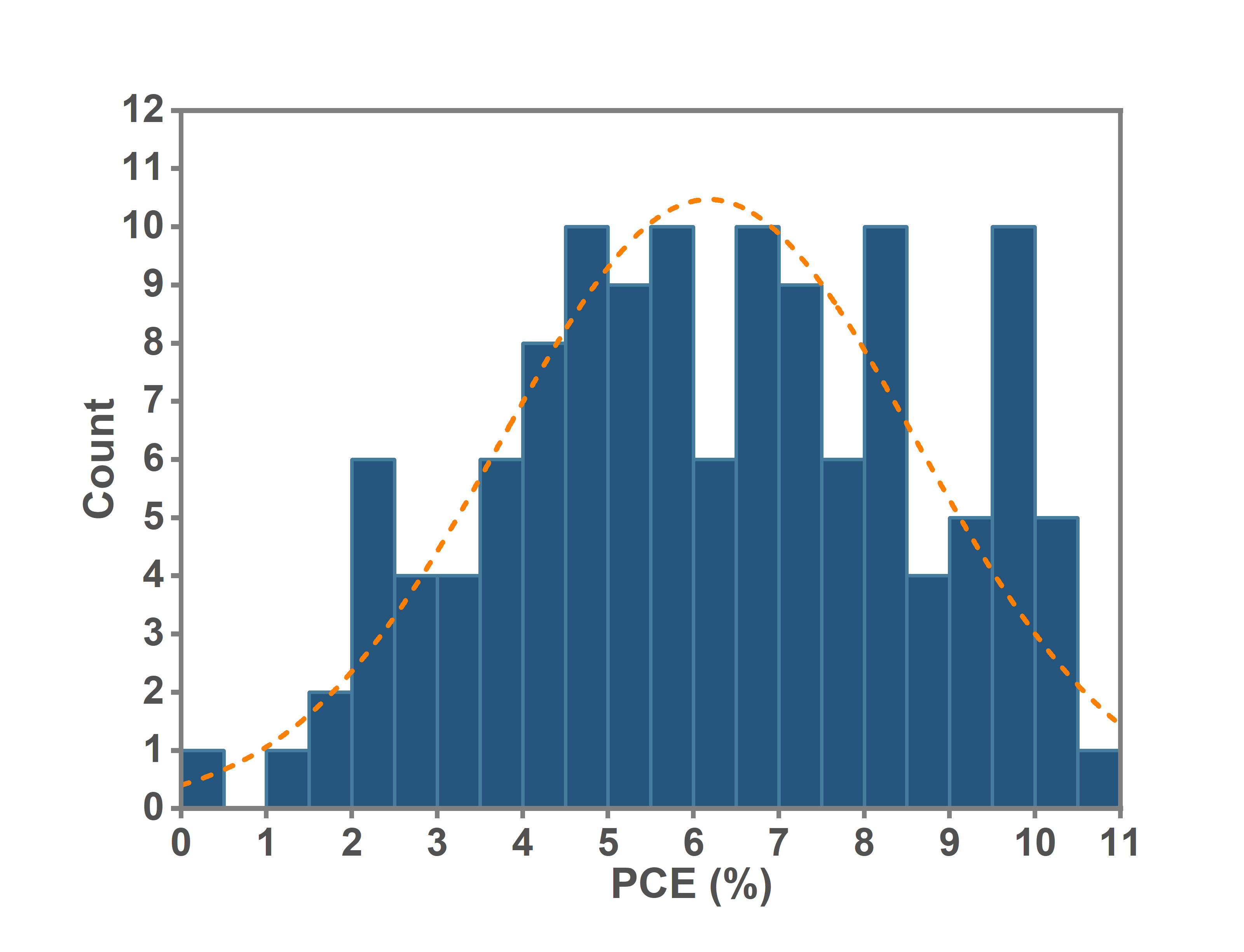


Figure S1. PCE Distribution of utilized Zn Porphyrin-Sensitized Solar cells.

Table S2. Symbols and physical meanings of 23 molecular descriptors (MD) assembled in the MDS−GS.

| No. | Symbol | Property |
| --- | --- | --- |
| 1 | *E*_H−2_ | Energy of HOMO−2 |
| 2 | *E*_H−1_ | Energy of HOMO−1 |
| 3 | E_H_ | Energy of HOMO |
| 4 | E_L_ | Energy of LUMO |
| 5 | E_L+1_ | Energy of LUMO+1 |
| 6 | E_L+2_ | Energy of LUMO+2 |
| 7 | ΔE_L-H−2_ | Energy difference between LUMO and HOMO−2 |
| 8 | ΔE_L+1-H−2_ | Energy difference between LUMO+1 and HOMO−2 |
| 9 | ΔE_L+2-H−2_ | Energy difference between LUMO+2 and HOMO−2 |
| 10 | ΔE_L-H−1_ | Energy difference between LUMO and HOMO−1 |
| 11 | ΔE_L+1-H−1_ | Energy difference between LUMO+1 and HOMO−1 |
| 12 | ΔE_L+2-H−1_ | Energy difference between LUMO+2 and HOMO−1 |
| 13 | ΔE_L-H_ | Energy difference between LUMO and HOMO |
| 14 | ΔE_L+1-H_ | Energy difference between LUMO+1 and HOMO |
| 15 | ΔE_L+2-H_ | Energy difference between LUMO+2 and HOMO |
| 16 | DM | Dipole moment |
| 17 | DU | Degree of Unsaturation^[48]^:  DU=1+1/2∑*n_i_*(*v_i_* −2), where *n_i_* is the number of atoms with valence *v_i_*. |
| 18 | DOF_core_ | Degree of freedom (alkyl chains were substituted with methyl) |
| 19 | *N*_C_-D | Number of alkyl chain’s carbon atoms attached to donor group |
| 20 | *N*_C_-Ar | Number of alkyl chain’s carbon atoms attached to aryl group |
| 21 | *N*_C_-A | Number of alkyl chain’s carbon atoms attached to acceptor group |
| 22 | *d_z_* | Vertical distance of the donor center of dye adsorbed on TiO_2_ to TiO_2_ surface. See the main text for details |
| 23 | *φ* | Tilt angle of dye adsorbed on TiO_2_ with respect to the normal of TiO_2_ surface. See the main text for details |

Table S3. Symbols and physical meanings of 19 molecular descriptors (MD) assembled in the MDS−ABS.

| No. | Symbol | Property |
| --- | --- | --- |
| 24 | λ_ICT_ | Maximum absorption wavelength of intramolecular charge transfer (ICT)  (λ > 600 nm) |
| 25 | *f*-λ_ICT_ | Oscillator strength of λ_ICT_ |
| 26 | λ_Q_ | Maximum absorption wavelength at Q band  (500 nm < λ < 600 nm) |
| 27 | *f*-λ_Q_ | Oscillator strength of λ_Q_ |
| 28 | λ_B_ | Maximum absorption wavelength at B band (λ < 500 nm) |
| 29 | *f*-λ_B_ | Oscillator strength of λ_B_ |
| 30 | LHE(λ_ICT_) | Light harvesting efficiency of λ_ICT_ |
| 31 | LHE(λ_Q_) | Light harvesting efficiency of λ_Q_ |
| 32 | LHE(λ_B_) | Light harvesting efficiency of λ_B_ |
| 33 | LHE(λ)_integral_ | Integral of Light harvesting efficiency at λ = 280-1200 nm |
| 34 | *J_sc_cal_* | Calculated maximum *J*_sc_ at λ = 280-1200 nm |
| 35 | CT-λ_ICT_ | Electron Density Difference Map (EDDM):  Charge transfer to the carboxylic acid group [and cyano (CN) group] on the anchoring group at λ_ICT_ |
| 36 | CT-λ_Q_ | EDDM :  Charge transfer to the carboxylic acid group [and cyano (CN) group] the anchoring group at λ_Q_ |
| 37 | CT-λ_B_ | EDDM :  Charge transfer to the carboxylic acid group [and cyano (CN) group] the anchoring group at λ_B_ |
| 38 | t_es_-λ_ICT_ | Singlet excited state lifetime of λ_ICT_ |
| 39 | t_es_-λ_Q_ | Singlet excited state lifetime of λ_Q_ |
| 40 | t_es_-λ_B_ | Singlet excited state lifetime of λ_B_ |
| 41 | ΔG_inj_ | Free energy of electron injection |
| 42 | E_b_ | Exciton binding energy |

Table S4. Symbols and physical meanings of 5 molecular descriptors (MD) assembled in the MDS−ET

| No. | Symbol | Property |
| --- | --- | --- |
| 43 | λ_h_ | Hole reorganization energy |
| 44 | λ_e_ | Electronic reorganization energy |
| 45 | λ_t_ | Total reorganization energy |
| 46 | *k_inject_* | Kinetics of electron injection |
| 47 | ΔE_S-I_ | Energy difference between singly occupied MO (SOMO) of cationic dye and electrolyte’s (I^−^/I_3_^−^) oxidation potential (−4.8 eV) |

**S1. Computational Methods**

*S1.1 Quantum Chemistry Calculations*

DFT, TD-DFT, and UDFT calculations were performed using the Gaussian 16 program^[49]^. The geometry optimization of all molecules (neutral, anion and cation) are carried through using the Becke-3 parameter Lee-Yang-Parr (B3LYP) exchange-correlation functional with a 6-31G(d,p) basis set^[50-51]^. The conductor-like polarizable continuum model (C-PCM) is used to simulate the environment of tetrahydrofuran (THF)^[52]^. Long alkyl chains are shortened to methyl in the calculation to reduce the computing resource due to their limited contribution to the electronic structures. We calculated all of the MDs for YD2-o-C8 Zn-porphyrin with full alkyl chains to support the above point. We compared them with those of the corresponding molecule where the alkyl chains were substituted with methyl groups. The results show that the MDs are similar (data not shown). We employed TD-DFT at the B3LYP/6-31G(d,p) level using the C-PCM solvent model to calculate the excitation energy, and oscillator strength upon fully optimized structures of individual Zn-porphyrins. Then, the electron density difference maps (EDDMs), which indicate the electron density before and after excitation, were generated using GaussSum3^[53]^.

To model the configurations of dye sensitizers adsorbed on TiO_2_, we calculate the configurations of acceptor groups absorbed on the TiO_2_ surface. The Dmol^3^ software package^[54-55]^ was used. The geometry optimization utilized the Perdew-Burke-Ernzerhof (PBE) parametrization of the generalized gradient approximation^[56]^ (GGA), along with the double numerical basis sets with polarization (DNP) and all-electron core treatment^[57]^. The anatase (101) surface was utilized for dye absorption studies, employing a (TiO_2_)_64_ unit cell with periodic boundary conditions.

*S1.2 Algorithm Description and Assessment Metrics*

In this study, we employed four ML algorithms, namely LGBM, ANN, and CNN , to construct predictive models for the PCE of Zn-porphyrin based DSCs. In order to achieve a reliable and robust predictive model, the entire dataset was randomly divided into two subsets: a training set comprising 90% of the data and a test set comprising the remaining 10%. To ensure an unbiased evaluation of the model, the cross-validation technique was employed during the training phase. Specifically, a 10-fold cross-validation approach was utilized, wherein the dataset was partitioned into ten equal-sized folds. During each iteration, nine folds were used as the training set, while the remaining fold was used as the validation set. This process was repeated ten times, with each fold serving as the validation set exactly once. The performance metrics obtained from each iteration were then averaged to obtain a more robust assessment of the model's predictive capabilities. This 10-fold cross-validation technique was employed to gauge the model's generalization ability and mitigate the impact of potential variations in the data. Subsequently, the bagging technique was applied to the models derived from the 10-fold cross-validation procedure. Bagging, an ensemble technique, harnesses the diversity of multiple models to enhance prediction accuracy, reduce variance, and provide an estimate of the model's generalization error. By aggregating the predictions of multiple models, bagging leverages their individual strengths to improve overall performance. Finally, the independent test set was employed to evaluate the performance of the optimized model. This separate test set, distinct from the training and validation sets, served as a reliable benchmark to assess the model's predictive capabilities on unseen data, thus providing an unbiased measure of its effectiveness.

The assessment includes the computation of three metrics such as the Pearson's correlation coefficient for the validation set (*r*_val_) and the test set (*r*_test_), mean absolute error (*MAE*), and root mean square error (*RMSE*). Pearson's correlation coefficient quantifies the strength and direction of the linear relationship between the variables and is expressed as

$$r= \frac{\sum_{k=1}^{N} \left( A_{k}-\bar{A_{k}} \right)\times\left( P_{k}-\bar{P_{k}} \right)}{\sqrt{\sum_{k=1}^{N} \left( A_{k}-\bar{A_{k}} \right)^{2}} \times\sqrt{\sum_{k=1}^{N} \left( P_{k}-\bar{P_{k}} \right)^{2}}}$$

, where $A_{k}$ is the actual value, $\bar{A_{k}}$ is the average of the actual value and $P_{k}$ is the predicted value, $\bar{P_{k}}$ is the average of the predicted value. The coefficient ranges from -1 to 1, where a value of −1 indicates a perfect negative linear correlation, 1 indicates a perfect positive linear correlation, and 0 indicates no linear correlation. *MAE* measures the average absolute difference between predicted values and actual values. It is less sensitive to outliers and provides a more intuitive measure of error. *RMSE* is the square root of *MSE*, which scales the error back to the original data magnitude and is easier to interpret.

*S1.3 Model Explanation: SHAP value*

For enhancing the interpretability of the ML models, we utilized SHAP (Shapley Additive exPlanations)^[58-59]^, a valuable framework that addresses the challenges posed by the growing complexity of ML models. Originally derived from cooperative game theory, SHAP provides a comprehensive tool for explaining various ML models. For a specific data represented as *x*_i_, where *x*_i,j_ denotes the *j*th MD of the *i*th data, f(*x*_i_) represents the model's predicted value for the *i*th data, and *f*_base_ represents the base value of the model (i.e., the mean of the dependent variable for all data), the SHAP value can be computed using the following equation: $f\left( x_{i} \right)=f_{base}+\sum_{j=1}^{n} \varphi(x_{i,j})$, where φ(*x*_i,j_) signifies the contribution of the *j*th MD in the *i*th data to the predicted value. A positive value of φ(*x*_i,j_) indicates that the *j*th MD enhances the predictive value of the *i*th data instance, while a negative value indicates a reduction in the predictive value. SHAP has the remarkable capability of capturing the influence of each MD on individual data instances, allowing the quantification of both positive and negative contributions.

**S2. Characterization of GY-Series Dyes**

**GY5**: Yield 70%. ^1^H NMR (CDCl_3_/CD_3_OD, 400 MHz) δ 10.95 (s, 1H), 9.83 (d, J = 4.8 Hz, 2H), 9.63 (d, J = 8.8 Hz, 2H), 9.12 (d, J = 4.4 Hz, 2H), 8.89 (d, J = 4.8 Hz, 2H), 8.63 (d, J = 4.4 Hz, 2H), 8.52 (d, J = 7.2 Hz, 2H), 7.84 (d, J = 7.6 Hz, 2H), 7.65 (t, J = 8.4 Hz, 2H), 7.22 (d, J= 8.8 Hz, 4H), 6.97 (d. J= 8.8 Hz, 4H), 6.92 (d, JE 8.8 Hz, 4H), 3.85 (t, J=6.8 Hz,8H), 2.46 (t, J=7.6 Hz, 4H), 1.58-1.51 (m, 4H), 1.29-1.22 (m, 12H), 1.00-0.97 (m, 8H), 0.87-0.82 (m, 8H), 0.74-0.47 (m, 52H). ^13^C NMR (CDCl_3_/CD_3_OD, 400 MHz) δ 160.3, 152.6, 1S2.5, 151.0, 150.9, 150.8, 135.2, 134.1,133.6, 133.1, 132.5, 131.1, 130.6, 130.4, 130.2, 129.2,126.2, 121.5, 106.0, 99.1, 92.2, 69.4,35.7, 32.2, 32.0, 31.9, 29.6, 29.1, 29.0, 25.6, 23.1, 22.7, 14.5, 14.2. ESI-MS: m/z calcd. for C106H125N5O8Zn: 1661, found: 1661.9 [M+H]^+^.

**GY6**: Yield 94%.^1^H NMR (CDCl_3_/CD_3_OD, 400 MHz) δ 9.58 (d, J= 4.8Hz, 2H), 9.06 (d, J= 4.4Hz, 2H), 8.83 (s, 1H), 8.75 (d, J = 4.0Hz, 2H), 8.56 (d, J= 4.4Hz, 2H), 8.43 (d, J= 7.6Hz, 1H), 8.08 (d, J = 8.4Hz, 1H), 7.64 (t, J = 8.0Hz, 2H), 7.17 (d, J = 8.4Hz, 4H), 6.94 (d, J = 8.0Hz, 4H), 6.88 (d, J=8.8Hz,4H), 3.81 (t, J=6.4 Hz, 8H), 2.42 (t, J=7.6 Hz, 4H), 1.47-1.43 (m, 6H), 1.30-1. 19 (m, 16H), 0.95 (t, J=6.8 Hz, 16H), 0.89-0.78 (m, 14H), 0.72-0.48 (m, 36H).

**GY7**: Yield 98%. ^1^H NMR (CDCl_3_, 400 MHz) δ 9.67(d, J = 4.4 Hz,2H), 9.17 (d, J = 4.8 Hz, 2H), 8.87 (d, J= 4.4 Hz, 2H), 8.68 (d, J= 4.4 Hz, 3H), 8.19 (dd, J = 9.2 Hz, 1H), 7.72 (s, 1H), 7.66 (t, J = 8.4 Hz, 2H), 7.54 (d, J = 8.4 Hz, 1H), 7.21 (d, J = 8.4 Hz, 4H), 6.96 (d, J = 8.4 Hz, 4H), 6.93 (d, J = 8.8 Hz, 4H), 3.84 (t, J = 6.4 Hz, 8H), 2.46 (t, J =7.6 Hz, 4H), 1.53 (s, 4H), 1.24 (s, 12H), 1.01 (t, J = 7.6 Hz, 16H), 0.87-0.78 (m, 14H), 0.63-0.45 (m, 36H). ^13^C NMR (CDCl_3_/pyridine-d5, 100 MHz) δ 159.8, 151 .8, 151.3, 150.3, 149.9, 134.6, 133.9, 132.5, 132.0, 131.5, 130.0, 129.2, 128.8, 128.3, 125.0, 122.3, 121.4, 113.6, 108.6, 105.1, 97.6, 94.7, 93.9, 68.4, 53.2, 35.1, 31.6, 31.3, 29.5, 29.0, 28.5, 25.0, 22.4, 22.2, 13.9, 13.7. MALDI-TOF-MS: m/z calcd. for C97H121N5O6Zn: 1517, found: 1518 [M+H]^+^.

**GY8**: Yield 20%. ^1^H NMR (CDCl_3_/pyridine-d5, 400 MHz) δ 9.70 (d, J = 4.8 Hz, 2H), 9.17 (d, J = 4.4 Hz, 2H), 8.88 (d, J = 4.4 Hz, 2H), 8.68 (d, J = 4.8 Hz, 2H), 7.66 (t, 8.4 Hz,2H), 7.20 (d, J = 8.0 Hz, 4H), 6.96(d ,J = 8.4 Hz, 8H), 3.82 (t, J = 6.4 Hz, 8H), 2.44 (t, J = 7.6 Hz, 4H), 1.56 (s, 4H), 1.24 (s, 12H), 0.97 (t, J=7.6 Hz, 16H), 0.83-0.81 (m, 14H), 0.62-0.49 (m, 36H). MALDI-TOF-MS: m/z calcd. for C97H117F4NsO6Zn: 1589, found 1589 [M+H]^+^.

**GY9**: Yield 98%. ^1^H NMR (CDCl_3_/pyridine-d5, 400 MHz) δ 9.62 (d, J = 4.4 Hz, 2H), 9.16 (d, J = 4.8 Hz, 2H), 8.91 (d, J = 4.8 Hz, 2H), 8.66 (d, J = 4.8 Hz, 2H), 7.87 (s, 1H), 7.68 (t, J = 8.0 Hz, 2H), 7.21 (d, J = 8.4 Hz, 4H), 6.99 (d, J = 8.4 Hz, 4H), 6.95 (d, J = 8.4 Hz, 4H), 3.87 (t, J = 6.4 Hz, 8H), 2.47 (t, J = 7.6 Hz, 4H), 1.52 (S, 4H), 1.25 (s, 12H), 1.03 (t, J = 7.6 Hz, 16H), 0.85-0.79 (m, 14H), 0.66-0.45 (m, 36H). ^13^C NMR (CDCl_3_/pyridine-d5, 100 MHz) δ 159.8, 152.3, 151.3, 150.5, 150.4, 134.2, 132.5, 131.5, 130.4, 129.5, 128.4, 121.6, 121.1, 114.8, 105.1, 68.4, 53.3, 35.1, 31.6, 31.4, 29.6, 29.0, 28.6, 25.0, 24.9, 13.8, 13.7. MALDI-TOF-MS: m/z calcd. for C100H118F4N6O6Zn: 1640, found: 1641 [M+H]^+^.

**GY12**: Yield 84%. ^1^H NMR (400 MHz, CDCl_3_/CD_3_OD) δ 9.42 (d, J = 4.4 Hz, 2H), 8.94 (d, J = 4.4 Hz, 2H), 8.66 (d, J = 4.4 Hz, 2H), 8.46 (d, J = 4.4 Hz, 2H), 8.24 (s, 1H), 7.68 (t, J = 8.4 Hz, 2H), 7.09 (d, J = 9.2 Hz, 4H), 7.06 (d, J =8.4 Hz, 4H), 6.95(d, J =8.4 Hz, 4H), 3.83(t, J =6.4 Hz, 8H), 2.42 (t, J = 7.2 Hz, 4H), 1.43-1.38 (m, 4H), 1.23 (br, 12H), 0.99 (t, J =7.6 Hz, 16H), 0.89-0.68 (m, 14H), 0.65-0.48 (m, 36H); ^13^C NMR (100 MHz, CDCl_3_/CD_3_OD) δ 160.1, 159.4, 151.7, 150.7, 147.9, 145.7, 144.4, 131.6, 130.4, 129.7, 128.6, 127.6, 121.9, 108.5, 69.7, 35.2, 31.7, 31.4, 29.6, 29.0, 28.6, 28.4, 28.1, 25.1, 22.5, 22.2, 13.8, 13.6.

**GY13**: Yield 72%. ^1^H NMR (400 MHz, CDCl_3_/CD_3_OD) δ 9.45 (d, J = 4.8 Hz, 2H), 9.02 (d, J = 4.4 Hz, 2H), 8.72 (d, J = 4.4 Hz, 2H), 8.52 (d, J = 4.4 Hz, 2H), 8.15 (s, 1H), 7.61-7.57 (m, 4H), 7.51-7.48 (m, 1H), 7.38 (d, J = 4.0 Hz, 1H), 7.15 (d, J = 8.8 Hz, 4H), 6.91(d, J =8.8 Hz, 4H), 6.84(d, J =8.8 Hz, 4H), 3.79(t, J =6.2 Hz, 8H), 2.38 (t, J = 7.2 Hz, 4H), 1.45-1.30 (m, 4H), 1.28 (br, 12H), 1.19 (t, J =7.6 Hz, 16H), 0.93-0.62 (m, 14H), 0.57-0.46 (m, 36H); ^13^C NMR (100 MHz, CDCl_3_/CD_3_OD) δ 160.5, 159.7, 153.1, 152.9, 151.7, 151.3, 150.6, 136.8, 135.5, 134.4, 133.8, 132.6, 131.8, 131.2, 130.7, 129.9, 129.4, 128.7, 128.3, 127.9, 127.4, 122.8, 114.7, 106.5, 103.2, 91.2, 90.6, 68.3, 43.5, 35.4, 31.2, 30.9, 28.5, 28.1, 27.0, 26.6, 22.1, 21.7, 19.5, 18.6, 14.7

**GY51**: Yield 83%. ^1^H NMR (CDCl_3_/pyridine-d5, 400 MHz) δ 9.49 (d, J = 4.4 Hz, 2H), 8.90 (d, J = 4.4 Hz, 2H), 8.79 (d, J = 4.8 Hz, 2H), 8.58 (d, J = 4.4 Hz, 2H), 8.37 (s, 1H), 7.76 (s, 1H), 7.65-7.58 (m, 3H), 7.02-6.93 (m, 8H), 6.79 (d, J = 8.4 Hz, 4H), 3.81 (t, J = 6.4 Hz, 8H), 2.40 (t, J = 8.0 Hz, 4H), 1.49-1.42 (m, 8H), 1.34 (t, J = 6.8 Hz, 4H), 1.27-1.24 (m, 24H), 1.05-1.00 (m, 4H), 0.96-0.73 (m, 32H), 0.65-0.58 (m, 24H), 0.55-0.42 (m, 12H). ^13^C NMR (CDCl_3_/pyridine-d5, 100 MHz) δ 207.4, 161.0, 160.3, 158.9,152.1, 152.0, 150.8, 150.6,148.9, 148.5,147.2, 146.1, 143.9, 140.4, 138.1, 135.8,134.5, 134.3,132.1, 130.7, 130.0, 128.9, 123.3, 122.0, 121.8, 1183, 114.5, 106.9, 105.5, 102.2, 101.8,97.5,88.6,68.9,35.7,33.3,'32.2, 31.9,31.5,31.3, 29.5, 29.1, 29.0, 27.2, 25.5, 24.5, 23.5, 23.1, 23.0, 22.9, 14.5, 14.4, 12.2. ESI-MS: m/z calcd. for C114H46N6O6S2SiZn: 1852, found 1853.1 [M+H]^+^.

**GY52**: Yield 98%. ^1^H NMR (CDCl_3_/pyridine-d5, 400 MHz) δ 9.53 (d, J = 4.4 Hz, 2H), 8.97 (d, J = 4.4 Hz, 2H), 8.75 (d, J = 4.8 Hz, 2H), 8.56 (d, J = 4.4 Hz, 2H), 8.44 (s, 1H), 7.60 (t, J = 8.4 Hz, 2H), 6.99 (d, J = 7.6 Hz, 4H), 6.92 (d, J = 8.4 Hz, 4H), 6.77 (d, J = 7.6 Hz, 4H), 4.49 (s, 4H), 4.43 (d, J = 3.6 Hz, 2H), 4.39 (d, J = 3.6 Hz, 2H), 3.78 (t, 8H), 2.38 (t, J = 8.0 Hz, 4H), 1.50-1.42 (m, 4H), 1.28-1.19 (m, 12H),0.94-0.79 (m, 32H), 0.63-0.57 (m, 24H), 0.51-0.45 (m, 14H). ^13^C NMR (CDCl_3_/pyridine-d5, 100 MHz) δ 160.3, 151.9, 150.8, 150.7, 150.4, 149.9, 149.6, 149.3, 136.0, 135.8, 135.6, 134.4, 132.0, 131.9, 130.4, 129.8, 128.8, 123.7, 123.5, 123.2, 121.9, 114.3, 111.4, 105.5, 98.4 86.7, 68.8, 65.8, 65.5, 68.8, 65.8, 65.5, 35.6, 32.1, 31.9, 31.8, 29.5, 29.0, 28.9, 25.4, 22.9, 22.7, 14.4, 14.3. ESI: m/z calcd. for C106H126N6O10S2Zn: 1772, found: 1772.9 [M+H]^+^.

**GY58**: Yield: 35%. ^1^H NMR (CDCl_3_/CD_3_OD, 400 MHz) δ 9.52 (d, J = 4.5 Hz, 2H), 9.44 (d, J = 4.4 Hz, 2H, 8.70 (dd, J = 16.6, 4.4 Hz, 4H), 8.31 (s, 1H), 7.79 - 7.70 (m, 3H), 7.68 - 7.55 (m, 3H), 7.10 - 7.03 (m, 10H), 6.94 (d, J = 8.4 Hz, 4H), 3.80 (t, J = 6.5 Hz, 8H), 2.54 (t, J = 8.0 Hz, 4H), 1.63 - 1.53 (m, 4H), 1.38 - 1.22 (m, 12H), 0.98 - 0.88 (m, 8H), 0.88 - 0.74 (m, 16H), 0.71 - 0.60 (m, 8H), 0.60 - 0.45 (m, 26H), 0.45 - 0.34 (m, 8H). ^13^C NMR (101 MHz, CDCl_3_/CD_3_0D) δ 159.7, 151.5, 151.3, 150.5, 150.2, 148.1, 144.8, 138.3, 132.2, 131. 8, 131.2, 129.6, 129.2, 124.9, 121.5, 121. 1, 114.8, 108.7, 105.3, 97.2, 92.5, 68.7, 35.3, 31.6, 31.4, 31.3, 28.9, 28.6, 28.4, 25.1, 22.5, 22.2, 13.9, 13.7.

**GY60**: Yield 30%. ^1^H NMR (CDCl_3_/CD_3_OD, 400 MHz) δ 9.55 (d, J = 4.5 Hz, 2H), 9.47 (d, J= 4.5 Hz, 2H), 8.73 (dd, J = 12.0, 4.5 Hz, 4H), 7.79 (s, 1H), 7.75 (d, J = 8.5 Hz, 2H), 7.65 (t, J= 8.4 Hz, 2H), 7.53 (s, 1H), 7.10 (d, J= 14.7 Hz, 10H), 6.97 (d, J = 8.4 Hz, 4H), 3.82 (t, J = 6.2 Hz, 8H), 2.56 (t, J = 8.0 Hz, 4H), 1.64 - 1.55 (m, 4H), 1.41 - 1.23 (m, 8H), 1.00 - 0.79 (m, 22H), 0.75 - 0.63 (m, 8H), 0.63 - 0.49 (m, 28H), 0.49 - 0.35 (m, 12H). ^13^C NMR (CDCl_3_/CD_3_OD, 400 MHz) δ 159.7, 151.4, 151.3, 150.3, 150.1, 148.1, 144.83, 132.2,131.7, 131.3, 131.2, 130.4,129.8, 129.6,129.2, 124.9, 121.5, 121.1, 116.4,114.5, 105.2, 101.3, 100.0, 97.6, 96.0, 92.5, 87.8,68.7,35.3,31.6, 31.4, 31.3, 29.0, 28.6, 28.4, 25.1, 22.5, 22.2, 14.0, 13.7.

**GY32**: Yield: 40%. ^1^H NMR (400 MHz, CDCl_3_): δ 9.65 (d, J = 4.5 Hz, 2H), 9.60 (d, J = 4.5 Hz, 2H), 8.86 (d, J = 4.5 Hz, 2H), 8.81 (d, J = 4.5 Hz, 2H), 8.24 (d, J = 8.1 Hz, 2H), 8.05 (d, J = 8.1 Hz, 2H), 7.83 (d, J = 9.0 Hz, 2H), 7.70 (t, J = 8.4 Hz, 2H), 6.99 (d, J = 8.4 Hz, 4H), 6.82 (d, J = 9.0 Hz, 2H), 3.74 (d, J = 5.1 Hz, 8H), 3.52 – 3.42 (m, 4H), 1.27 (t, J = 7.1 Hz, 6H), 0.99 – 0.87 (m, 8H), 0.86 – 0.76 (m, 8H), 0.66 – 0.19 (m, 60H), 0.16 – 0.08 (m, 12H). ^13^C NMR (101 MHz, CDCl_3_): δ 160.0, 151.9, 151.4, 150.6, 150.4, 147.5, 133.0, 132.0, 131.2, 130.3, 129.8, 120.6, 115.5, 111.4, 104.7, 71.2, 44.5, 37.1, 31.4, 30.5, 30.2, 29.7, 29.1, 28.2, 26.1, 22.3, 13.9, 13.5, 12.6. ESI-HRMS: m/z calcd. for C101H133N5O6Zn: 1575.95418, found: 1575.95415 [M+H]^+^.

**GY33**: Yield: 32%. ^1^H NMR (400 MHz, CDCl_3_): δ 9.63 (d, J = 4.5 Hz, 2H), 9.59 (d, J = 4.5 Hz, 2H), 8.84 (d, J = 4.5 Hz, 2H), 8.80 (d, J = 4.5 Hz, 2H), 8.23 (d, J = 8.0 Hz, 2H), 8.04 (d, J = 8.0 Hz, 2H), 7.80 (d, J = 8.7 Hz, 2H), 7.68 (t, J = 8.4 Hz, 2H), 6.97 (d, J = 8.4 Hz, 4H), 6.76 (d, J = 8.7 Hz, 2H), 3.72 (d, J = 4.9 Hz, 8H), 3.44 – 3.34 (m, 4H), 1.69 – 1.61 (m, 4H), 1.46 – 1.34 (m, 8H), 1.00 (t, J = 7.4 Hz, 6H), 0.96 – 0.86 (m, 8H), 0.84 – 0.74 (m, 8H), 0.69 – 0.17 (m, 60H), 0.17 – 0.01 (m, 12H). ^13^C NMR (101 MHz, CDCl_3_): δ 160.1, 151.9, 151.5, 150.7, 150.5, 132.9, 132.1, 131.4, 131.3, 130.8, 130.2, 129.8, 111.5, 104.8, 71.3, 50.8, 37.2, 31.4, 30.6, 30.2, 29.6, 29.1, 28.2, 26.1, 22.3, 20.4, 13.9, 13.4. ESI-HRMS: m/z calcd. for C105H141N5O6Zn: 1632.0618, found: 1632.0166 [M+H]^+^.

**GY34**: Yield: 31%. ^1^H NMR (400 MHz, CDCl_3_): δ 9.65 (d, J = 4.5 Hz, 2H), 9.61 (d, J = 4.5 Hz, 2H), 8.86 (d, J = 4.5 Hz, 2H), 8.81 (d, J = 4.5 Hz, 2H), 8.26 (d, J = 8.2 Hz, 2H), 8.06 (d, J = 8.2 Hz, 3H), 7.81 (d, J = 8.6 Hz, 3H), 7.70 (t, J = 8.4 Hz, 2H), 6.99 (d, J = 8.6 Hz, 4H), 6.79 (d, J = 8.6 Hz, 2H), 3.74 (d, J = 5.5 Hz, 8H), 3.33 (d, J = 7.4 Hz, 4H), 1.94 (s, 2H), 1.68 (s, 4H), 1.39 – 1.23 (m, 38H), 0.97 – 0.87 (m, 14H), 0.87 – 0.74 (m, 8H), 0.66 – 0.18 (m, 60H), 0.18 – 0.01 (m, 12H). ^13^C NMR (101 MHz, CDCl_3_): δ 160.0, 151.8, 151.4, 150.4, 148.1, 132.7, 132.0, 131.4, 131.2, 130.8, 130.3, 130.0, 129.7, 120.6, 115.4, 112.4, 110.0, 104.7, 71.2, 56.8, 37.1, 35.5, 31.9, 31.6, 31.4, 31.3, 31.0, 30.5, 30.1, 29.9, 29.7, 29.1, 28.7, 28.2, 26.5, 26.1, 26.0, 23.2, 22.7, 22.3, 14.1, 13.9, 13.5. ESI-HRMS: m/z calcd. for C121H173N5O6Zn: 1856.2672, found: 1856.2662 [M+H]^+^.

**GY45**: Yield: 36%. 'H NMR (400 MHz, CDCl_3_): δ 9.57 (d, J= 4.5 Hz, 2H), 9.54 (d, J = 4.5 Hz, 2H), 8.77 (d, J= 4.5 Hz, 2H), 8.74 (d, J = 4.5 Hz, 2H, 8.20 (d, J = 8.2 Hz, 2H, 8.00 (d, J= 8.2 Hz, 2H), 7.82 (d, J - 8.7 Hz, 2H), 7.67 (t, J = 8.4 Hz, 2H), 7.02 (d, J = 8.7 Hz, 2H), 6.97 (d, J= 8.4 Hz, 4H), 3.72 (d, J - 5.6 Hz, 8H), 3.26 (t, J = 5.6 Hz, 4H), 1.77 - 1.69 (m, 4H, 1.66 - 1.59 (m, 2H), 0.96 - 0.83 (m, 12H), 0.74 - 0.64 (m, 8H), 0.60 - 0.38 (m, 44H), 0.38 - 0.27 (m, 16H), 0.27 - 0.20 (m, 12H). ^13^C NMR (101 MHz, CDCl_3_): δ 160.1, 151.8, 151.6, 150.6, 150.4, 131.9, 131.3, 131.0, 130.8, 130.2, 129.7, 120.8, 115.4, 115.3, 104.7, 71.3, 37.1, 31.6, 30.5, 30.2, 29.7, 29.2, 28.3, 26.2, 26.1, 22.4, 14.0, 13.6. ESI(HRMS): m/z calcd. for C102H133O6N5Zn: 1587.9542, found: 1587.9588 [M+H]^+^.

**GY47**: Yield 89%. ^1^H NMR (CDCl_3_, 400 MHz): δ 9.78 (d, J = 4.4 Hz, 2H), 9.62 (d, J = 4.4 Hz, 2H), 8.95 (d, J = 8.3 Hz, 1H), 8.88 (d, J = 13.0 Hz, 4H), 8.36 (dd, J = 7.8, 4.8 Hz, 2H), 8.33 – 8.18 (m, 5H), 8.05 (d, J = 8.0 Hz, 2H), 7.81 – 7.67 (m, 5H), 7.52 (dd, J = 13.8, 7.7 Hz, 2H), 7.02 (d, J = 8.4 Hz, 4H), 3.77 (d, J = 5.5 Hz, 8H), 1.31 (s, 4H), 1.00 – 0.74 (m, 8H), 0.74 – 0.62 (m, 8H), 0.62 – 0.39 (m, 45H), 0.39 – 0.29 (m, 15H), 0.29 – 0.17 (m, 12H). ^13^C NMR (CDCl_3_, 101 MHz): δ 160.0, 151.7, 151.5, 150.7, 135.1, 131.8, 131.2, 131.0, 130.3, 128.9, 127.4, 126.7, 120.9, 120.6, 120.1, 104.7, 71.1, 37.2, 30.4, 30.1, 29.7, 29.1, 28.2, 26.0, 22.4, 13.9, 13.6. ESI-HRMS (m/z) Calcd for C111H132N4O6Zn: 1680.9433; Found: 1680.9365 [ M + H ]^+^.

**GY48**: Yield 92%. ^1^H NMR (CDCl_3_, 400 MHz): δ 9.95 (d, J = 4.5 Hz, 2H), 9.66 (d, J = 4.5 Hz, 2H), 9.16 (d, J = 8.1 Hz, 1H), 8.97 (d, J = 4.5 Hz, 2H), 8.92 (d, J = 4.5 Hz, 2H), 8.83 (d, J = 7.6 Hz, 1H) , 8.75 (d, J = 7.6 Hz, 1H), 8.53 (s, 1H), 8.28 (d, J = 8.0 Hz, 2H), 8.16 (d, J = 8.0 Hz, 1H), 8.13 – 8.05 (m, 3H), 7.98 (d, J = 8.8, 1H), 7.89 – 7.81 (m, 2H), 7.73 (t, J = 8.5 Hz, 2H), 7.02 (d, J = 8.5 Hz, 4H), 4.70 (d, J = 6.9 Hz, 2H), 3.78 (d, J = 5.1 Hz, 8H), 2.42 (s, 1H), 1.56 – 1.2 (m, 20H), 1.01 – 0.73 (m, 22H), 0.66 – 0.19 (m, 60H), 0.19 – 0.01 (m, 12H). ^13^C NMR (CDCl_3_, 101 MHz): δ 160.0, 151.8, 151.5, 150.6, 150.5, 133.4, 131.8, 131.5, 131.1, 130.4, 130.1, 129.7, 128.7, 125.2, 124.6, 120.9, 118.1, 117.3, 115.1, 113.4, 104.7, 96.1, 71.2, 40.0, 37.1, 31.8, 31.5, 30.4, 30.1, 29.6, 29.1, 28.8, 28.2, 26.6, 26.1, 23.0, 22.6, 22.4, 14.0, 13.9, 13.6. ESI-HRMS (m/z) Calcd for C123H155N5O6Zn: 1862.3801; Found: 1862.1199 [ M + H ]^+^.


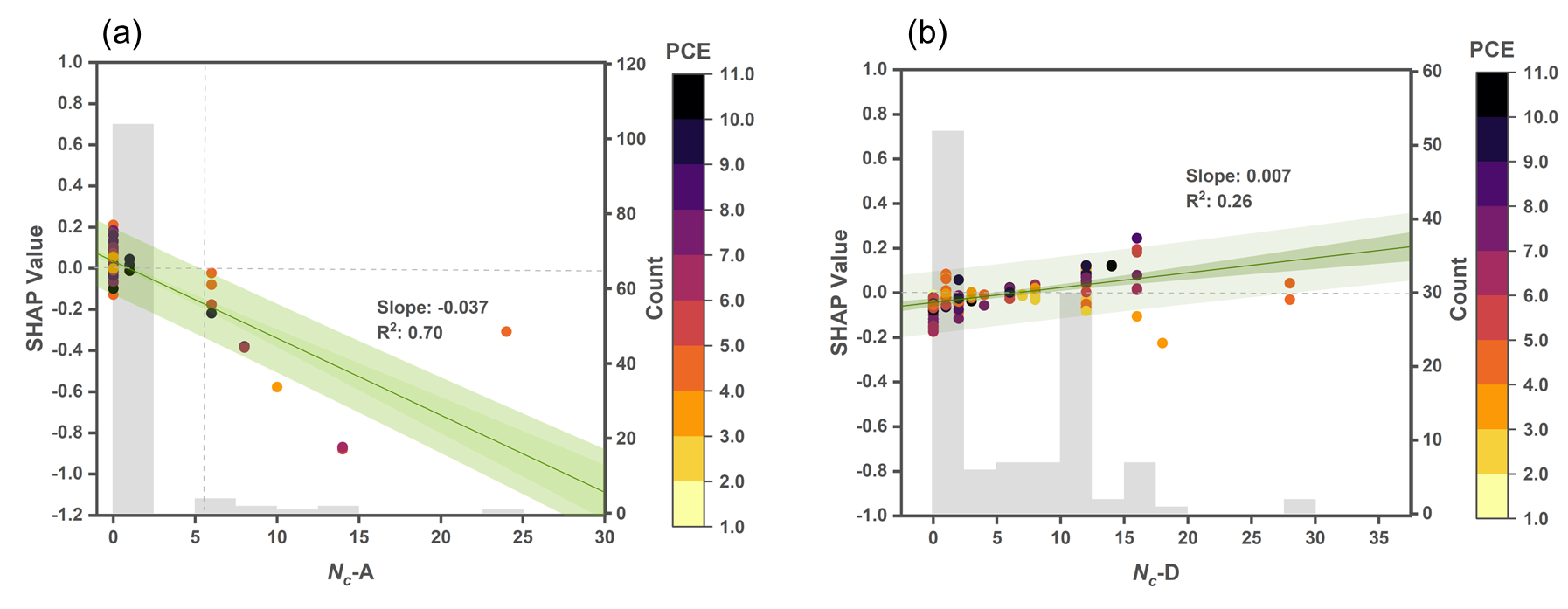


Figure S2. Distribution plots correlating the (a) *N_C_*-D and (b) *N_C_*-A values with their respective SHAP values based on the optimized CNN/MDS−GS+ABS+ET model. The figure displays linear fitting results, including the slope and *R^2^* value. The dark-colored narrow band represents the 95% confidence interval, while the light-colored wide band indicates the 95% prediction interval.

Table S5. Photovoltaic parameters of DSCs based on 17 **GY dyes** under simulated AM 1.5G illumination.*^a^*

| Dye | *J_sc_* [mA cm^-2^] | *V_oc_* [V] | *FF* | PCE [%] |
| --- | --- | --- | --- | --- |
| **GY5** | 10.94 | 0.670 | 0.59 | 4.34 |
| **GY6** | 14.50 | 0.712 | 0.65 | 6.70 |
| **GY7** | 12.10 | 0.773 | 0.69 | 6.39 |
| **GY8** | 15.37 | 0.679 | 0.73 | 7.57 |
| **GY9** | 5.91 | 0.631 | 0.71 | 2.68 |
| **GY12** | 18.3 | 0.650 | 0.60 | 7.06 |
| **GY13** | 15.3 | 0.668 | 0.61 | 6.23 |
| **GY32** | 19.1 | 0.750 | 0.72 | 10.28 |
| **GY33** | 16.0 | 0.717 | 0.71 | 8.16 |
| **GY34** | 16.3 | 0.709 | 0.73 | 8.38 |
| **GY45** | 15.5 | 0.710 | 0.75 | 8.47 |
| **GY47** | 11.0 | 0.702 | 0.67 | 5.32 |
| **GY48** | 11.45 | 0.71 | 0.72 | 6.01 |
| **GY51** | 9.79 | 0.696 | 0.72 | 5.13 |
| **GY52** | 11.20 | 0.668 | 0.69 | 5.37 |
| **GY58** | 11.20 | 0.656 | 0.70 | 5.13 |
| **GY60** | 14.0 | 0.708 | 0.69 | 6.88 |

*^a^*Photovoltaic parameters of the best cells.

Table S6. The predicted PCE values for 17 newly designed Zn Porphyrin-sensitized solar cells (as illustrated in Figure 2) by 12 ML models developed in this study.

| Dye | Exp. PCE  (%) | | Pred. PCE (%) utilizing MDS−GS | | | | Pred.PCE (%) utilizing MDS−GS+ABS | | | Pred.PCE (%) utilizing MDS−GS+ABS+ET | | |
| --- | --- | --- | --- | --- | --- | --- | --- | --- | --- | --- | --- | --- |
|  |  |  | LGBM | ANN | CNN | LGBM | | ANN | CNN | LGBM | ANN | CNN |
| **GY5** | | 4.34 | 5.95 | 6.15 | 6.26 | 6.28 | | 5.65 | 5.88 | 6.47 | 5.30 | 5.62 |
| **GY6** | | 6.7 | 6.41 | 7.47 | 7.35 | 6.77 | | 6.87 | 7.26 | 6.98 | 6.71 | 7.06 |
| **GY7** | | 6.39 | 5.09 | 6.86 | 6.49 | 5.04 | | 5.13 | 5.26 | 5.28 | 5.21 | 5.53 |
| **GY8** | | 7.57 | 6.60 | 7.35 | 7.50 | 6.96 | | 6.66 | 6.98 | 6.99 | 6.31 | 6.76 |
| **GY9** | | 2.68 | 6.13 | 6.16 | 6.25 | 5.60 | | 5.28 | 5.01 | 5.58 | 4.85 | 4.37 |
| **GY12** | | 7.06 | 5.73 | 5.89 | 5.51 | 6.31 | | 6.27 | 6.00 | 6.37 | 6.22 | 6.24 |
| **GY13** | | 6.23 | 6.60 | 6.08 | 6.25 | 6.61 | | 6.44 | 6.51 | 6.55 | 6.17 | 6.55 |
| **GY32** | | 10.28 | 8.15 | 8.11 | 8.48 | 7.86 | | 8.22 | 8.04 | 7.84 | 8.00 | 8.01 |
| **GY33** | | 8.16 | 8.16 | 8.35 | 8.31 | 7.86 | | 8.29 | 7.90 | 7.73 | 8.03 | 8.06 |
| **GY34** | | 8.38 | 8.18 | 8.80 | 8.30 | 7.92 | | 8.50 | 7.89 | 7.78 | 7.90 | 8.04 |
| **GY45** | | 8.47 | 8.30 | 7.69 | 8.25 | 8.62 | | 8.33 | 8.43 | 9.14 | 8.83 | 8.14 |
| **GY47** | | 5.32 | 8.39 | 7.31 | 6.87 | 9.08 | | 7.97 | 8.20 | 9.14 | 7.52 | 7.72 |
| **GY48** | | 6.01 | 8.17 | 8.26 | 8.52 | 8.96 | | 8.74 | 9.07 | 8.91 | 8.28 | 8.87 |
| **GY51** | | 5.13 | 6.65 | 5.39 | 4.85 | 7.07 | | 6.47 | 5.96 | 6.96 | 5.95 | 5.40 |
| **GY52** | | 5.37 | 6.73 | 5.89 | 5.78 | 7.60 | | 6.81 | 6.99 | 7.43 | 6.58 | 6.19 |
| **GY58** | | 5.13 | 6.57 | 6.47 | 6.71 | 6.79 | | 6.38 | 6.31 | 6.59 | 5.85 | 6.31 |
| **GY60** | | 6.88 | 7.34 | 7.66 | 8.33 | 8.10 | | 7.80 | 8.16 | 8.08 | 7.34 | 7.97 |

**
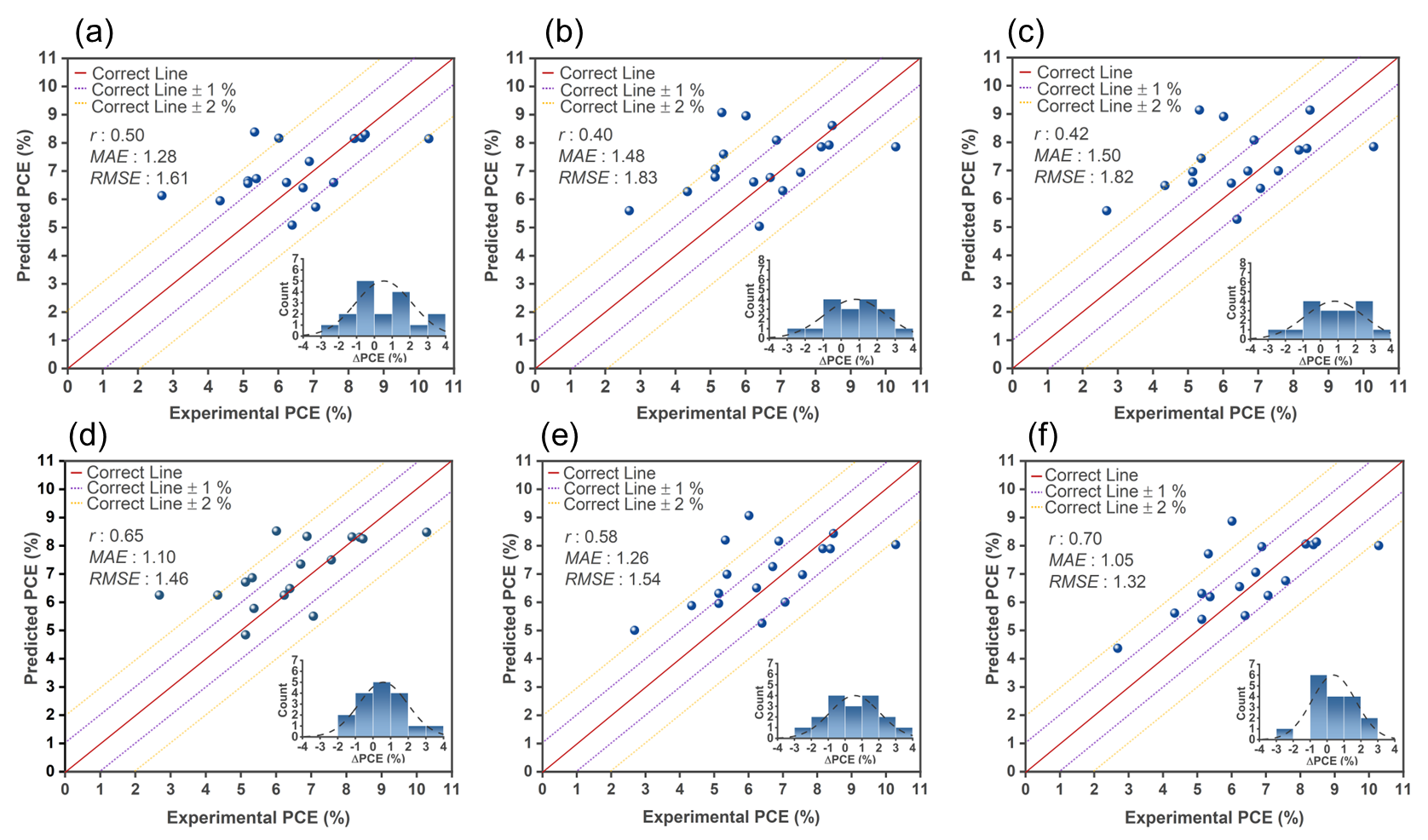
**

Figure S3. Performance assessment and predicted PCEs of 17 newly designed Zn porphyrin-sensitized solar cells compared to their experimentally measured values using various ML Models, including (a). LGBM/MDS-GS, (b). LGBM/MDS-GS+ABS, (c). LGBM/MDS-GS+ABS+ET, (d). CNN/MDS-GS, (e). CNN/MDS-GS+ABS, and (f). CNN/MDS-GS+ABS+ET. The inset figure displays the distribution of prediction errors (ΔPCE) calculated as the predicted PCE minus the experimental PCE.

**S3. Working Principles of DSC**

A typical DSCs includes the following key components:

(I) Transparent Electrode: The DSCs incorporates a transparent conductive electrode, commonly composed of fluorine-doped tin oxide (FTO).

(II) Semiconductor: A thin film of porous semiconductor material, usually titanium dioxide (TiO_2_), is sintered on the transparent electrode.

(III) Sensitizer/Dye: A sensitizer or dye is chemically adsorbed onto the semiconductor surface to facilitate light harvesting.

(IV) Redox Electrolyte: A liquid electrolyte containing a redox couple occupies the space between the semiconductor layer and a counter electrode, enabling the regeneration of oxidized sensitizers.

(V) Counter Electrode: The counter electrode, typically made of catalytic material such as platinum coated onto a transparent glass substrate, plays a crucial role in reducing the oxidized redox electrolyte.

In DSCs, the PCE depends on short-circuit current density (*J_sc_*), open-circuit voltage (*V_oc_*), and fill factor (*FF*). The operational mechanism of DSCs have the following essential processes^[60]^:

(1) Photoexcitation: Incident sunlight excites the dye adsorbed on the semiconductor titanium dioxide from the ground state (Dye) to the excited state (Dye*).

(2) Electron Injection: Unstable excited state electrons are spontaneously injected into the conduction band of TiO_2_ due to the potential difference between the conduction band of TiO_2_ and Dye*.

(3) Electron Transport: The injected electrons diffuse through the mesoporous TiO_2_ film towards the FTO substrate and then transfer to the platinized counter electrode through an external circuit.

(4) Reduction of Redox Agent: With the catalytic assistance of Pt, the oxidized form of the redox agent in the electrolyte is reduced back to its reductive state.

(5) Sensitizer Regeneration: The oxidized sensitizer (Dye^+^) is regenerated by the reductive agent in the electrolyte, completing the cycle.

**S4. Calculations of Significant Molecular Descriptors**

*Simulated Maximum Short-Circuit Current Density* (*J_sc_cal_*)

The *J_SC_* value is primarily determined by the light-harvesting ability of dye, which is directly associated with its absorption spectra. In this study, we employed TD-DFT calculations to simulate the absorption spectra of the dye. The equation for the simulated short-circuit current( *J_sc_cal_*) is expressed as follows^[61]^:

$J_{SC\_cal}=e\int_{0}^{\infty} IPCE\left( \lambda\right)\times\varphi_{AM1.5G}\left( \lambda\right)d\lambda$ (S1)

,where $\varphi_{AM1.5G}$ corresponds to the photon flux under AM1.5G solar irradiation spectra, IPCE is the incident photon-electron conversion efficiency, which means that the process of photoelectric conversion; IPCE is expressed as the following equation^[62]^:

*IPCE*$=LHE \times\phi_{inj}\times\phi_{reg}$ $\times\phi_{coll}$ (S2)

,where $\phi_{inj}$ represents electron injection efficiency, $\phi_{reg}$ represents dye regeneration efficiency, and $\phi_{coll}$ represents charge collection efficiency. Assuming that all these three parameters remain constant and equal to 1 in calculations, under ideal conditions, the solar cell can achieve the maximum short-circuit current density. LHE is expressed as follows^[62]^:

$\mathrm{LHE}\left( \lambda\right)=1- {10}^{-\varepsilon(\lambda)bc}$ (S3)

,where $\varepsilon(\lambda)$ represents the molar absorption coefficient, considering the AM1.5G standard solar spectrum (280-1200 nm), *b* represents the thickness of the TiO_2_ layer, and *c* represents the dye concentration; for ease of calculations, empirical values of 10 μm for *b* (thickness) and 100 mmol/L for *c* (dye concentration) are used.

*Electron Density Difference Map (EDDM)*

Electron Density Difference Map (EDDM)^[63]^ calculated the electron density difference between the ground- and the excited-states, where the excited electron density is coming from and going to of absorption bands upon photoexcitation. We employ it to count the electron density transfer towards the anchoring group (COOH and CN). The calculations were performed using the GaussSum 3.0 program^[64]^.

*The Free Energy of Electron Injection* (*∆G_inj_*)

In DSCs, the rate and efficiency of electron injection processes are characterized by free energy change (*∆G_inj_*). The initial state of the reaction corresponds to the transition of the dye from the ground state to the excited state upon light excitation, and the final state corresponds to the injection of an electron into the conduction band (CB) of the TiO_2_ semiconductor. The energy difference between the initial and final states corresponds to ΔG_inj_ of the reaction. Thus, ΔG_inj_ can be expressed as the following equation^[65]^:

${\text{Δ}\text{G}}_{\mathrm{inj}}=E^{\mathrm{dye}^{*}}-E_{\mathrm{CB}}^{TiO2}$ (S4)

$E^{\mathrm{dye}^{*}}=E^{\mathrm{dye}}+E_{00}$ (S5)

,where $E_{CB}^{TiO2}$ is the conduction band energy of TiO_2_ (-4.00 eV), $E^{{dye}^{*}}$ is the oxidation potential of the excited state of the dye, $E^{dye}$ is the oxidation potential of the ground-state of the dye, and $E_{00}$ is the vertical excitation energy of the lowest energy.

*Excited State Lifetime* ($t_{es}$)

The efficiency of electron injection to TiO_2_ can be determined by the excited state lifetime. A short electron lifetime, will lead to electron recombination. This process would minimize the photovoltage and lower the charge collection efficiency, thereby reducing the *J_SC_* and PCE. After electron injection, the dye was in a cationic state until regeneration occurred. The longer the excited state lifetime, the longer the dyes remained in the cationic form, which favored charge transfer. *t_es_* of the dye can be expressed as follows^[66]^:

$t_{es}= \frac{1.499}{f\left( E_{00} \right)^{2}}$ (S6)

,where $f$ is the oscillator strength of the excited-state and $E_{00}$ is the lowest energy of vertical excitation energy.

*Exciton binding energy* (*E_b_*)

E_b_ is directly related to the charge separation in DSCs, and hence, it is an important factor for the efficiency of the cells. After the dye is irradiated by sunlight, the generated electron-hole pairs will be attractive to each other. Therefore, to avoid charge recombination and reduce the charge collection efficiency, a lower E_b_ is desired. E_b_ can be viewed as the difference between the fundamental and optical bandgap energies. Therefore, E_b_ is expressed as the follows^[67]^:

$E_{b}=E_{\mathrm{fund}}-E_{\mathrm{opt}}$ (S7)

$E_{\mathrm{fund}}=E_{\mathrm{LUMO}}-E_{\mathrm{HOMO}}$ (S8)

,where $E_{fund}$ is the HOMO-LUMO energy bandgap and $E_{opt}$ is optical bandgap, which is taken to be the first excitation energy ($E_{00}$).

*Kinetics of Electron Injection*

Based on Marcus’ theory^[68]^, and the electron-hole transfer rate (*k*)^[69]^ can be expressed as:

$k=A exp\left[ -\frac{\lambda}{4k_{B}T} \right]$ (S9)

,where A is a pre-exponential factor, $k_{B}$ is the Boltzmann constant, $\lambda$ is the reorganization energy and $T$ is the temperature. Figure S4 shows the calculation of the reorganization energy from the neutral and cation or anion potential energy, thus $\lambda$ can be expressed as follows^[70]^:

$\lambda_{h}=\left[ E_{0}^{+}-E_{+} \right]+\left[ E_{+}^{0}-E_{0} \right]$ (S10)

$\lambda_{e}=\left[ E_{0}^{-}-E_{-} \right]+\left[ E_{-}^{0}-E_{0} \right]$ (S11)

,where $E_{0}$ denotes the ground state energy of the optimized neutral dye, $E_{+} \mathrm{and}E_{-}$ denote the energies of optimized structures of cation and anion, respectively, $E_{0}^{+} \mathrm{and} E_{0}^{-}$ denote the energies of cation and anion based on the optimized structure of neutral dye, respectively, and $E_{+}^{0}\mathrm{and}E_{-}^{0}$ denote the energies of neutral states based on the optimized structures of cationic and anionic states, respectively. The *k_inject_* value is calculated by setting the A value to unity.


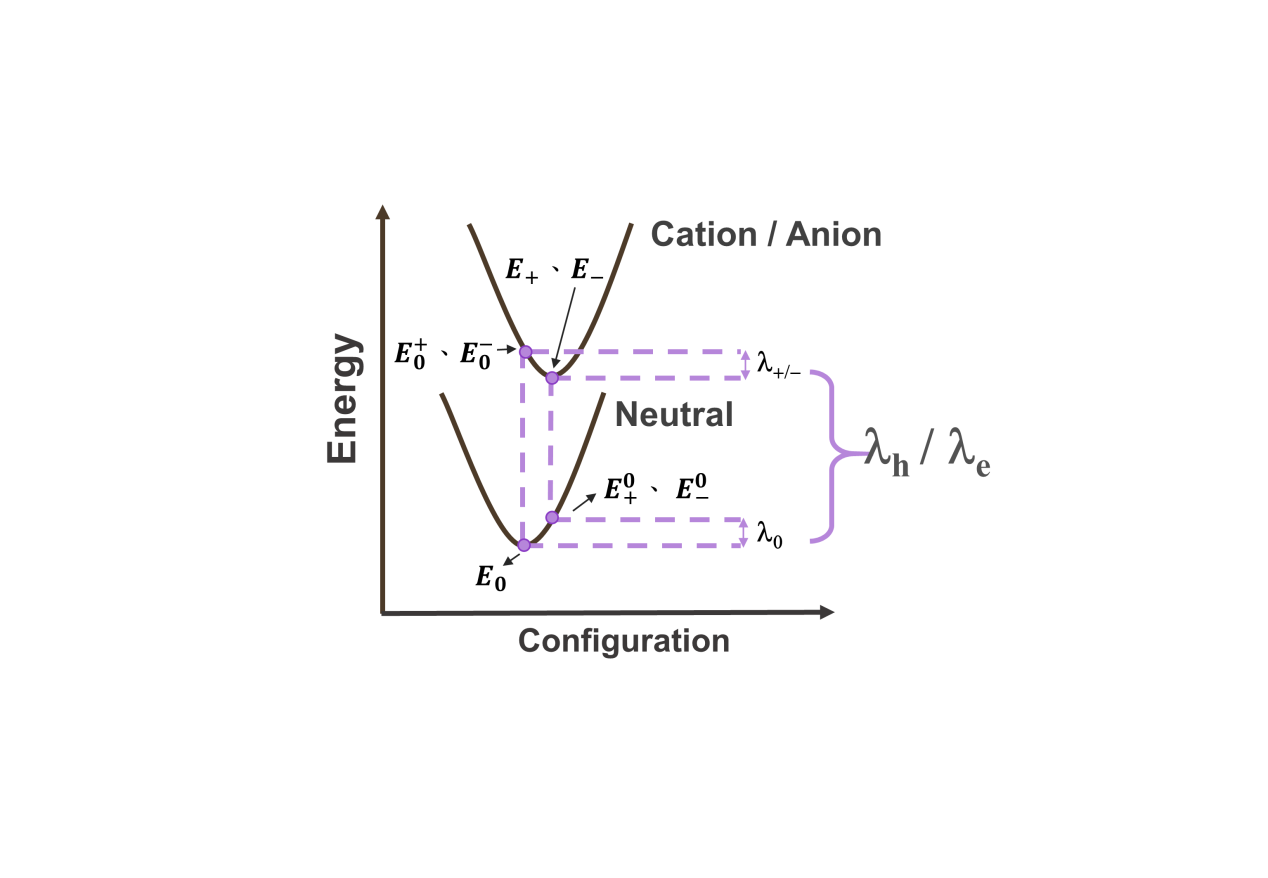


Figure S4 Scheme of the reorganization energy.

**S5. Machine Learning Algorithms**

In this study, we use light gradient boosting machine (LGBM)^[71]^, artificial neural network (ANN)^[72]^ and convolutional neural network (CNN)^[73]^ to construct ML models. Their principles are briefly introduced below:

*S5.1 LGBM*

LGBM utilizes the gradient boosting (GB) framework, which is an ensemble learning technique that combines multiple weak predictive models typically decision trees (DT), see Figure S5 to construct a strong predictive model. It employs the gradient-based learning algorithm, where each new model is trained to correct the errors made by the previous models in the ensemble. In the typical GBDT, the DT is grown layer by layer, treating the leaves of the same layer equally when perform splitting. However, this approach doesn't consider the split gains of individual leaves. LGBM addresses this issue by using a leaf-wise growth strategy, which is more efficient.

In LGBM's leaf-wise strategy, instead of splitting the leaves of the same layer at the same time, it chooses the leaf with the largest split gain and splits it. This process is repeated iteratively. By focusing on leaves with higher split gains, LGBM avoids wasting resources on unnecessary splits in leaves with low split gains. The leaf-wise strategy gives several advantages. It improves training efficiency as it can quickly identify and split leaves that contribute the most to reducing the loss function. The leaf-wise strategy can result in better prediction accuracy by capturing finer-grained patterns and utilizing resources more effectively.


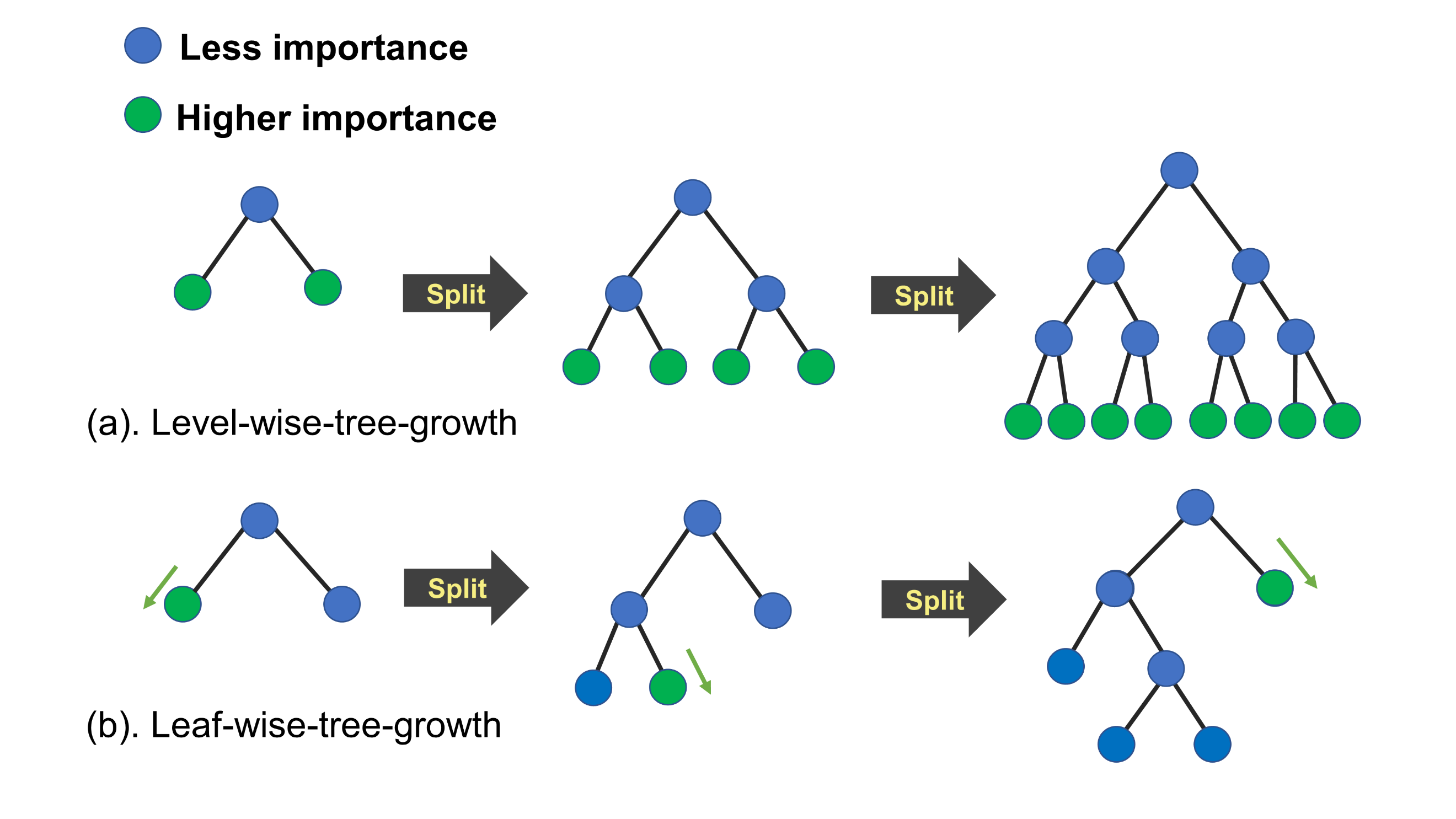


Figure S5. Different ways of growing decision trees: (a) Level-wise-tree-growth;

(b) Leaf-wise-tree-growth.

*S5.2 ANN*

ANNs are a class of ML models inspired by the structure and function of the human brain. ANNs are composed of interconnected nodes, called artificial neurons or "units," which mimic the behavior of biological neurons see Figure S6. These units are organized into layers, with an input layer to receive the input data, one or more hidden layers for intermediate processing, and an output layer to produce the targeted output.

ANNs are trained using a process called "backpropagation," which involves feeding input data into the network, propagating it forward through the layers, comparing the predicted output with the actual values, and then adjusting the weights of the connections between the units to minimize the prediction error. This iterative process continues until the network achieves satisfactory performance.

One of the key strengths of ANNs is their ability to learn complex patterns and relationships in data, enabling them to solve a wide range of complex tasks, including classification, regression, and even more advanced tasks like image and speech recognition. They are highly flexible and can model non-linear relationships between input and output variables.


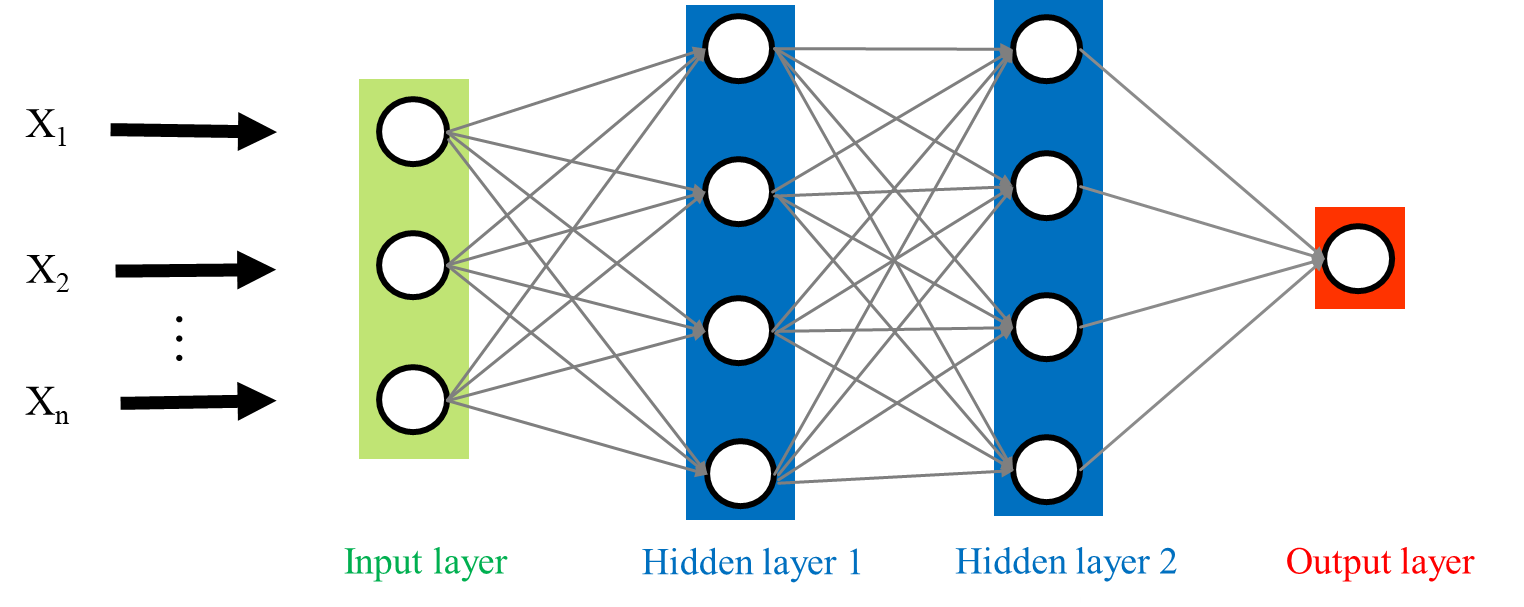


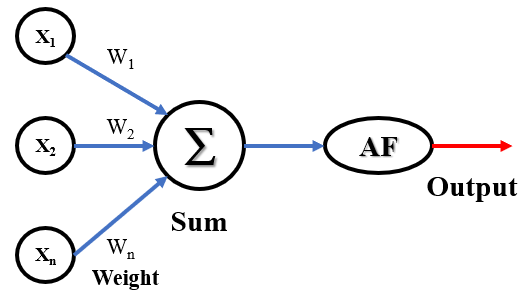

$$f\left( x \right)=\sum_{i=1}^{i=n} W_{n}X_{n}$$

Figure S6 (a) ANN model structure; (b) The learning process of nodes.

*S5.3 CNN*

One dimensional Convolutional Neural Networks (1D CNNs) are a variation of the traditional CNN architecture specifically designed to process 1D sequential data, such as time series or text data. While traditional CNNs operate on two-dimensional inputs like images.

In a 1D CNN, the convolutional layer applies filters to the input sequence, sliding across the data and performing convolutions to extract local patterns see Figure S7. The filters capture features and patterns at different positions in the sequence. By utilizing multiple filters, the network can learn a diverse set of features. The output of the convolutional layer is a set of feature maps that preserve the sequential order of the input data.

To reduce the dimensionality and extract the most salient information, pooling layers are often employed in 1D CNNs. Max pooling is commonly used, which downsamples the feature maps by selecting the maximum value within a fixed window. This operation helps to capture the most relevant features and achieve translation invariance. Following the convolutional and pooling layers, fully connected layers can be added to further process the extracted features and make predictions. These layers take the flattened feature maps as input and perform typical neural network operations, such as nonlinear transformations and classification/regression tasks.


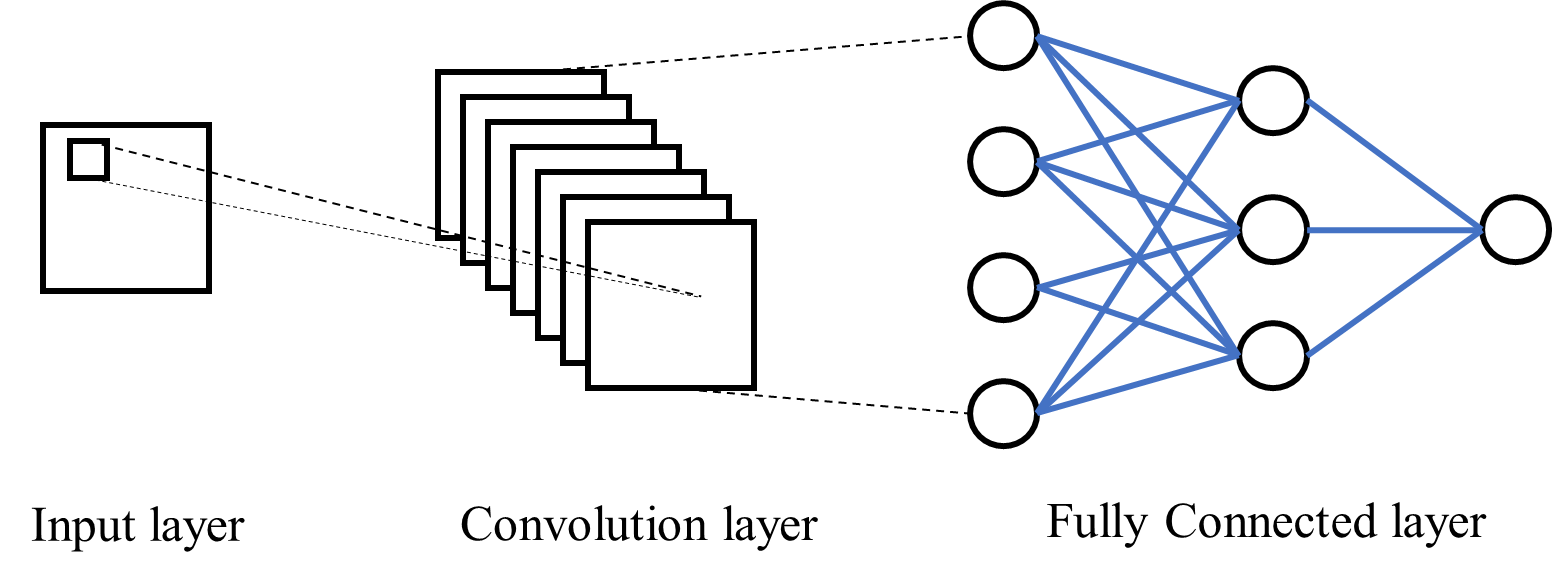


Figure S7 CNN model structure.

**S6. Optimal hyper-parameters**

Table S7. The hyperparameter values for the ML models using MDS-GS.

| LGBM/MDS−GS |
| --- |
| The rate at which weights are updated during each estimators: *learning_rate* = *0.1*  Number of boosting estimators to perform: *n_estimators* = *300 (callbacks)*  Maximum depth of the individual regression estimators: *max_depth* = *3*  The number of leaf nodes in each estimators: *num_leaves* = *4*  The minimum of samples required for each leaf node: *min_child_samples* = *18*  The fraction of data randomly selected for each estimators: *bagging_fraction* = *0.6*  The number of iterations at which bagging is performed: *bagging_freq* = *3*  The strength of L1 regularization: *reg_alpha = 0.7*  The strength of L2 regularization: *reg_lambda = 0.1* |
| ANN/MDS−GS |
| Number of hidden layer: *4*  Number of hidden units in each layer: *24; 24; 12; 12*  Activation function for the each hidden layer: activation = *relu; relu; relu; linear*  The solver for weight optimization: *optimizer = Adam*  An epoch refers to the process of passing the entire dataset through the neural network once and updating the weights: *epochs = 1000 (callbacks)*  The number of data samples to be processed before updating the model's weights:  *batch_size = 32* |
| CNN/MDS−GS |
| Number of 1D Convolutional Layer: *2*  Number of units kernels in each Conv1D layer: *12; 8*  Number of units kernels size in each Conv1D layer: *kernel_size = 3; 5*  Number of 1D Max Pooling Layer: *1*  Number of units pool size in each MaxPooling1D layer: *pool_size = 2*  Number of 1D hidden Layer: *3*  Number of hidden units in each layer: *16; 8; 1*  Activation function for the each hidden layer: activation = *relu; relu; linear* The solver for weight optimization: *optimizer = Adam*  An epoch refers to the process of passing the entire dataset through the neural network once and updating the weights: *epochs = 1000 (callbacks)*  The number of data samples to be processed before updating the model's weights:  *batch_size = 32* |

Table S8. The hyperparameter values for the ML models using MDS-GS+ABS.

| LGBM/MDS−GS+ABS |
| --- |
| The rate at which weights are updated during each estimators: *learning_rate* = *0.1*  Number of boosting estimators to perform: *n_estimators* = *300 (callbacks)*  Maximum depth of the individual regression estimators: *max_depth* = *3*  The number of leaf nodes in each estimators: *num_leaves* = *4*  The minimum of samples required for each leaf node: *min_child_samples* = *18*  The fraction of data randomly selected for each estimators: *bagging_fraction* = *0.7*  The number of iterations at which bagging is performed: *bagging_freq* = *3*  The strength of L1 regularization: *reg_alpha = 0.6*  The strength of L2 regularization: *reg_lambda = 0.1* |
| ANN/MDS−GS+ABS |
| Number of hidden layer: *4*  Number of hidden units in each layer: *43; 43; 22; 10*  Activation function for the each hidden layer: activation = *relu; relu; relu; linear*  The solver for weight optimization: *optimizer = Adam*  An epoch refers to the process of passing the entire dataset through the neural network once and updating the weights: *epochs = 1000 (callbacks)*  The number of data samples to be processed before updating the model's weights:  *batch_size = 32* |
| CNN/MDS−GS+ABS |
| Number of 1D Convolutional Layer: *2*  Number of units kernels in each Conv1D layer: *20; 8*  Number of units kernels size in each Conv1D layer: *kernel_size = 3; 5*  Number of 1D Max Pooling Layer: *1*  Number of units pool size in each MaxPooling1D layer: *pool_size = 2*  Number of 1D hidden Layer: *3*  Number of hidden units in each layer: *16; 8; 1*  Activation function for the each hidden layer: activation = *relu; relu; linear*  The solver for weight optimization: *optimizer = Adam*  An epoch refers to the process of passing the entire dataset through the neural network once and updating the weights: *epochs = 1000 (callbacks)*  The number of data samples to be processed before updating the model's weights:  *batch_size = 32* |

Table S9. The hyperparameter values for the ML models using MDS−GS+ABS+ET ML.

| LGBM/MDS−GS+ABS+ET |
| --- |
| The rate at which weights are updated during each estimators: *learning_rate* = *0.1*  Number of boosting estimators to perform: *n_estimators* = *300 (callbacks)*  Maximum depth of the individual regression estimators: *max_depth* = *3*  The number of leaf nodes in each estimators: *num_leaves* = *4*  The minimum of samples required for each leaf node: *min_child_samples* = *18*  The fraction of data randomly selected for each estimators: *bagging_fraction* = *0.7*  The number of iterations at which bagging is performed: *bagging_freq* = *3*  The strength of L1 regularization: *reg_alpha = 0.7*  The strength of L2 regularization: *reg_lambda = 0.1* |
| ANN/MDS−GS+ABS+ET |
| Number of hidden layer: *4*  Number of hidden units in each layer: *48; 48; 24; 10*  Activation function for the each hidden layer: activation = *relu; relu; relu; linear*  The solver for weight optimization: *optimizer = Adam*  An epoch refers to the process of passing the entire dataset through the neural |
| network once and updating the weights: *epochs = 1000 (callbacks)*  The number of data samples to be processed before updating the model's weights:  *batch_size = 3* |
| CNN/MDS−GS+ABS+ET |
| Number of 1D Convolutional Layer: *2*  Number of units kernels in each Conv1D layer: 20*; 8*  Number of units kernels size in each Conv1D layer: *kernel_size = 3; 5*  Number of 1D Max Pooling Layer: *1*  Number of units pool size in each MaxPooling1D layer: *pool_size = 2*  Number of 1D hidden Layer: *3*  Number of hidden units in each layer: *16; 8; 1*  Activation function for the each hidden layer: activation = *relu; relu; linear*  The solver for weight optimization: *optimizer = Adam*  An epoch refers to the process of passing the entire dataset through the neural network once and updating the weights: *epochs = 1000 (callbacks)*  The number of data samples to be processed before updating the model's weights:  *batch_size = 32* |

Table S10. Coordinates of 2-cyano-3-(5-(prop-1-yn-1-yl)thiophen-2-yl)propanoic acid adsorbed onto a (TiO_2_)_64_ surface in a tridentate fashion.

| Ti | -0.079 | 1.047 | 4.546 | Ti | 8.977 | 4.883 | 0.993 | O | 13.846 | 10.653 | 5.135 |
| --- | --- | --- | --- | --- | --- | --- | --- | --- | --- | --- | --- |
| Ti | 10.478 | 1.074 | 4.517 | Ti | 5.243 | 6.81 | 4.612 | O | 15.28 | 10.646 | 2.452 |
| Ti | -0.075 | 4.889 | 4.545 | Ti | 6.58 | 6.807 | 1.587 | O | 11.696 | 8.739 | 5.811 |
| Ti | 10.347 | 4.964 | 4.424 | Ti | 2.762 | 4.906 | 4.954 | O | 15.336 | 8.73 | 4.535 |
| Ti | -0.077 | 8.73 | 4.546 | O | 6.993 | 4.879 | 1.484 | O | 19.017 | 10.651 | 0.766 |
| Ti | 10.443 | 8.806 | 4.423 | O | 3.269 | 6.806 | 5.186 | O | 17.573 | 10.65 | 3.438 |
| Ti | -0.08 | 12.573 | 4.546 | O | 4.71 | 6.805 | 2.57 | O | 12.329 | 8.76 | 3.383 |
| Ti | 10.47 | 12.607 | 4.511 | O | 1.169 | 4.884 | 5.906 | Ti | 8.983 | 12.568 | 1 |
| O | 10.008 | 1.044 | 2.456 | O | 4.79 | 4.883 | 4.64 | Ti | 5.19 | 14.492 | 4.579 |
| O | 20.559 | 1.047 | 2.484 | O | 8.452 | 6.799 | 0.828 | Ti | 6.56 | 14.49 | 1.433 |
| O | 10.032 | 4.839 | 2.462 | O | 6.995 | 6.784 | 3.636 | Ti | 2.757 | 12.562 | 4.951 |
| O | 20.56 | 4.891 | 2.483 | O | 1.742 | 4.895 | 3.474 | O | 6.981 | 12.571 | 1.442 |
| O | 10.053 | 8.743 | 2.423 | Ti | 19.556 | 4.888 | 1 | O | 3.284 | 14.497 | 5.19 |
| O | 20.558 | 8.729 | 2.482 | Ti | 15.743 | 6.84 | 4.511 | O | 4.727 | 14.495 | 2.518 |
| O | 10.021 | 12.583 | 2.457 | Ti | 17.114 | 6.809 | 1.373 | O | 1.165 | 12.576 | 5.906 |
| O | 20.559 | 12.571 | 2.484 | Ti | 13.358 | 4.886 | 4.886 | O | 4.774 | 12.579 | 4.615 |
| Ti | 8.984 | 1.04 | 0.988 | O | 17.522 | 4.887 | 1.362 | O | 8.448 | 14.489 | 0.765 |
| Ti | 5.194 | 2.971 | 4.587 | O | 13.834 | 6.812 | 5.145 | O | 7.018 | 14.5 | 3.488 |
| Ti | 6.56 | 2.963 | 1.439 | O | 15.279 | 6.823 | 2.45 | O | 1.74 | 12.57 | 3.472 |
| Ti | 2.759 | 1.048 | 4.952 | O | 15.295 | 4.894 | 4.509 | Ti | 19.556 | 12.572 | 1.001 |
| O | 6.975 | 1.043 | 1.403 | O | 19.016 | 6.808 | 0.764 | Ti | 15.753 | 14.486 | 4.516 |
| O | 3.29 | 2.965 | 5.191 | O | 17.568 | 6.814 | 3.437 | Ti | 17.116 | 14.493 | 1.379 |
| O | 4.727 | 2.975 | 2.527 | O | 12.339 | 4.858 | 3.443 | Ti | 13.312 | 12.57 | 4.896 |
| O | 1.167 | 1.05 | 5.906 | Ti | 8.979 | 8.727 | 0.989 | O | 17.523 | 12.573 | 1.36 |
| O | 4.782 | 1.046 | 4.609 | Ti | 5.206 | 10.649 | 4.599 | O | 13.854 | 14.497 | 5.133 |
| O | 8.444 | 2.972 | 0.753 | Ti | 6.574 | 10.649 | 1.524 | O | 15.286 | 14.496 | 2.46 |
| O | 7.018 | 2.909 | 3.492 | Ti | 2.761 | 8.72 | 4.95 | O | 11.74 | 12.59 | 5.861 |
| O | 1.743 | 1.048 | 3.472 | O | 7 | 8.726 | 1.521 | O | 15.345 | 12.573 | 4.536 |
| Ti | 19.556 | 1.046 | 1.002 | O | 3.27 | 10.652 | 5.182 | O | 19.019 | 14.493 | 0.766 |
| Ti | 15.749 | 2.949 | 4.516 | O | 4.723 | 10.644 | 2.546 | O | 17.578 | 14.494 | 3.441 |
| Ti | 17.115 | 2.967 | 1.378 | O | 1.167 | 8.729 | 5.905 | O | 12.31 | 12.571 | 3.418 |
| Ti | 13.314 | 1.043 | 4.907 | O | 4.783 | 8.729 | 4.636 | Ti | 8.041 | 2.915 | 4.957 |
| O | 17.525 | 1.046 | 1.36 | O | 8.447 | 10.642 | 0.796 | O | 8.586 | 1.028 | 5.206 |
| O | 13.845 | 2.973 | 5.167 | O | 7.009 | 10.709 | 3.589 | O | 6.473 | 2.965 | 5.933 |
| O | 15.281 | 2.959 | 2.456 | O | 1.739 | 8.723 | 3.472 | O | 10.058 | 2.942 | 4.538 |
| O | 11.744 | 1.046 | 5.873 | Ti | 19.556 | 8.731 | 0.999 | Ti | 18.589 | 2.972 | 4.917 |
| O | 15.342 | 1.054 | 4.54 | Ti | 15.75 | 10.656 | 4.514 | O | 19.115 | 1.05 | 5.16 |
| O | 19.018 | 2.967 | 0.766 | Ti | 17.114 | 10.651 | 1.375 | O | 16.995 | 2.98 | 5.874 |
| O | 17.571 | 2.967 | 3.441 | Ti | 13.288 | 8.737 | 4.884 | O | 20.606 | 2.97 | 4.563 |
| O | 12.304 | 1.055 | 3.429 | O | 17.521 | 8.73 | 1.352 | Ti | 8.059 | 6.851 | 5.243 |
| O | 8.58 | 4.863 | 5.127 | Ti | 11.849 | 4.879 | 1.36 | C | 11.187 | 11.702 | 11.946 |
| O | 6.406 | 6.828 | 6.043 | O | 12.247 | 6.804 | 1.343 | C | 11.851 | 10.646 | 12.58 |
| O | 10.086 | 6.795 | 4.447 | O | 13.737 | 4.884 | 0.738 | H | 12.2 | 8.501 | 12.194 |
| Ti | 18.585 | 6.808 | 4.915 | O | 15.859 | 6.801 | 0.015 | C | 11.095 | 13.039 | 12.358 |
| O | 19.112 | 4.889 | 5.156 | O | 10.56 | 4.898 | 0.03 | H | 12.391 | 10.781 | 13.515 |
| O | 16.991 | 6.807 | 5.869 | Ti | 3.741 | 10.643 | 1.051 | C | 11.013 | 14.202 | 12.73 |
| O | 20.605 | 6.807 | 4.567 | Ti | 1.289 | 8.725 | 1.412 | C | 10.936 | 15.588 | 13.155 |
| Ti | 8.035 | 10.674 | 5.125 | O | 1.699 | 10.646 | 1.39 | H | 9.891 | 15.903 | 13.295 |
| O | 8.559 | 8.747 | 5.002 | O | 3.203 | 8.727 | 0.816 | H | 11.469 | 15.739 | 14.106 |
| O | 6.4 | 10.652 | 5.992 | O | 5.343 | 10.633 | 0.133 | H | 11.39 | 16.249 | 12.4 |
| O | 10.07 | 10.671 | 4.497 | O | 0.034 | 8.729 | 0.049 |  |  |  |  |
| Ti | 18.588 | 10.649 | 4.916 | Ti | 14.273 | 10.654 | 0.969 |  |  |  |  |
| O | 19.112 | 8.728 | 5.155 | Ti | 11.834 | 8.728 | 1.32 |  |  |  |  |
| O | 17 | 10.65 | 5.873 | O | 12.242 | 10.651 | 1.305 |  |  |  |  |
| O | 20.608 | 10.65 | 4.566 | O | 13.739 | 8.727 | 0.712 |  |  |  |  |
| Ti | 8.037 | 14.487 | 4.958 | O | 15.857 | 10.657 | 0.017 |  |  |  |  |
| O | 8.591 | 12.59 | 5.198 | O | 10.548 | 8.714 | -0.008 |  |  |  |  |
| O | 6.461 | 14.49 | 5.926 | Ti | 3.718 | 14.49 | 1.04 |  |  |  |  |
| O | 10.06 | 14.496 | 4.52 | Ti | 1.29 | 12.562 | 1.411 |  |  |  |  |
| Ti | 18.591 | 14.495 | 4.919 | O | 1.697 | 14.491 | 1.388 |  |  |  |  |
| O | 19.115 | 12.573 | 5.157 | O | 3.197 | 12.566 | 0.808 |  |  |  |  |
| O | 17.002 | 14.493 | 5.876 | O | 5.304 | 14.483 | 0.081 |  |  |  |  |
| O | 20.609 | 14.496 | 4.566 | O | 0.035 | 12.567 | 0.05 |  |  |  |  |
| Ti | 3.718 | 2.974 | 1.048 | Ti | 14.274 | 14.492 | 0.98 |  |  |  |  |
| Ti | 1.289 | 1.047 | 1.411 | Ti | 11.828 | 12.57 | 1.359 |  |  |  |  |
| O | 1.697 | 2.97 | 1.391 | O | 12.242 | 14.494 | 1.339 |  |  |  |  |
| O | 3.192 | 1.047 | 0.806 | O | 13.739 | 12.575 | 0.746 |  |  |  |  |
| O | 5.306 | 2.979 | 0.089 | O | 15.856 | 14.494 | 0.024 |  |  |  |  |
| O | 0.034 | 1.048 | 0.05 | O | 10.556 | 12.569 | 0.019 |  |  |  |  |
| Ti | 14.275 | 2.957 | 0.974 | N | 8.926 | 10.279 | 7.297 |  |  |  |  |
| Ti | 11.828 | 1.039 | 1.366 | C | 9.415 | 9.465 | 7.983 |  |  |  |  |
| O | 12.244 | 2.964 | 1.332 | C | 10.066 | 8.385 | 8.624 |  |  |  |  |
| O | 13.736 | 1.043 | 0.745 | C | 10.02 | 7.098 | 7.833 |  |  |  |  |
| O | 15.858 | 2.964 | 0.02 | O | 9.001 | 6.964 | 7.058 |  |  |  |  |
| O | 10.563 | 1.042 | 0.019 | O | 10.951 | 6.268 | 7.991 |  |  |  |  |
| Ti | 3.754 | 6.81 | 1.06 | H | 11.389 | 5.451 | 6.787 |  |  |  |  |
| Ti | 1.288 | 4.9 | 1.412 | O | 11.688 | 4.945 | 5.925 |  |  |  |  |
| O | 1.695 | 6.81 | 1.387 | C | 10.771 | 8.456 | 9.799 |  |  |  |  |
| O | 3.198 | 4.893 | 0.812 | C | 10.996 | 9.538 | 10.695 |  |  |  |  |
| O | 5.36 | 6.813 | 0.169 | H | 11.248 | 7.514 | 10.088 |  |  |  |  |
| O | 0.033 | 4.892 | 0.05 | S | 10.422 | 11.171 | 10.475 |  |  |  |  |
| Ti | 14.274 | 6.814 | 0.969 | C | 11.744 | 9.439 | 11.878 |  |  |  |  |

Table S11. Coordinates of 2-cyano-3-(5-(prop-1-yn-1-yl)thiophen-2-yl)propanoic acid adsorbed onto a (TiO_2_)_64_ surface in a bidentate fashion.

| Ti | -0.146 | 1.03 | 4.59 | Ti | 8.92 | 4.872 | 1.09 | O | 17.459 | 8.715 | 1.398 |
| --- | --- | --- | --- | --- | --- | --- | --- | --- | --- | --- | --- |
| Ti | 10.41 | 1.016 | 4.594 | Ti | 5.059 | 6.804 | 4.578 | O | 13.794 | 10.634 | 5.184 |
| Ti | -0.156 | 4.869 | 4.59 | Ti | 6.529 | 6.798 | 1.646 | O | 15.221 | 10.632 | 2.495 |
| Ti | 10.394 | 4.841 | 4.603 | Ti | 2.674 | 4.853 | 5.009 | O | 11.658 | 8.724 | 5.843 |
| Ti | -0.156 | 8.715 | 4.584 | O | 6.919 | 4.875 | 1.541 | O | 15.287 | 8.715 | 4.573 |
| Ti | 10.444 | 8.773 | 4.426 | O | 3.181 | 6.786 | 5.217 | O | 18.956 | 10.637 | 0.809 |
| Ti | -0.147 | 12.561 | 4.587 | O | 4.616 | 6.81 | 2.633 | O | 17.516 | 10.639 | 3.48 |
| Ti | 10.414 | 12.556 | 4.588 | O | 1.088 | 4.861 | 5.96 | O | 12.268 | 8.735 | 3.427 |
| O | 9.946 | 1.028 | 2.538 | O | 4.698 | 4.867 | 4.691 | Ti | 8.919 | 12.561 | 1.076 |
| O | 20.501 | 1.032 | 2.529 | O | 8.379 | 6.801 | 0.894 | Ti | 5.139 | 14.49 | 4.651 |
| O | 9.954 | 4.871 | 2.549 | O | 6.883 | 6.706 | 3.721 | Ti | 6.501 | 14.476 | 1.51 |
| O | 20.495 | 4.874 | 2.528 | O | 1.674 | 4.854 | 3.522 | Ti | 2.686 | 12.552 | 5.004 |
| O | 10.034 | 8.718 | 2.432 | Ti | 19.489 | 4.871 | 1.046 | O | 6.916 | 12.562 | 1.516 |
| O | 20.493 | 8.714 | 2.525 | Ti | 15.688 | 6.798 | 4.559 | O | 3.223 | 14.485 | 5.244 |
| O | 9.956 | 12.572 | 2.532 | Ti | 17.05 | 6.793 | 1.421 | O | 4.669 | 14.476 | 2.591 |
| O | 20.5 | 12.555 | 2.527 | Ti | 13.253 | 4.882 | 4.955 | O | 1.098 | 12.566 | 5.953 |
| Ti | 8.92 | 1.033 | 1.074 | O | 17.461 | 4.871 | 1.407 | O | 4.716 | 12.568 | 4.693 |
| Ti | 5.139 | 2.959 | 4.655 | O | 13.785 | 6.802 | 5.184 | O | 8.383 | 14.481 | 0.844 |
| Ti | 6.501 | 2.954 | 1.517 | O | 15.22 | 6.801 | 2.497 | O | 6.964 | 14.489 | 3.573 |
| Ti | 2.69 | 1.026 | 5.007 | O | 11.676 | 4.868 | 5.932 | O | 1.68 | 12.558 | 3.518 |
| O | 6.91 | 1.035 | 1.501 | O | 15.275 | 4.877 | 4.584 | Ti | 19.494 | 12.56 | 1.045 |
| O | 3.221 | 2.94 | 5.256 | O | 18.954 | 6.793 | 0.809 | Ti | 15.687 | 14.478 | 4.565 |
| O | 4.666 | 2.958 | 2.593 | O | 17.513 | 6.793 | 3.483 | Ti | 17.051 | 14.48 | 1.426 |
| O | 1.101 | 1.029 | 5.954 | O | 12.25 | 4.891 | 3.482 | Ti | 13.262 | 12.554 | 4.951 |
| O | 4.73 | 1.034 | 4.675 | Ti | 8.92 | 8.722 | 1.026 | O | 17.463 | 12.56 | 1.403 |
| O | 8.384 | 2.957 | 0.852 | Ti | 5.159 | 10.67 | 4.66 | O | 13.792 | 14.477 | 5.188 |
| O | 6.961 | 2.961 | 3.578 | Ti | 6.513 | 10.632 | 1.616 | O | 15.225 | 14.482 | 2.507 |
| O | 1.684 | 1.031 | 3.521 | Ti | 2.673 | 8.741 | 5.002 | O | 11.686 | 12.562 | 5.921 |
| Ti | 19.494 | 1.032 | 1.047 | O | 6.941 | 8.713 | 1.618 | O | 15.283 | 12.557 | 4.577 |
| Ti | 15.682 | 2.955 | 4.567 | O | 3.196 | 10.642 | 5.231 | O | 18.958 | 14.48 | 0.813 |
| Ti | 17.049 | 2.951 | 1.428 | O | 4.625 | 10.612 | 2.614 | O | 17.516 | 14.481 | 3.486 |
| Ti | 13.258 | 1.031 | 4.96 | O | 1.093 | 8.717 | 5.954 | O | 12.249 | 12.543 | 3.477 |
| O | 17.463 | 1.032 | 1.404 | O | 4.719 | 8.693 | 4.74 | Ti | 7.985 | 2.976 | 5.048 |
| O | 13.786 | 2.955 | 5.19 | O | 8.379 | 10.63 | 0.875 | O | 8.53 | 1.036 | 5.27 |
| O | 15.222 | 2.953 | 2.509 | O | 6.9 | 10.73 | 3.653 | O | 6.402 | 2.964 | 6.009 |
| O | 11.681 | 1.027 | 5.933 | O | 1.675 | 8.728 | 3.515 | O | 10.007 | 2.948 | 4.597 |
| O | 15.278 | 1.033 | 4.587 | Ti | 19.486 | 8.715 | 1.043 | Ti | 18.52 | 2.948 | 4.968 |
| O | 18.956 | 2.952 | 0.812 | Ti | 15.69 | 10.634 | 4.558 | O | 19.055 | 1.03 | 5.207 |
| O | 17.513 | 2.949 | 3.488 | Ti | 17.052 | 10.64 | 1.42 | O | 16.938 | 2.954 | 5.926 |
| O | 12.242 | 1.026 | 3.49 | Ti | 13.253 | 8.718 | 4.931 | O | 20.544 | 2.95 | 4.607 |
| Ti | 8.125 | 6.924 | 5.155 | Ti | 14.212 | 6.795 | 1.015 | C | 10.504 | 7.662 | 13.524 |
| O | 8.556 | 4.87 | 5.298 | Ti | 11.771 | 4.875 | 1.435 | C | 10.479 | 6.339 | 13.063 |
| O | 6.292 | 6.742 | 6.136 | O | 12.183 | 6.8 | 1.36 | H | 10.048 | 5.31 | 11.162 |
| O | 9.98 | 6.814 | 4.601 | O | 13.671 | 4.871 | 0.8 | C | 10.776 | 8.137 | 14.817 |
| Ti | 18.522 | 6.793 | 4.963 | O | 15.797 | 6.782 | 0.062 | H | 10.695 | 5.491 | 13.711 |
| O | 19.05 | 4.872 | 5.203 | O | 10.486 | 4.876 | 0.104 | C | 10.968 | 8.563 | 15.948 |
| O | 16.94 | 6.796 | 5.921 | Ti | 3.682 | 10.627 | 1.102 | C | 11.201 | 9.086 | 17.281 |
| O | 20.546 | 6.793 | 4.607 | Ti | 1.219 | 8.716 | 1.452 | H | 10.349 | 9.698 | 17.616 |
| Ti | 7.997 | 10.633 | 5.196 | O | 1.631 | 10.63 | 1.426 | H | 11.342 | 8.271 | 18.007 |
| O | 8.435 | 8.732 | 4.703 | O | 3.133 | 8.704 | 0.846 | H | 12.099 | 9.724 | 17.302 |
| O | 6.331 | 10.629 | 6.054 | O | 5.291 | 10.616 | 0.208 |  |  |  |  |
| O | 10.023 | 10.643 | 4.571 | O | -0.034 | 8.712 | 0.091 |  |  |  |  |
| Ti | 18.525 | 10.64 | 4.961 | Ti | 14.214 | 10.64 | 1.013 |  |  |  |  |
| O | 19.052 | 8.715 | 5.198 | Ti | 11.774 | 8.718 | 1.327 |  |  |  |  |
| O | 16.943 | 10.636 | 5.918 | O | 12.182 | 10.637 | 1.346 |  |  |  |  |
| O | 20.547 | 10.637 | 4.606 | O | 13.689 | 8.717 | 0.751 |  |  |  |  |
| Ti | 7.982 | 14.486 | 5.046 | O | 15.798 | 10.65 | 0.06 |  |  |  |  |
| O | 8.535 | 12.554 | 5.273 | O | 10.473 | 8.715 | 0.006 |  |  |  |  |
| O | 6.402 | 14.485 | 6.005 | Ti | 3.663 | 14.474 | 1.108 |  |  |  |  |
| O | 9.998 | 14.48 | 4.606 | Ti | 1.228 | 12.547 | 1.456 |  |  |  |  |
| Ti | 18.524 | 14.479 | 4.966 | O | 1.637 | 14.477 | 1.435 |  |  |  |  |
| O | 19.055 | 12.559 | 5.202 | O | 3.141 | 12.552 | 0.869 |  |  |  |  |
| O | 16.941 | 14.477 | 5.923 | O | 5.249 | 14.476 | 0.153 |  |  |  |  |
| O | 20.548 | 14.48 | 4.604 | O | -0.026 | 12.556 | 0.095 |  |  |  |  |
| Ti | 3.664 | 2.956 | 1.108 | Ti | 14.209 | 14.482 | 1.03 |  |  |  |  |
| Ti | 1.228 | 1.03 | 1.459 | Ti | 11.769 | 12.562 | 1.422 |  |  |  |  |
| O | 1.638 | 2.956 | 1.434 | O | 12.179 | 14.479 | 1.4 |  |  |  |  |
| O | 3.139 | 1.033 | 0.872 | O | 13.675 | 12.565 | 0.799 |  |  |  |  |
| O | 5.252 | 2.963 | 0.157 | O | 15.791 | 14.483 | 0.072 |  |  |  |  |
| O | -0.025 | 1.032 | 0.097 | O | 10.486 | 12.559 | 0.09 |  |  |  |  |
| Ti | 14.207 | 2.953 | 1.033 | H | 6.199 | 7.303 | 6.925 |  |  |  |  |
| Ti | 11.768 | 1.033 | 1.436 | O | 8.583 | 7.597 | 7.099 |  |  |  |  |
| O | 12.179 | 2.955 | 1.41 | C | 8.822 | 8.758 | 7.614 |  |  |  |  |
| O | 13.667 | 1.034 | 0.803 | C | 9.263 | 8.808 | 9.028 |  |  |  |  |
| O | 15.789 | 2.95 | 0.074 | O | 8.698 | 9.887 | 7.035 |  |  |  |  |
| O | 10.494 | 1.037 | 0.098 | C | 9.507 | 7.656 | 9.743 |  |  |  |  |
| Ti | 3.674 | 6.799 | 1.094 | C | 9.418 | 10.106 | 9.589 |  |  |  |  |
| Ti | 1.222 | 4.88 | 1.458 | C | 9.899 | 7.492 | 11.1 |  |  |  |  |
| O | 1.629 | 6.794 | 1.439 | H | 9.373 | 6.727 | 9.182 |  |  |  |  |
| O | 3.138 | 4.885 | 0.86 | N | 9.548 | 11.166 | 10.074 |  |  |  |  |
| O | 5.298 | 6.809 | 0.236 | S | 10.121 | 8.785 | 12.255 |  |  |  |  |
| O | -0.029 | 4.875 | 0.096 | C | 10.133 | 6.249 | 11.711 |  |  |  |  |

Table S12. Coordinates of approximation of LW1 adsorbed onto a (TiO_2_)_64_ surface in a tridentate fashion.

| Ti | -0.079 | 1.047 | 4.546 | Ti | 8.977 | 4.883 | 0.993 | O | 13.846 | 10.653 | 5.135 |
| --- | --- | --- | --- | --- | --- | --- | --- | --- | --- | --- | --- |
| Ti | 10.478 | 1.074 | 4.517 | Ti | 5.243 | 6.81 | 4.612 | O | 15.28 | 10.646 | 2.452 |
| Ti | -0.075 | 4.889 | 4.545 | Ti | 6.58 | 6.807 | 1.587 | O | 11.696 | 8.739 | 5.811 |
| Ti | 10.347 | 4.964 | 4.424 | Ti | 2.762 | 4.906 | 4.954 | O | 15.336 | 8.73 | 4.535 |
| Ti | -0.077 | 8.73 | 4.546 | O | 6.993 | 4.879 | 1.484 | O | 19.017 | 10.651 | 0.766 |
| Ti | 10.443 | 8.806 | 4.423 | O | 3.269 | 6.806 | 5.186 | O | 17.573 | 10.65 | 3.438 |
| Ti | -0.08 | 12.573 | 4.546 | O | 4.71 | 6.805 | 2.57 | O | 12.329 | 8.76 | 3.383 |
| Ti | 10.47 | 12.607 | 4.511 | O | 1.169 | 4.884 | 5.906 | Ti | 8.983 | 12.568 | 1 |
| O | 10.008 | 1.044 | 2.456 | O | 4.79 | 4.883 | 4.64 | Ti | 5.19 | 14.492 | 4.579 |
| O | 20.559 | 1.047 | 2.484 | O | 8.452 | 6.799 | 0.828 | Ti | 6.56 | 14.49 | 1.433 |
| O | 10.032 | 4.839 | 2.462 | O | 6.995 | 6.784 | 3.636 | Ti | 2.757 | 12.562 | 4.951 |
| O | 20.56 | 4.891 | 2.483 | O | 1.742 | 4.895 | 3.474 | O | 6.981 | 12.571 | 1.442 |
| O | 10.053 | 8.743 | 2.423 | Ti | 19.556 | 4.888 | 1 | O | 3.284 | 14.497 | 5.19 |
| O | 20.558 | 8.729 | 2.482 | Ti | 15.743 | 6.84 | 4.511 | O | 4.727 | 14.495 | 2.518 |
| O | 10.021 | 12.583 | 2.457 | Ti | 17.114 | 6.809 | 1.373 | O | 1.165 | 12.576 | 5.906 |
| O | 20.559 | 12.571 | 2.484 | Ti | 13.358 | 4.886 | 4.886 | O | 4.774 | 12.579 | 4.615 |
| Ti | 8.984 | 1.04 | 0.988 | O | 17.522 | 4.887 | 1.362 | O | 8.448 | 14.489 | 0.765 |
| Ti | 5.194 | 2.971 | 4.587 | O | 13.834 | 6.812 | 5.145 | O | 7.018 | 14.5 | 3.488 |
| Ti | 6.56 | 2.963 | 1.439 | O | 15.279 | 6.823 | 2.45 | O | 1.74 | 12.57 | 3.472 |
| Ti | 2.759 | 1.048 | 4.952 | O | 15.295 | 4.894 | 4.509 | Ti | 19.556 | 12.572 | 1.001 |
| O | 6.975 | 1.043 | 1.403 | O | 19.016 | 6.808 | 0.764 | Ti | 15.753 | 14.486 | 4.516 |
| O | 3.29 | 2.965 | 5.191 | O | 17.568 | 6.814 | 3.437 | Ti | 17.116 | 14.493 | 1.379 |
| O | 4.727 | 2.975 | 2.527 | O | 12.339 | 4.858 | 3.443 | Ti | 13.312 | 12.57 | 4.896 |
| O | 1.167 | 1.05 | 5.906 | Ti | 8.979 | 8.727 | 0.989 | O | 17.523 | 12.573 | 1.36 |
| O | 4.782 | 1.046 | 4.609 | Ti | 5.206 | 10.649 | 4.599 | O | 13.854 | 14.497 | 5.133 |
| O | 8.444 | 2.972 | 0.753 | Ti | 6.574 | 10.649 | 1.524 | O | 15.286 | 14.496 | 2.46 |
| O | 7.018 | 2.909 | 3.492 | Ti | 2.761 | 8.72 | 4.95 | O | 11.74 | 12.59 | 5.861 |
| O | 1.743 | 1.048 | 3.472 | O | 7 | 8.726 | 1.521 | O | 15.345 | 12.573 | 4.536 |
| Ti | 19.556 | 1.046 | 1.002 | O | 3.27 | 10.652 | 5.182 | O | 19.019 | 14.493 | 0.766 |
| Ti | 15.749 | 2.949 | 4.516 | O | 4.723 | 10.644 | 2.546 | O | 17.578 | 14.494 | 3.441 |
| Ti | 17.115 | 2.967 | 1.378 | O | 1.167 | 8.729 | 5.905 | O | 12.31 | 12.571 | 3.418 |
| Ti | 13.314 | 1.043 | 4.907 | O | 4.783 | 8.729 | 4.636 | Ti | 8.041 | 2.915 | 4.957 |
| O | 17.525 | 1.046 | 1.36 | O | 8.447 | 10.642 | 0.796 | O | 8.586 | 1.028 | 5.206 |
| O | 13.845 | 2.973 | 5.167 | O | 7.009 | 10.709 | 3.589 | O | 6.473 | 2.965 | 5.933 |
| O | 15.281 | 2.959 | 2.456 | O | 1.739 | 8.723 | 3.472 | O | 10.058 | 2.942 | 4.538 |
| O | 11.744 | 1.046 | 5.873 | Ti | 19.556 | 8.731 | 0.999 | Ti | 18.589 | 2.972 | 4.917 |
| O | 15.342 | 1.054 | 4.54 | Ti | 15.75 | 10.656 | 4.514 | O | 19.115 | 1.05 | 5.16 |
| O | 19.018 | 2.967 | 0.766 | Ti | 17.114 | 10.651 | 1.375 | O | 16.995 | 2.98 | 5.874 |
| O | 17.571 | 2.967 | 3.441 | Ti | 13.288 | 8.737 | 4.884 | O | 20.606 | 2.97 | 4.563 |
| O | 12.304 | 1.055 | 3.429 | O | 17.521 | 8.73 | 1.352 | Ti | 8.059 | 6.851 | 5.243 |
| O | 8.58 | 4.863 | 5.127 | Ti | 11.849 | 4.879 | 1.36 | C | 11.187 | 11.702 | 11.946 |
| O | 6.406 | 6.828 | 6.043 | O | 12.247 | 6.804 | 1.343 | C | 11.851 | 10.646 | 12.58 |
| O | 10.086 | 6.795 | 4.447 | O | 13.737 | 4.884 | 0.738 | H | 12.2 | 8.501 | 12.194 |
| Ti | 18.585 | 6.808 | 4.915 | O | 15.859 | 6.801 | 0.015 | C | 11.095 | 13.039 | 12.358 |
| O | 19.112 | 4.889 | 5.156 | O | 10.56 | 4.898 | 0.03 | H | 12.391 | 10.781 | 13.515 |
| O | 16.991 | 6.807 | 5.869 | Ti | 3.741 | 10.643 | 1.051 | C | 11.013 | 14.202 | 12.73 |
| O | 20.605 | 6.807 | 4.567 | Ti | 1.289 | 8.725 | 1.412 | C | 10.932 | 15.673 | 13.181 |
| Ti | 8.035 | 10.674 | 5.125 | O | 1.699 | 10.646 | 1.39 | C | 11.579 | 16.033 | 14.393 |
| O | 8.559 | 8.747 | 5.002 | O | 3.203 | 8.727 | 0.816 | C | 10.196 | 16.557 | 12.348 |
| O | 6.4 | 10.652 | 5.992 | O | 5.343 | 10.633 | 0.133 | N | 11.589 | 17.285 | 14.939 |
| O | 10.07 | 10.671 | 4.497 | O | 0.034 | 8.729 | 0.049 | C | 12.328 | 15.119 | 15.221 |
| Ti | 18.588 | 10.649 | 4.916 | Ti | 14.273 | 10.654 | 0.969 | N | 10 | 17.887 | 12.589 |
| O | 19.112 | 8.728 | 5.155 | Ti | 11.834 | 8.728 | 1.32 | C | 9.552 | 16.171 | 11.115 |
| O | 17 | 10.65 | 5.873 | O | 12.242 | 10.651 | 1.305 | Zn | 10.62 | 18.95 | 14.231 |
| O | 20.608 | 10.65 | 4.566 | O | 13.739 | 8.727 | 0.712 | C | 12.327 | 17.196 | 16.104 |
| Ti | 8.037 | 14.487 | 4.958 | O | 15.857 | 10.657 | 0.017 | H | 12.478 | 14.069 | 15.012 |
| O | 8.591 | 12.59 | 5.198 | O | 10.548 | 8.714 | -0.008 | C | 12.791 | 15.839 | 16.28 |
| O | 6.461 | 14.49 | 5.926 | Ti | 3.718 | 14.49 | 1.04 | C | 9.239 | 18.366 | 11.538 |
| O | 10.06 | 14.496 | 4.52 | Ti | 1.29 | 12.562 | 1.411 | H | 9.558 | 15.175 | 10.695 |
| Ti | 18.591 | 14.495 | 4.919 | O | 1.697 | 14.491 | 1.388 | C | 8.96 | 17.291 | 10.615 |
| O | 19.115 | 12.573 | 5.157 | O | 3.197 | 12.566 | 0.808 | N | 11.395 | 20.054 | 15.778 |
| O | 17.002 | 14.493 | 5.876 | O | 5.304 | 14.483 | 0.081 | N | 9.806 | 20.655 | 13.428 |
| O | 20.609 | 14.496 | 4.566 | O | 0.035 | 12.567 | 0.05 | C | 12.597 | 18.25 | 16.985 |
| Ti | 3.718 | 2.974 | 1.048 | Ti | 14.274 | 14.492 | 0.98 | H | 13.393 | 15.491 | 17.107 |
| Ti | 1.289 | 1.047 | 1.411 | Ti | 11.828 | 12.57 | 1.359 | C | 8.804 | 19.687 | 11.376 |
| O | 1.697 | 2.97 | 1.391 | O | 12.242 | 14.494 | 1.339 | H | 8.387 | 17.387 | 9.704 |
| O | 3.192 | 1.047 | 0.806 | O | 13.739 | 12.575 | 0.746 | C | 11.203 | 21.391 | 16.014 |
| O | 5.306 | 2.979 | 0.089 | O | 15.856 | 14.494 | 0.024 | C | 12.157 | 19.58 | 16.821 |
| O | 0.034 | 1.048 | 0.05 | O | 10.556 | 12.569 | 0.019 | C | 9.824 | 21.913 | 13.973 |
| Ti | 14.275 | 2.957 | 0.974 | N | 8.926 | 10.279 | 7.297 | C | 9.076 | 20.747 | 12.264 |
| Ti | 11.828 | 1.039 | 1.366 | C | 9.415 | 9.465 | 7.983 | C | 13.42 | 17.943 | 18.201 |
| O | 12.244 | 2.964 | 1.332 | C | 10.066 | 8.385 | 8.624 | C | 7.983 | 20.002 | 10.16 |
| O | 13.736 | 1.043 | 0.745 | C | 10.02 | 7.098 | 7.833 | C | 11.862 | 21.777 | 17.234 |
| O | 15.858 | 2.964 | 0.02 | O | 9.001 | 6.964 | 7.058 | C | 10.471 | 22.278 | 15.183 |
| O | 10.563 | 1.042 | 0.019 | O | 10.951 | 6.268 | 7.991 | C | 12.454 | 20.655 | 17.735 |
| Ti | 3.754 | 6.81 | 1.06 | H | 11.389 | 5.451 | 6.787 | C | 9.092 | 22.827 | 13.136 |
| Ti | 1.288 | 4.9 | 1.412 | O | 11.688 | 4.945 | 5.925 | C | 8.628 | 22.105 | 12.076 |
| O | 1.695 | 6.81 | 1.387 | C | 10.771 | 8.456 | 9.799 | C | 14.823 | 18.054 | 18.165 |
| O | 3.198 | 4.893 | 0.812 | C | 10.996 | 9.538 | 10.695 | C | 12.803 | 17.54 | 19.401 |
| O | 5.36 | 6.813 | 0.169 | H | 11.248 | 7.514 | 10.088 | C | 6.58 | 19.898 | 10.196 |
| O | 0.033 | 4.892 | 0.05 | S | 10.422 | 11.171 | 10.475 | C | 8.602 | 20.409 | 8.963 |
| Ti | 14.274 | 6.814 | 0.969 | C | 11.744 | 9.439 | 11.878 | H | 11.869 | 22.776 | 17.645 |
| C | 10.38 | 23.633 | 15.593 | C | 10.752 | 27.911 | 17.97 |  |  |  |  |
| H | 13.039 | 20.56 | 18.638 | H | 11.384 | 25.88 | 18.164 |  |  |  |  |
| H | 8.957 | 23.88 | 13.338 | C | 10.042 | 28.876 | 17.207 |  |  |  |  |
| H | 8.038 | 22.455 | 11.242 | H | 8.868 | 29.125 | 15.391 |  |  |  |  |
| C | 15.596 | 17.767 | 19.3 | H | 11.243 | 28.197 | 18.891 |  |  |  |  |
| O | 15.347 | 18.452 | 16.97 | N | 9.958 | 30.186 | 17.612 |  |  |  |  |
| C | 13.567 | 17.251 | 20.54 | C | 10.604 | 30.616 | 18.843 |  |  |  |  |
| O | 11.441 | 17.459 | 19.358 | C | 9.224 | 31.155 | 16.81 |  |  |  |  |
| C | 5.808 | 20.194 | 9.064 | H | 11.69 | 30.458 | 18.81 |  |  |  |  |
| O | 6.055 | 19.496 | 11.39 | H | 10.423 | 31.68 | 18.989 |  |  |  |  |
| C | 7.838 | 20.708 | 7.825 | H | 10.21 | 30.083 | 19.718 |  |  |  |  |
| O | 9.963 | 20.486 | 9.005 | H | 8.167 | 30.881 | 16.707 |  |  |  |  |
| C | 10.303 | 24.798 | 15.949 | H | 9.273 | 32.13 | 17.295 |  |  |  |  |
| C | 14.954 | 17.37 | 20.471 | H | 9.649 | 31.256 | 15.803 |  |  |  |  |
| H | 16.675 | 17.85 | 19.276 |  |  |  |  |  |  |  |  |
| C | 16.761 | 18.584 | 16.858 |  |  |  |  |  |  |  |  |
| H | 13.097 | 16.94 | 21.464 |  |  |  |  |  |  |  |  |
| C | 10.748 | 17.055 | 20.536 |  |  |  |  |  |  |  |  |
| C | 6.451 | 20.595 | 7.894 |  |  |  |  |  |  |  |  |
| H | 4.729 | 20.115 | 9.087 |  |  |  |  |  |  |  |  |
| C | 4.64 | 19.37 | 11.502 |  |  |  |  |  |  |  |  |
| H | 8.309 | 21.021 | 6.902 |  |  |  |  |  |  |  |  |
| C | 10.658 | 20.894 | 7.83 |  |  |  |  |  |  |  |  |
| C | 10.216 | 26.15 | 16.366 |  |  |  |  |  |  |  |  |
| H | 15.549 | 17.146 | 21.351 |  |  |  |  |  |  |  |  |
| H | 17.15 | 19.338 | 17.552 |  |  |  |  |  |  |  |  |
| H | 17.27 | 17.63 | 17.038 |  |  |  |  |  |  |  |  |
| H | 16.95 | 18.905 | 15.833 |  |  |  |  |  |  |  |  |
| H | 11.04 | 16.046 | 20.849 |  |  |  |  |  |  |  |  |
| H | 10.92 | 17.754 | 21.363 |  |  |  |  |  |  |  |  |
| H | 9.69 | 17.06 | 20.275 |  |  |  |  |  |  |  |  |
| H | 5.856 | 20.825 | 7.015 |  |  |  |  |  |  |  |  |
| H | 4.136 | 20.328 | 11.326 |  |  |  |  |  |  |  |  |
| H | 4.247 | 18.621 | 10.805 |  |  |  |  |  |  |  |  |
| H | 4.45 | 19.045 | 12.525 |  |  |  |  |  |  |  |  |
| H | 10.484 | 20.2 | 6.999 |  |  |  |  |  |  |  |  |
| H | 10.37 | 21.906 | 7.523 |  |  |  |  |  |  |  |  |
| H | 11.716 | 20.884 | 8.091 |  |  |  |  |  |  |  |  |
| C | 9.51 | 27.109 | 15.607 |  |  |  |  |  |  |  |  |
| C | 10.833 | 26.592 | 17.557 |  |  |  |  |  |  |  |  |
| C | 9.422 | 28.431 | 16.009 |  |  |  |  |  |  |  |  |
| H | 9.024 | 26.802 | 14.685 |  |  |  |  |  |  |  |  |

**References:**

[1] N. Duvva, S. Prasanthkumar, L. J. S. E. Giribabu, **2019**, *184*, 620.

[2] S. Li, Y. Zhang, S. Mei, X. Kong, M. Yang, Z. Hu, W. Wu, J. He, H. J. A. A. E. M. Tan, **2021**, *4*, 9267.

[3] C.-Y. Lin, C.-F. Lo, L. Luo, H.-P. Lu, C.-S. Hung, E. W.-G. Diau, *The Journal of Physical Chemistry C* **2009**, *113*, 755.

[4] C.-H. Wu, T.-Y. Pan, S.-H. Hong, C.-L. Wang, H.-H. Kuo, Y.-Y. Chu, E. W.-G. Diau, C.-Y. Lin, *Chemical Communications* **2012**, *48*, 4329.

[5] H.-P. Lu, C.-L. Mai, C.-Y. Tsia, S.-J. Hsu, C.-P. Hsieh, C.-L. Chiu, C.-Y. Yeh, E. W.-G. Diau, *Physical Chemistry Chemical Physics* **2009**, *11*, 10270.

[6] L. Cabau, C. V. Kumar, A. Moncho, J. N. Clifford, N. López, E. Palomares, *Energy & Environmental Science* **2015**, *8*, 1368.

[7] C.-H. Wu, M.-C. Chen, P.-C. Su, H.-H. Kuo, C.-L. Wang, C.-Y. Lu, C.-H. Tsai, C.-C. Wu, C.-Y. Lin, *Journal of Materials Chemistry A* **2014**, *2*, 991.

[8] J. Lu, X. Xu, K. Cao, J. Cui, Y. Zhang, Y. Shen, X. Shi, L. Liao, Y. Cheng, M. Wang, *Journal of Materials Chemistry A* **2013**, *1*, 10008.

[9] J. Lu, H. Li, S. Liu, Y.-C. Chang, H.-P. Wu, Y. Cheng, E. W.-G. Diau, M. Wang, *Physical Chemistry Chemical Physics* **2016**, *18*, 6885.

[10] Y. C. Chang, C. L. Wang, T. Y. Pan, S. H. Hong, C. M. Lan, H. H. Kuo, C. F. Lo, H. Y. Hsu, C. Y. Lin, E. W. Diau, *Chem. Commun.* **2011**, *47*, 8910.

[11] M. Tanaka, S. Hayashi, S. Eu, T. Umeyama, Y. Matano, H. Imahori, *Chemical communications* **2007**, 2069.

[12] S. Hayashi, Y. Matsubara, S. Eu, H. Hayashi, T. Umeyama, Y. Matano, H. Imahori, *Chemistry letters* **2008**, *37*, 846.

[13] S. Hayashi, M. Tanaka, H. Hayashi, S. Eu, T. Umeyama, Y. Matano, Y. Araki, H. Imahori, *The Journal of Physical Chemistry C* **2008**, *112*, 15576.

[14] M. Cariello, S. M. Abdalhadi, P. Yadav, J.-D. Decoppet, S. M. Zakeeruddin, M. Grätzel, A. Hagfeldt, G. J. D. T. Cooke, **2018**, *47*, 6549.

[15] S. Eu, S. Hayashi, T. Umeyama, A. Oguro, M. Kawasaki, N. Kadota, Y. Matano, H. Imahori, *J. Phys. Chem. C* **2007**, *111*, 3528.

[16] C.-P. Hsieh, H.-P. Lu, C.-L. Chiu, C.-W. Lee, S.-H. Chuang, C.-L. Mai, W.-N. Yen, S.-J. Hsu, E. W.-G. Diau, C.-Y. Yeh, *Journal of Materials Chemistry* **2010**, *20*, 1127.

[17] H. Song, X. Li, H. Ågren, Y. Xie, *Dyes and Pigments* **2017**, *137*, 421.

[18] M. J. Lee, K. D. Seo, H. M. Song, M. S. Kang, Y. K. Eom, H. S. Kang, H. K. Kim, *Tetrahedron Letters* **2011**, *52*, 3879.

[19] Y. Tang, Y. Wang, X. Li, H. Ågren, W.-H. Zhu, Y. Xie, *ACS Applied Materials & Interfaces* **2015**, *7*, 27976.

[20] H.-L. Jia, M.-D. Zhang, Z.-M. Ju, H.-G. Zheng, X.-H. Ju, *Journal of Materials Chemistry A* **2015**, *3*, 14809.

[21] H.-L. Jia, M.-D. Zhang, W. Yan, X.-H. Ju, H.-G. Zheng, *Journal of Materials Chemistry A* **2016**, *4*, 11782.

[22] Y. Xie, Y. Tang, W. Wu, Y. Wang, J. Liu, X. Li, H. Tian, W.-H. Zhu, *Journal of the American Chemical Society* **2015**, *137*, 14055.

[23] Y. Lu, H. Song, X. Li, H. Ågren, Q. Liu, J. Zhang, X. Zhang, Y. Xie, *ACS applied materials & interfaces* **2019**, *11*, 5046.

[24] Y. Liu, N. Xiang, X. Feng, P. Shen, W. Zhou, C. Weng, B. Zhao, S. Tan, *Chemical communications* **2009**, 2499.

[25] Y. Wang, L. Xu, X. Wei, X. Li, H. Ågren, W. Wu, Y. Xie, *New Journal of Chemistry* **2014**, *38*, 3227.

[26] N. Duvva, S. Gangada, R. Chitta, L. Giribabu, **2020**, *24*, 1189.

[27] P. S. Gangadhar, S. Gonuguntla, S. Madanaboina, N. Islavath, U. Pal, L. J. J. o. P. Giribabu, P. A. Chemistry, **2020**, *392*, 112408.

[28] G. Yang, Y. Tang, X. Li, H. Ågren, Y. Xie, *ACS applied materials & interfaces* **2017**, *9*, 36875.

[29] H. L. Song, W. Q. Tang, S. L. Zhao, Q. Y. Liu, Y. S. Xie, *Dyes Pigm.* **2018**, *155*, 323.

[30] A. S. Hart, B. K. Chandra, H. B. Gobeze, L. R. Sequeira, F. D'Souza, *ACS Appl Mater Interfaces* **2013**, *5*, 5314.

[31] T. Wei, X. Sun, X. Li, H. Ågren, Y. Xie, *ACS Applied Materials & Interfaces* **2015**, *7*, 21956.

[32] T. Higashino, Y. Fujimori, K. Sugiura, Y. Tsuji, S. Ito, H. Imahori, *Angewandte Chemie* **2015**, *127*, 9180.

[33] Y. Wang, B. Chen, W. Wu, X. Li, W. Zhu, H. Tian, Y. Xie, *Angewandte Chemie* **2014**, *126*, 10955.

[34] S. Chang, H. Wang, Y. Hua, Q. Li, X. Xiao, W.-K. Wong, W. Y. Wong, X. Zhu, T. Chen, *Journal of Materials Chemistry A* **2013**, *1*, 11553.

[35] M. J. Griffith, K. Sunahara, P. Wagner, K. Wagner, G. G. Wallace, D. L. Officer, A. Furube, R. Katoh, S. Mori, A. J. Mozer, *Chemical Communications* **2012**, *48*, 4145.

[36] H. He, A. Gurung, L. Si, A. G. Sykes, *Chemical Communications* **2012**, *48*, 7619.

[37] D. Koteshwar, S. Prasanthkumar, S. P. Singh, T. H. Chowdhury, I. Bedja, A. Islam, L. J. M. C. F. Giribabu, **2022**, *6*, 580.

[38] W. Zhou, B. Zhao, P. Shen, S. Jiang, H. Huang, L. Deng, S. Tan, *Dyes and Pigments* **2011**, *91*, 404.

[39] C. Y. Lee, C. She, N. C. Jeong, J. T. Hupp, *Chemical communications* **2010**, *46*, 6090.

[40] M. S. Kang, S. H. Kang, S. G. Kim, I. T. Choi, J. H. Ryu, M. J. Ju, D. Cho, J. Y. Lee, H. K. Kim, *Chemical Communications* **2012**, *48*, 9349.

[41] S. H. Kang, I. T. Choi, M. S. Kang, Y. K. Eom, M. J. Ju, J. Y. Hong, H. S. Kang, H. K. Kim, *Journal of Materials Chemistry A* **2013**, *1*, 3977.

[42] M. S. Kang, I. T. Choi, Y. W. Kim, B. S. You, S. H. Kang, J. Y. Hong, M. J. Ju, H. K. Kim, *Journal of Materials Chemistry A* **2013**, *1*, 9848.

[43] A. Yella, H. W. Lee, H. N. Tsao, C. Yi, A. K. Chandiran, M. K. Nazeeruddin, E. W. Diau, C. Y. Yeh, S. M. Zakeeruddin, M. Gratzel, *Science* **2011**, *334*, 629.

[44] T. Ripolles-Sanchis, B. C. Guo, H. P. Wu, T. Y. Pan, H. W. Lee, S. R. Raga, F. Fabregat-Santiago, J. Bisquert, C. Y. Yeh, E. W. Diau, *Chem. Commun.* **2012**, *48*, 4368.

[45] N. Masi Reddy, T.-Y. Pan, Y. Christu Rajan, B.-C. Guo, C.-M. Lan, E. Wei-Guang Diau, C.-Y. Yeh, *Physical Chemistry Chemical Physics* **2013**, *15*, 8409.

[46] A. Yella, C. L. Mai, S. M. Zakeeruddin, S. N. Chang, C. H. Hsieh, C. Y. Yeh, M. Grätzel, *Angewandte Chemie* **2014**, *126*, 3017.

[47] S.-L. Wu, H.-P. Lu, H.-T. Yu, S.-H. Chuang, C.-L. Chiu, C.-W. Lee, E. W.-G. Diau, C.-Y. Yeh, *Energy & Environmental Science* **2010**, *3*, 949.

[48] M. Badertscher, K. Bischofberger, M. E. Munk, E. Pretsch, *J. Chem. Inf. Comput. Sci.* **2001**, *41*, 889.

[49] M. J. Frisch, G. W. Trucks, H. B. Schlegel, G. E. Scuseria, M. A. Robb, J. R. Cheeseman, G. Scalmani, V. Barone, G. A. Petersson, H. Nakatsuji, X. Li, M. Caricato, A. V. Marenich, J. Bloino, B. G. Janesko, R. Gomperts, B. Mennucci, H. P. Hratchian, J. V. Ortiz, A. F. Izmaylov, J. L. Sonnenberg, Williams, F. Ding, F. Lipparini, F. Egidi, J. Goings, B. Peng, A. Petrone, T. Henderson, D. Ranasinghe, V. G. Zakrzewski, J. Gao, N. Rega, G. Zheng, W. Liang, M. Hada, M. Ehara, K. Toyota, R. Fukuda, J. Hasegawa, M. Ishida, T. Nakajima, Y. Honda, O. Kitao, H. Nakai, T. Vreven, K. Throssell, J. A. Montgomery Jr., J. E. Peralta, F. Ogliaro, M. J. Bearpark, J. J. Heyd, E. N. Brothers, K. N. Kudin, V. N. Staroverov, T. A. Keith, R. Kobayashi, J. Normand, K. Raghavachari, A. P. Rendell, J. C. Burant, S. S. Iyengar, J. Tomasi, M. Cossi, J. M. Millam, M. Klene, C. Adamo, R. Cammi, J. W. Ochterski, R. L. Martin, K. Morokuma, O. Farkas, J. B. Foresman, D. J. Fox, Gaussian 16 Rev. C.01. Wallingford, CT, **2016**.

[50] A. D. Becke, *J. Chem. Phys.* **1993**, *98*, 5648.

[51] G. Petersson, M. A. J. T. J. o. c. p. Al‐Laham, **1991**, *94*, 6081.

[52] M. Cossi, N. Rega, G. Scalmani, V. J. J. o. c. c. Barone, **2003**, *24*, 669.

[53] N. M. O'boyle, A. L. Tenderholt, K. M. J. J. o. c. c. Langner, **2008**, *29*, 839.

[54] B. J. T. J. o. c. p. Delley, **1990**, *92*, 508.

[55] **2000**, *113*, 7756.

[56] J. P. Perdew, K. Burke, M. J. P. r. l. Ernzerhof, **1996**, *77*, 3865.

[57] J. P. Perdew, J. A. Chevary, S. H. Vosko, K. A. Jackson, M. R. Pederson, D. J. Singh, C. J. P. r. B. Fiolhais, **1992**, *46*, 6671.

[58] S. M. Lundberg, B. Nair, M. S. Vavilala, M. Horibe, M. J. Eisses, T. Adams, D. E. Liston, D. K.-W. Low, S.-F. Newman, J. Kim, S.-I. Lee, *Nature Biomedical Engineering* **2018**, *2*, 749.

[59] Lundberg, Scott, S.-I. Lee, *Adv Neural Inf Process Syst* **2017**, 4765.

[60] K. Sharma, V. Sharma, S. S. Sharma, *Nanoscale research letters* **2018**, *13*, 1.

[61] T.-F. Lu, W. Li, F.-Q. Bai, R. Jia, J. Chen, H.-X. J. J. o. M. C. A. Zhang, **2017**, *5*, 15567.

[62] N. Fuke, L. B. Hoch, A. Y. Koposov, V. W. Manner, D. J. Werder, A. Fukui, N. Koide, H. Katayama, M. J. A. N. Sykora, **2010**, *4*, 6377.

[63] C.-C. Chiu, Y.-C. Sheng, W.-J. Lin, R. Juwita, C.-J. Tan, H.-H. G. J. A. o. Tsai, **2018**, *3*, 433.

[64] N. M. O'Boyle, A. L. Tenderholt, K. M. Langner, *J. Comput. Chem.* **2008**, *29*, 839.

[65] R. Katoh, A. Furube, T. Yoshihara, K. Hara, G. Fujihashi, S. Takano, S. Murata, H. Arakawa, M. J. T. J. o. P. C. B. Tachiya, **2004**, *108*, 4818.

[66] K. Chaitanya, X.-H. Ju, B. M. J. R. A. Heron, **2014**, *4*, 26621.

[67] J.-L. J. M. H. Bredas, **2014**, *1*, 17.

[68] R. A. Marcus, *J. Chem. Phys.* **1965**, *43*, 679.

[69] R. A. Marcus, N. Sutin, *Biochim. Biophys. Acta, Rev. Bioenerg.* **1985**, *811*, 265.

[70] O. Lopez-Estrada, H. G. Laguna, C. Barrueta-Flores, C. J. A. o. Amador-Bedolla, **2018**, *3*, 2130.

[71] G. Ke, Q. Meng, T. Finley, T. Wang, W. Chen, W. Ma, Q. Ye, T.-Y. J. A. i. n. i. p. s. Liu, **2017**, *30*.

[72] A. Dongare, R. Kharde, A. D. J. I. J. o. E. Kachare, I. Technology, **2012**, *2*, 189.

[73] S. Albawi, T. A. Mohammed, S. Al-Zawi, in *2017 international conference on engineering and technology (ICET)* Ieee, **2017**, 1-6.
